# The expansion of chemical space in 1826 and in the 1840s prompted the convergence to the periodic system

**DOI:** 10.1073/pnas.2119083119

**Published:** 2022-07-22

**Authors:** Wilmer Leal, Eugenio J. Llanos, Andrés Bernal, Peter F. Stadler, Jürgen Jost, Guillermo Restrepo

**Affiliations:** ^a^Bioinformatics Group, Department of Computer Science, Universität Leipzig, 04107 Leipzig, Germany;; ^b^Max Planck Institute for Mathematics in the Sciences, 04103 Leipzig, Germany;; ^c^Corporación SCIO, 111321 Bogotá, Colombia;; ^d^Departamento de Ciencias Básicas, Universidad Jorge Tadeo Lozano, 110311 Bogotá, Colombia;; ^e^Interdisciplinary Center for Bioinformatics, Universität Leipzig, 04107 Leipzig, Germany;; ^f^Institute for Theoretical Chemistry, University of Vienna, 1090 Vienna, Austria;; ^g^Facultad de Ciencias, Universidad Nacional de Colombia, Sede Bogotá, Colombia;; ^h^The Santa Fe Institute, Santa Fe, NM 87501

**Keywords:** chemistry, periodic system, periodic table, chemical space, computational history

## Abstract

The periodic system, which intertwines order and similarity among chemical elements, arose from knowledge about substances constituting the chemical space. Little is known, however, about how the expansion of the space contributed to the emergence of the system—formulated in the 1860s. Here, we show by analyzing the space between 1800 and 1869 that after an unstable period culminating around 1826, chemical space led the system to converge to a backbone structure clearly recognizable in the 1840s. Hence, the system was already encoded in the space for about two and half decades before its formulation. Chemical events in 1826 and in the 1840s were driven by the discovery of new forms of combination standing the test of time. Emphasis of the space upon organic chemicals after 1830 prompted the recognition of relationships among elements participating in the organic turn and obscured some of the relationships among transition metals. To account for the role of nineteenth century atomic weights upon the system, we introduced an algorithm to adjust the space according to different sets of weights, which allowed for estimating the resulting periodic systems of chemists using one or the other weights. By analyzing these systems, from Dalton up to Mendeleev, Gmelin’s atomic weights of 1843 produce systems remarkably similar to that of 1869, a similarity that was reinforced by the atomic weights on the years to come. Although our approach is computational rather than historical, we hope it can complement other tools of the history of chemistry.

## Significance

The number and diversity of substances constituting the chemical space triggered, in two important steps, the convergence of the periodic system toward a stable backbone structure eventually unveiled in the 1860s. The first step occurred in 1826, and the second was between 1835 and 1845. Interestingly, the salient features of the periodic system of the 1860s can be detected as early as the 1840s, even when considering the effect of disagreement regarding the determination of atomic weights. The methods presented here become instrumental to study the further evolution of the periodic system and to ponder its current shape.

Meyer’s and Mendeleev’s periodic systems culminated a series of attempts to classify and order chemical elements (1) through the knowledge of their compounds (2, 3), which led to the systems of chemical elements (SCEs) of the 1860s. By finding the smallest common combining weight of a large set of compounds containing a reference element, atomic weights were determined and used to order elements (4). Likewise, chemical resemblance among elements was mainly determined on the basis of similarities of empirical and molecular formulae (2, 3). Thus, SCEs refer not just to chemical elements but to substances in general.

Every discovered substance enlarges the set of known chemicals, which we call the chemical space (5). Given the central role of this space for the formulation of the SCE, every discovery of new elements and compounds may affect the SCE by introducing or perturbing similarities among chemical elements or by affecting the ordering of their atomic weights. Therefore, we wondered how the evolution of the chemical space affected the SCE.

Historians have found that the ripe moment for formulating the system came in the 1860s (6, 7), thanks largely to the normalization of molecular formulae through the standardized set of atomic weights resulting from the 1860 Karlsruhe conference. We further wondered whether the chemical space was mature enough in the 1860s to prompt the formulation of the SCE.

In this contribution, we used chemical information from the Reaxys database to gain insight into the interplay of the expanding chemical space and the emergence of the SCE. We were interested in determining whether the growth of the chemical space led to SCEs that either diverged or converged and whether these processes occurred in a cumulative fashion or rather, through sudden events. Likewise, we investigated the role that research on atomic weights played in those processes leading to the emergence of the SCE.

## Evolution of the Chemical Space (1800 to 1868)

Gmelin’s and Beilstein’s handbooks, initiated in the nineteenth century, gather records of extractions, synthesis, and properties of substances (5, 8). Nowadays, Reaxys, a large electronic database of chemical information that merges these two handbooks plus several other sources of chemical information, constitutes a suitable corpus for studies on the evolution of chemistry (5, 8).

**Fig. 1.**
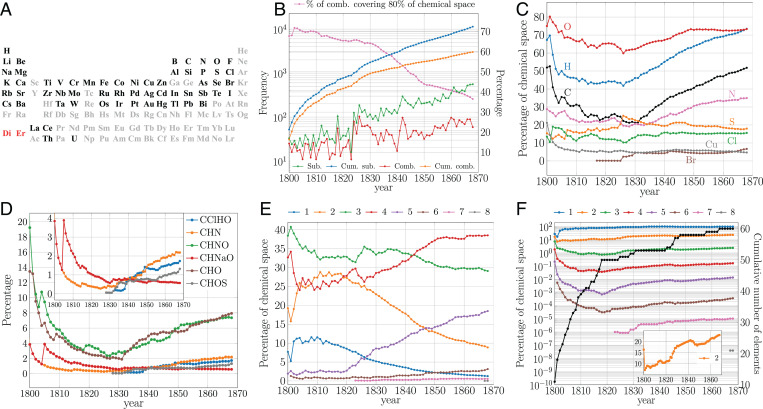
Chemical elements, growth, and diversity of the chemical space up to 1869. (*A*) Current SCE depicting elements known by 1869 (black), undiscovered elements (gray), and mixtures that were thought to be elements (red) (*SI Appendix*). Elements in black were considered in this study. (*B*) Absolute (left axis [l.a.]) and cumulative values (right axis [r.a.]) of new substances and combinations. (*C*) Percentage of chemical space spanned by some elements. These percentages are nonadditive because a single substance adds to each one of its elements (e.g., H_2_O contributes to both H and O counts). (*D*) Percentage of chemical space spanned by different combinations. (*E*) Percentage of chemical space spanned by substances made of *n* elements. After 1811, the number of uncombined forms (unary substances) in which elements appeared exceeded the number of known elements as a consequence of the allotropic forms and polymorphs of elements. For instance, by 1868, sulfur had nine uncombined forms. (*F*) Cumulative number of elements (r.a.) and percentage of theoretical combinations of different sizes actually observed (l.a.) (*Materials and Methods*). *Inset*: corresponding plot for binary combinations in linear scale.

We collected records from Reaxys* (January 2017) from 1771 up to 1868: that is, 2 mo before the publication of the first Mendeleev SCEs (9). These amounted to 11,356 substances involved in 21,521 single-step reactions (*Materials and Methods*), mainly reported in Gmelin’s handbook and gathered from leading nineteenth century journals (10). These substances span a growing number of elements over time from 9 in 1800 up to 60 in 1868 (Fig. 1*A*) (https://mchem.bioinf.uni-leipzig.de/1868/main.html). The most complete system was formulated by Mendeleev (11), including the 60 elements in Fig. 1*A* plus Er, Yt, and Di (12). Nonetheless, we excluded these three elements because of their unreliable information by 1869; Yt [currently Y (13)] first reported reaction dates back to 1872. Er and Di were later found to be mixtures of other elements (14) (*SI Appendix*).

Chemists expanded the chemical space at an exponential rate from a handful of new substances in 1800 up to the 11,000 of 1868 (5) (Fig. 1*B*). From each substance, we extracted its element combination: that is, HOS for H_2_SO_4_. We used the number of combinations as a measure of diversity. Fig. 1*B* shows that although combinations also grew exponentially (5), unlike substances their growth was reduced after 1830, indicating decreasing diversity. By the end of the period, 36% of the combinations covered 80% of the chemical space, while at the beginning of the nineteenth century, the same percentage of space was spanned by 71% of the combinations (Fig. 1*B*). Fig. 1*C* and *D* provides further details about the 1830 turning point. At the dawn of the century, the chemical space was mainly populated by compounds of C, H, O, and N; then, during the first quarter of the century, chemists found new combinations, which reduced the percentage of chemical space spanned by each combination (Fig. 1*D* and *SI Appendix*, Fig. S1). This was a period when the numbers of new substances and of combinations grew hand in hand (Fig. 1*B*). A minimum was reached for CHO and CHNO compounds around 1830. Afterward, there was again a clear emphasis on CHO and CHNO (Fig. 1*D*), which resulted in a less rapid production of new combinations (Fig. 1*B*). More CHO and CHNO substances distributed over a slow-growing number of combinations increased the space spanned by these combinations (Fig. 1*D* and *SI Appendix*, Fig. S2). This is clearly a consequence of the organic revolution (4, 15); before 1830, most new combinations were metallic, while afterward, most were organic (Fig. 1*D* and *SI Appendix*, Figs. S1 and S3 and Table S1). The importance of organic chemistry after 1830 is observed in *SI Appendix*, Figs. S4 and S5, where substances containing typical organic chemistry molecular fragments skyrocketed, in contrast with those containing inorganic ones.

Another attribute of a combination is its size: that is, the number of elements present in it. The theoretical number of combinations depends on the available elements. Thus, by 1800, with 11 elements, there were 2,036 possible combinations (*Materials and Methods*), which grew up to $1.15\times 10^{18}$ by 1868 with 60 elements. We found that despite the growth of new combinations (Fig. 1*B*), chemists reported compounds of no more than eight elements (Fig. 1*E*). During the first quarter of the nineteenth century, the chemical space was mainly populated by compounds of size 2 to 3, presumably due to the prevalence of dualism in chemical theories (15). Afterward, there was a surge in the number of larger combinations involving four to five elements, mostly organic.

By analyzing how close chemists were to actually realizing the theoretical combinations of different sizes, we found that during the first years of the nineteenth century, when a rapid discovery of elements took place (Fig. 1*F*), the number of theoretical combinations rose, causing a rapid drop of the proportion of realized combinations. Once the discovery of new elements slowed around 1820, more combinations were actually observed, increasing the proportion of theoretical combinations realized (Fig. 1*F*). In the mid-1840s came another batch of new elements, reducing again the proportion of realized combinations, which coincides with a strong drop in the number of new substances from 300 in 1842 to 163 in 1846 (Fig. 1*B*). After a decade, chemists were again discovering more combinations and increasing this proportion. As expected, given its relatively small number, binary combinations were always closer to their theoretical possibilities than combinations of more elements. By 1825, after the stabilization of the number of elements, about 13% of the theoretical number of binary compounds was reported (Fig. 1*F*, *Inset*), a growing percentage not even affected by the emphasis on compounds of three and four elements (Fig. 1*E*). In fact, by 1868 about 23% of the possible binary compounds (made with combinations of 60 elements) were already known (Fig. 1*F*, *Inset*).

## The Chemical Space from Which the Periodic System Arose

We analyzed from two perspectives, one contemporary or presentist and another historical or retrodictive, the effect of the evolution of the chemical space upon the ordering and similarity among chemical elements, which constitutes the foundations of the SCE (1).

The presentist approach “sees” the chemical space of the nineteenth century through the eyes of twenty-first century chemistry. Here, nineteenth century formulae (for example, Dalton’s OH for water) are replaced by their contemporary versions. Reaxys data suit this approach. This approach was designed to determine whether the ordering and similarity among chemical elements diverged or converged as the chemical space expanded. As solving these questions requires setting up a framework of reference, we selected the one provided by the large corpus of knowledge on the chemical space the chemical community has accumulated so far. We note that this approach may be regarded as a Whiggish account from a historical perspective, as it assesses the past from a present stance (16).

In order to analyze the role of the different sets of atomic weights developed before the formulation of the SCE, we designed the retrodictive approach, which considers the evolution of the chemical space as historically witnessed. It acknowledges the historical construction of consent on atomic weights and its associated formulae. Therefore, when analyzing the chemical space (for instance, of 1810), it attempts to use the formulae proposed by the leading chemists of that time. This approach allows for studying possible similarity and ordering relationships among chemical elements according to several nineteenth century chemists. It also turns instrumental to determine whether those relationships diverged or converged as atomic theory evolved.

### Presentist Approach to the Evolution of the SCEs.

**Fig. 2.**
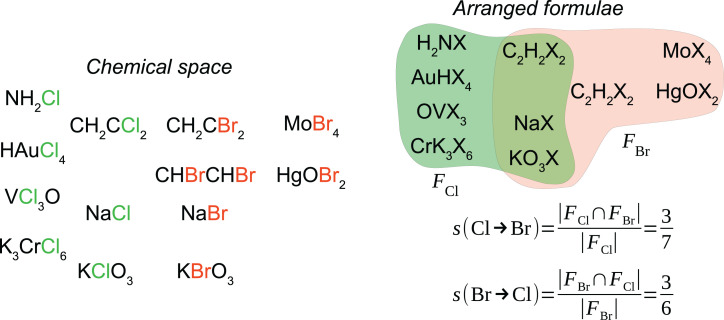
Similarity among chemical elements. Toy chemical space of 13 substances. Each compound provides an arranged formula for an element in the given formula when Cl or Br is replaced by X and the elements are lexicographically ordered. Arranged formulae of element X are gathered in $F_{\text{X}}$, which is a multiset as elements may appear more than once (e.g., C_2_H_2_X_2_ appears twice in $F_{\text{Br}}$) (*SI Appendix*). The similarity of element *x* to element *y* is given by s(x→y), which is the probability of *x* having a common arranged formula with *y*. In chemical terms, it is a measure of substitutability. This similarity is an asymmetric relation (17) [e.g., *s*(Br → Cl) > *s*(Cl → Br)]. For instance, by 1869, we have *s*(Br → Cl) = 344/659 = 0.52, while *s*(Cl → Br) = 349/1,556 = 0.22 (https://mchem.bioinf.uni-leipzig.de/1868/main.html). This means that Br could be substituted by Cl to obtain a known compound in roughly half of Br combinations, whereas Cl could be substituted by Br in about one-fourth of those of Cl. This similarity measure generalizes that presented in ref. 18.

Fig. 2 explains our methodology to quantifying similarity among chemical elements (*SI Appendix*), which is based on Mendeleev’s idea that “the elements, which are most chemically analogous, are characterized by the fact of their giving compounds of similar form RX*_n_*” (19). We, therefore, associate similarity with the possibility of substituting one element by another in an empirical formula. This approach is strictly based on substance composition and disregards other features, such as chemical stability and optical, electrical, and magnetic properties as well as vapor density, atomic volumes, and numerical relationships among atomic weights (20, 21), which were often considered by formulators of the SCE to gauge similarities among chemical elements.

**Fig. 3.**
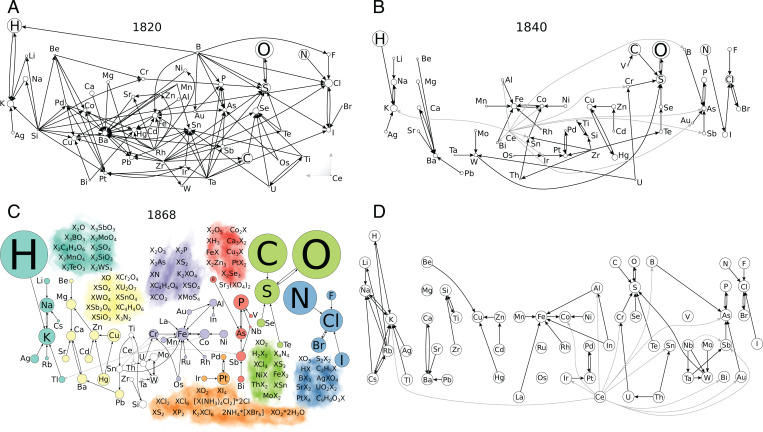
Evolution of the SCE. (*A*–*C*) SCEs of three different years. Arrows x→y indicate that *x* is most similar to *y*. Node (element) size is proportional to the number of substances composed by the element. Similarities of Ce are colored in light gray for the sake of readability. In *A*, all Ce similarities are collapsed for the sake of simplicity. Some of the formulae shared by elements with the same color (X) are shown in *C*. At https://mchem.bioinf.uni-leipzig.de/1868/main.html, readers can also select any set of elements to retrieve the formulae making similar the elements in any particular year. (*D*) The backbone of the SCE depicting the pairs of most similar chemical elements appearing in more than 60% of the SCEs between 1800 and 1869 (*Materials and Methods*).

As SCEs intend to show only the most remarkable similarities among elements, we display only maximum similarities for each element. This choice is justified because SCEs are customarily presented as tables in which similarities between neighboring elements are the largest. If this is the case, nonmaximal but important similarities can be recovered from sequences of maximum similarity relationships; for instance, Li being most similar to Na and Na to K mean that likely Li is quite similar to K as well (Fig. 3). Therefore, elements related by sequences of maximum similarities correspond to the notion of families (groups) of elements on periodic tables.

Having determined the key similarities among chemical elements, all that remains is to arrange them according to their atomic weights to retrieve the SCE of each year between 1800 and 1868. We depict these systems as similarity networks. Fig. 3*A*–*C* presents three of them. All 69 networks can be found at https://mchem.bioinf.uni-leipzig.de/1868/main.html, as well as the compounds contributing to each similarity.

**Fig. 4.**
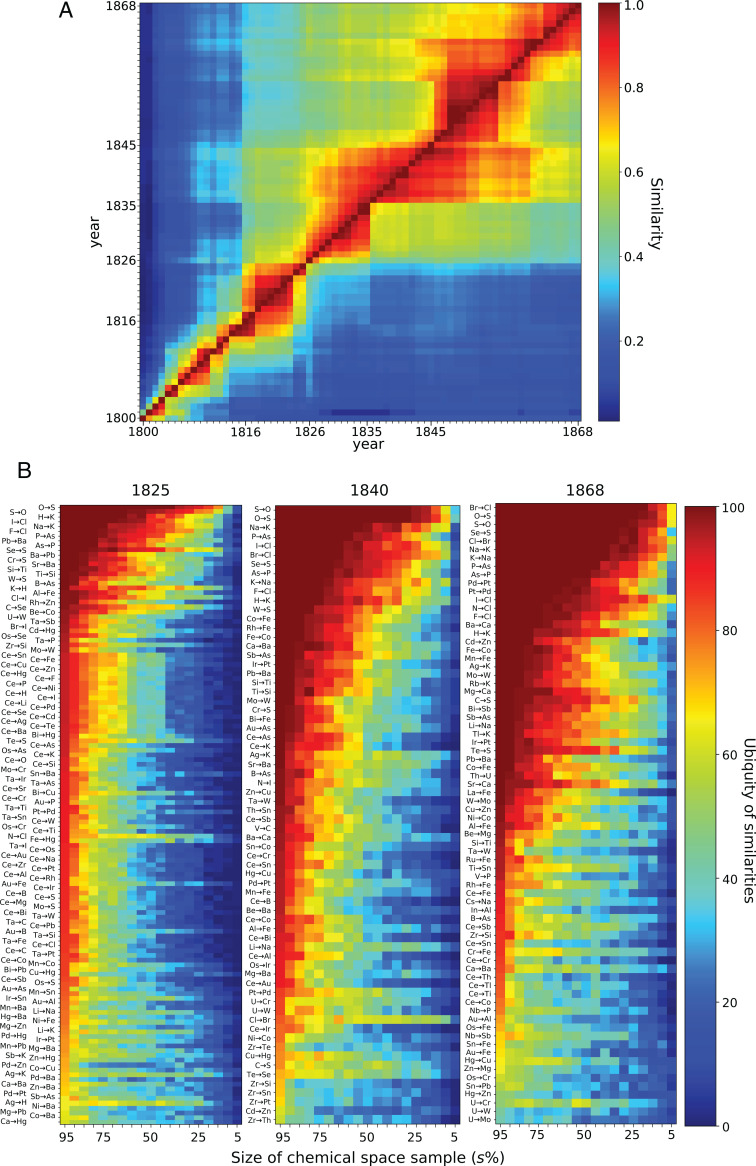
Similarity among SCEs and ubiquity of element resemblances. (*A*) Resemblance between SCEs. The heat map depicts similarity from the SCE of the column to the SCE of the row (*Materials and Methods*). Any row *y* indicates how similar the SCEs are, year after year, to the SCE of year *y*. Any column *x* shows which fraction of the SCE, year after year, is similar to the SCE of year *x*. (*B*) Ubiquity of the similarities of the SCEs of 1825, 1840, and 1868. The ubiquity of each similarity x→y corresponds to the percentage of appearance of such similarity in the sampled space of size *s*% (*Materials and Methods*). Plots for all years 1800 to 1868 are found in *SI Appendix*, Fig. S9.

Despite the increase in the number of elements (Fig. 1*F*), the number of “most similar” relationships decreased over time, dropping from a maximum of 166 in 1818 down to 69 in 1862 (*SI Appendix*, Fig. S6). To better assess this drop in similarity relationships, we calculated the similarity between SCEs of different years (Fig. 4*A* and *Materials and Methods*). The reddish region around the diagonal in Fig. 4*A* indicates continuity in the evolution of the SCE, as the most similar periodic system of any year is always one of an adjacent year. Nevertheless, it also shows qualitative shifts, the most visible of which appears in 1826, that suggest convergence to a stable SCE (https://mchem.bioinf.uni-leipzig.de/1868/main.html). The dark blue regions around the early years indicate that the SCEs of those years did not stand the test of time (https://mchem.bioinf.uni-leipzig.de/1868/main.html). Similarities in this early quarter of the century were mainly related to substitutions in chlorides, oxides, hydroxides, sulfates, and other typical inorganic compounds (https://mchem.bioinf.uni-leipzig.de/1868/main.html). Then, in 1826, there was a sharp stabilization of the SCE, as revealed by the light blue to yellow square in Fig. 4*A*, which indicates that more than 40% of the similarities found by 1826 remained in the SCE all the way to the end of the period.

The mechanisms behind this convergence were of substance discovery and tiebreaking. In the early years of the century, not enough compounds were known to determine the similarities that unveil the patterns of the SCE. For example, Br and Mo chemistries began, and new valencies for several elements were discovered: for instance, +1 for Ti, Cu, Zr, and Pd; +2 for Be; +3 for B, Fe, Co, and Ni; and +4 for Si. B went from having nine most similar elements to only one (As). Likewise, Si was not any more similar to 11 elements and became similar to only Ti (Fig. 3*A*) (https://mchem.bioinf.uni-leipzig.de/1868/main.html). These and other changes (https://mchem.bioinf.uni-leipzig.de/1868/main.html) reshaped the SCE and allowed for the appearance of families of similar elements that still were to be observed in the 1860s: for example, Fe, Co, Ni; B, P, As, Sb, and the halogens.

The reddish region between columns 1826 to 1860 and rows 1835 to 1845 (Fig. 4*A*) shows that about 80% of the similarities of the SCE observed between 1835 and 1845 were present since 1826 and lasted until 1860. During the 1860s, this resemblance dropped down to about 60%. In this 1835 to 1845 period, some elements reduced their number of most similar elements: for instance, Th and Cd, which went from having five most similar elements to having only one (https://mchem.bioinf.uni-leipzig.de/1868/main.html). In the first case, the change was caused by the appearance of +4 valency, a feature Th shared with Sn. Cd, in turn, reinforced its similarity to Zn through the discovery of common oxalates and other salts, including thiowolframates. The period after 1845 shows that the similarities observed after this year lasted but that there were also some transient similarities: for instance, those of Nb, Ta, Rb, and Cs (https://mchem.bioinf.uni-leipzig.de/1868/main.html).

The pattern observed in the period 1835 to 1845 suggests that by considering the chemical space only, a fairly accurate SCE could have been proposed as early as the 1840s. However, the problem of uncertainty on atomic weights still needs to be addressed, and we shall do it in the next section.

In order to detect the salient features of the convergence of the SCE, we determined the most frequent pairs of most similar elements, which we regard as the backbone of the periodic system from 1800 up to 1868 (Fig. 3*D*). This backbone structure shows families of elements, including alkali metals, halogens, chalcogens, pnictogens (without N), and {Fe, Co, Ni}, plus well-known families of transition metals, such as {Pd, Pt, Ir} and {Mo, W, Ta}.

Regarding the magnitude of similarities among chemical elements, that is their actual similarity values, *SI Appendix*, Fig. S8 shows that they were very weak. In fact, all over the period analyzed here, more than 80% of the similarities had values lower than 0.1: that is, less than 10% of the formulae of any element have been shared with its most similar element(s). The lowest similarity values ever recorded corresponded to those of organogenic elements (https://mchem.bioinf.uni-leipzig.de/1868/main.html). This fits Mendeleev’s concept of “typical elements” (12, 21), today called the singularity principle or the uniqueness of second period elements (22), which indicates that these elements possess weak similarities with elements of their families (23).

If the similarities were so small, how could they become so noticeable to chemists? We believe it has to do with ubiquity; these similarities extend over the whole spread of the chemical space, so that they are equally visible in any reasonably sized portion of the chemical space. To test this hypothesis, we took random samples of different sizes of the space, for every year, and analyzed how often the most similar relationships among elements were present in the samples (*Materials and Methods*). We found that most of the similarities observed in the first quarter of the nineteenth century required more than 50% of the chemical space to be detected, indicating that in this period, similarities of different elements were spread on different regions of the chemical space (Fig. 4*B* and *SI Appendix*, Fig. S9). As time went by, especially after 1830, similarities became more ubiquitous and easier to detect. This effect is particularly intense for similarities among elements involved in the organic expansion of the chemical space, such as organogenic ones, and metals, such as Na, K, Pd, Pt, Ba, and Ca (https://mchem.bioinf.uni-leipzig.de/1868/main.html). Similarities among those elements (for instance, S → O) detected as early as 1800 required at least 65% of the 1800 space to be observed, while by 1840, this fraction plummeted to 10% and dropped to 5% by 1868 (Fig. 4*B* and *SI Appendix*, Fig. S9). Likewise, Pd → Pt needed 80% of the space to be detected when first observed in 1822, a percentage that dropped to 55% by 1840 and to 15% by 1868. This ubiquity of Pd → Pt similarity was initially caused by inorganic substances as well as cyanide compounds and further strengthened by salts of organic acids (Fig. 4*B* and *SI Appendix*, Fig. S9). This contrasts with the larger amount of space required to detect similarities of elements that very seldom took part in the organic turn, such as Mo and W. Mo → W by the time of its appearance in 1825 required 70% of the space, by 1840 required 65%, and by 1868 required 30%, that is, six times more chemical space than S → O to be detected and twice the space to observe Pd → Pt (Fig. 4*B* and *SI Appendix*, Fig. S9).

### Meyer’s and Mendeleev’s Systems under the Presentist Approach.

The difficult detection of similarities among elements not taking part in the organic turn might explain why nineteenth century chemists, such as Meyer and Mendeleev, struggled with similarities among some transition metals (12, 21, 24–30) (*SI Appendix*, Fig. S7 and Table S2). Mendeleev also faced problems with the similarities of In and the rare earths he included in his system, especially because of the small number of compounds for these elements (*SI Appendix*, Table S2) (31). Remarkably, detecting In → Al by 1869, as Meyer did, required more than 75% of the chemical space (Fig. 4*D*). As Rocke (32) has pointed out, Meyer was able to pinpoint it by using the heuristic of his curve of atomic volumes. Examples of other similarities requiring large amounts of chemical space to be detected were Zn → Mg, Nb → P, and Nb → Sb. The first of these is explicit in Mendeleev and Meyer’s 1869/1870 systems (*SI Appendix*, Fig. S7), and the other two are explicit in Meyer’s system and discussed as similarities by Mendeleev (21) (*SI Appendix*, Fig. S7 and Table S2). Overall, we found that about 53% of the similarities among chemical elements arising from the chemical space were recovered by Meyer in his 1864 and 1868 systems (true positives) (*SI Appendix*, Table S3). Almost a quarter of nonsimilarities of the 1864 chemical space were observed as similarities by Meyer (false positives) (*SI Appendix*, Table S3). This fraction plummeted in 1868 to about 7%. At any rate, the best agreement between Meyer’s systems and the system allowed by the chemical space was achieved in 1869/1870, when 62% of the similarities of the space were gauged by his system, while there were only 6% nonsimilarities observed as similarities. Mendeleev, in turn, attained 58 and 10% of true and false positives, respectively (*SI Appendix*, Table S3). Note that the (dis-)agreements here discussed are based on the similarities reported by the two chemists in their systems, which were abundant and detailed in Mendeleev’s case and very seldom discussed by Meyer, in which case similarities needed to be interpreted from his periodic tables. Also, the greater detail of Mendeleev’s discussions on similarity is expected to yield a higher rate of false positives due to our methodology being based on maximum similarities.

### Retrodictive Approach to the Evolution of the SCE.

The presentist approach takes for granted a stable set of atomic weights and of empirical and molecular formulae corresponding to current standards. Nevertheless, the expansion of the chemical space and its influence upon the SCE require considering the development of the atomic theory. That is, it requires considering the various nineteenth century competing sets of atomic weights associated with different theoretical and experimental settings (4, 33), which led to chaos of formulae before the 1860s (34). Hence, different atomic weights produce different orderings of the elements and different formulae, so that different chemists working with different sets of atomic weights could find widely different similarities among chemical elements: that is, different SCEs even if they worked with the same experimental data. Here, we analyze the possible SCEs resulting from different perspectives of the nineteenth century chemical space spawned by several distinct sets of atomic weights proposed over the period.

**Fig. 5.**
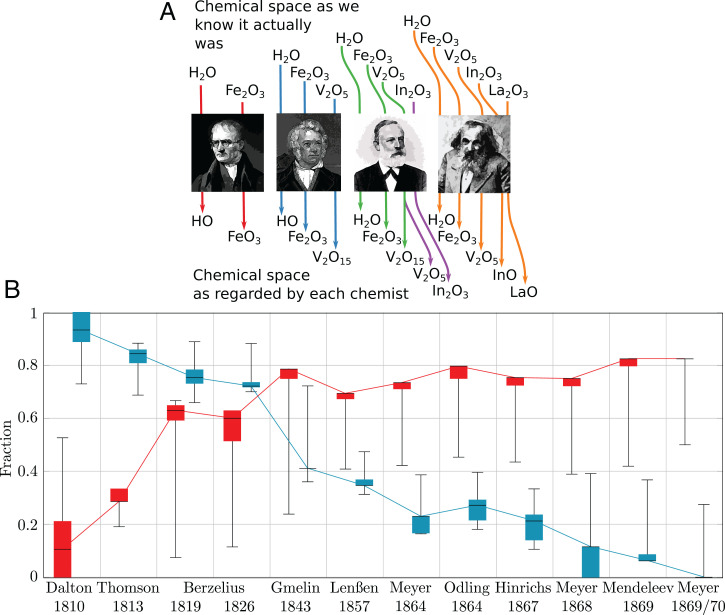
Contrast between SCEs calculated with nineteenth century and with modern atomic weights. (*A*) Examples of modified formulae according to the atomic weights of (from left to right) Dalton (1810), Gmelin (1843), Meyer (1864, green; 1869/1870, purple), and Mendeleev (1869). For every chemist publishing a set of atomic weights in year *y*, known Reaxys substances ($S_{y-1}$) up to year *y* – 1 (inclusive) were retrieved, and the corresponding SCE $P_{y-1}$ was obtained. Afterward, formulae of substances $S_{y-1}$ were transformed to fit a chemist’s atomic weights within 20 different tolerance values (*τ*), each *τ* yielding an SCE with similarities gathered in $P_{y-1}^{\tau }$ (*Materials and Methods* and *SI Appendix*). (*B*) Red (true positive) indicates the efficacy of a chemist’s atomic weights in approaching *P*_1868_, measured as $\left|P_{y-1}^{\tau }\cap P_{1868}|/|P_{y-1}\cap P_{1868}\right|$; a value of one means that they are just as effective as our modern atomic weights. Blue (false positive) indicates the fraction of transient similarities in $P_{y-1}^{\tau }$: that is, similarities not observed by 1868. These were calculated as $\left|P_{y}^{\tau }\setminus P_{1868}|/|P_{y}^{\tau }\right|$; a small value means that most of a chemist’s SCEs obtained from his atomic weights were observed in 1868. Box plots depict medians (black horizontal lines) and minimum/maximum values as whiskers.

In the nineteenth century, empirical data on composition came in the form of mass percentages for each element. For instance, Dalton knew that water was made of 88 and 12% by weight of oxygen and hydrogen, respectively. From Dalton on, chemists assumed formulae for key compounds, such as water, ammonia, and oxides. Thus, chemists selected an element and assigned a reference atomic weight to it, and they recorded atomic weights of other elements relative to that one. The initial assumptions thus propagate through all the calculations, therefore creating a different chemical space for each chemist (Fig. 5*A*). For example, Dalton’s reference was an atomic weight of one for hydrogen. He assumed HO as the formula of water, therefore yielding an atomic weight of seven for oxygen. This led to molecular formulae of oxides whose coefficients are around half of those we know today. The determinations were made even more difficult by the varying quality of the experimental data (4, 33).

We gathered 13 sets of atomic weights (*SI Appendix*, Table S4) corresponding to data published by Dalton [1810 (35)], Thomson [1813 (36)], Berzelius [1819 (37) and 1826 (38–40)], Gmelin [1843 (24)], Lenßen [1857 (25)], Meyer [1864 (26), 1868 (41), and 1869/1870 (28)], Odling [1864 (29)], Hinrichs [1867 (30)], and Mendeleev [1869 (12)] plus the currently accepted atomic weights. Starting with Gmelin, these sets of atomic weights were proposed by authors who actually devised SCEs (7). Although Dalton, Thomson, and Berzelius did not aim at devising SCEs, they were some of the key figures in the development of the atomic theory (4, 33), which is why we also explored the effects of their atomic weights upon the SCEs that could have been obtained from their respective chemical spaces. *SI Appendix*, Fig. S10 shows the elements comprised by each system of atomic weights, which range from 30 for Dalton to 60 for Mendeleev. Information on the selection of these elements is found in *SI Appendix*, Table S5.

As any SCE is based on ordering and similarity of its chemical elements (1), we analyzed the different orderings of elements associated with each set of weights. In all cases, they agreed in more than 80%, even with the current atomic weights (*SI Appendix*, Table S6). This indicates that the ordering relationships among elements were rather stable since the beginning of the nineteenth century. To determine element similarities, it is necessary to reconstruct the formulae spanned by each system of atomic weights (Fig. 5*A*). As there is no systematic record of the chemical formulae corresponding to the assumptions of each chemist, we devised an algorithm to obtain approximate formulae meeting the assumptions of the chemists here analyzed (*Materials and Methods*). This entails, for instance, approximating the current Fe_2_O_3_ to FeO_3_ according to Dalton (Fig. 5*A*). Our procedure takes all Reaxys formulae known by the time of publication of each chemist’s atomic weights and rescales the modern formulae to fit chemist’s atomic weights within 20 different levels of tolerance (*Materials and Methods* and *SI Appendix*). Often, the higher the tolerance, the lower the perturbation of Reaxys formulae.

For each chemist’s set of atomic weights and level of tolerance, we obtained a corresponding chemical space, which led to an associated SCE holding a set of similarities among chemical elements. In order to quantify how close a chemist’s set of atomic weights was to gauging the similarities allowed by the actual chemical space of the chemist’s time (calculated with our contemporary atomic weights), we computed the fraction depicted in the red plot of Fig. 5*B* (*SI Appendix*, Fig. S11). This corresponds to the true-positive rate, indicating to which extent the old atomic weights sharpened our ancestors’ capabilities of discovering the SCE of 1868. As a chemist’s space could lead to several transient similarities not remaining until 1868, we also quantified a chemist’s fraction of transient similarities (Fig. 5*B*, blue and *SI Appendix*, Fig. S12). They correspond to the false-positive rate (Fig. 5*B*, blue).

By inspecting Fig. 5*B*, we observe how, as the century progressed, SCEs resulting from fitting chemists’ sets of atomic weights contain more and more 1868 similarities and how transient similarities were reduced. There is a remarkable leap with Gmelin, who becomes a turning point in the trends, separating SCEs with many transient similarities and few standing the test of time (on Gmelin’s left in Fig. 5*B*) from SCEs rich in 1868 similarities and with very few transient similarities (on Gmelin’s right in Fig. 5*B*). Gmelin’s atomic weights led to SCEs containing about 78% of the 1868 similarities and about 40% of transient similarities. This is an improvement when contrasted with the SCEs obtained with the atomic weights of Gmelin’s predecessors. For instance, the SCEs obtained with Dalton’s weights contain about 10% of 1868 similarities and 93% of transient ones. For those of Berzelius (1826), the percentages were 60 and 73%, respectively. The lack of accuracy of pre-Gmelin SCEs is caused by the many changes the chemical space underwent. Nevertheless, in the years before Gmelin, Berzelius’ 1819 weights stand out. Despite their 75% of transient similarities, Berzelius’ atomic weights led to SCEs with 63% of 1868 similarities.

The remarkable separation of the two plots after Gmelin (Fig. 5*B*) shows the strong relationship between the theoretical and experimental advances the atomic theory brought about and the raise of the backbone of the periodic system. Interestingly, this is particularly evident in the 1840s, which agrees with the results of our presentist approach and motivates the question on the factors delaying the formulation of the SCE about a quarter of a century.

### Meyer’s and Mendeleev’s Systems under the Retrodictive Approach.

By analyzing the SCEs obtained from Meyer’s and Mendeleev’s atomic weights, we found that each new version of Meyer’s weights achieves more 1868 similarities and reduces the amount of transient similarities. His last set of atomic weights led to SCEs with no transient similarities matching 82% of the 1868 similarities. In turn, Mendeleev’s atomic weights produce SCEs with 83% of 1868 similarities and 6% of transient similarities. These improvements were mainly caused by accurate determinations of atomic weights of elements, such as V. Meyer’s atomic weight for V (137) came from Berzelius, who had determined it in 1831 making two mistakes: regarding a V oxide as the metal itself and considering V as hexavalent (42). By 1868, these errors were corrected by Roscoe (43), who updated the atomic weight of V to 51.3, which was taken by Meyer (28) as 51.2 and by Mendeleev (19) as 51 (*SI Appendix*, Table S4). Similarly, Ta atomic weight passed from 137.6 in 1864 for Meyer to 182 for Mendeleev and to 182.2 for Meyer by 1869 (*SI Appendix*, Table S4). Mendeleev also faced problems with rare earths and In. The atomic weights of Ce, La, and In were two-thirds their current figures, and those of U and Th were half their current values (*SI Appendix*, Table S4). These problems were mainly caused by the small number of compounds of those elements: for instance, only five for La by 1868 (https://mchem.bioinf.uni-leipzig.de/1868/main.html). These results coincide with the different stances the two chemists had regarding the SCE. Meyer favored accurate atomic weights and experimental information, and Mendeleev favored completeness (11, 32), as noted in the several elements left aside by Meyer that were included by Mendeleev.

## Conclusion

The expansion of the chemical space led the SCE to converge to a stable structure of similarity and order relationships, eventually unveiled in the 1860s. Convergence to this backbone structure was marked by two transitions, one around 1826 and another one in the decade from 1835 to 1845. In the first quarter of the nineteenth century, the rapid discovery of elements and their compounds led to highly dissimilar SCEs not standing the test of time. This changed in 1826 when the discovery of elements slowed down, allowing chemists to further explore the chemistry of the known substances and to discover compounds that revealed new valencies (therefore, new similarities among chemical elements). Several of these similarities remained until the 1860s, providing a rather stable SCE. A further stabilization of the SCE occurred between 1835 and 1845, where the SCE further converged to its backbone structure. This stabilization was driven by the strengthening of some similarities and by the discarding of others not supported any more by the chemical space at those times. This “cleaning” period of the SCEs was followed by the further discovery of elements and their compounds, which slightly perturbed the SCE and that finally led it to its stable form unveiled in the 1860s.

Despite the convergence of the SCE to its backbone structure as driven by the chemical space, the detection of such a structure was hindered by the biased expansion of the space. The rise of organic chemistry in the 1830s facilitated the recognition of similarities among strongly represented elements in the chemical space, such as O, H, C, N, and S, and among metals often associated with organic compounds, such as Na, K, Pd, Pt, Ba, and Ca. In contrast, similarities among metals poorly represented in the organic turn were difficult to detect, which might have contributed to their difficult arrangement on the SCE by several of its formulators.

By analyzing the structure of the SCE across time, we found that it was mainly determined by the similarities among chemical elements rather than by their ordering, provided by atomic weights. That is, gauging the similarities was the “hard part,” as the matter of order was pretty much settled since the dawn of the nineteenth century. Nineteenth century atomic weights actually led to quite similar orderings of the elements, and several of these sets of weights, starting with those proposed by Berzelius in 1819, allowed for devising chemical spaces encoding several similarities standing the test of time. This evidences the remarkable abilities of Berzelius, who by 1819, witnessed a chaotic chemical space and nonetheless, was able to detect some similarities of the 1860s. The subsequent interplay of the expanding space and of atomic debates led to more refined sets of atomic weights, as the one introduced by Gmelin in 1843. Gmelin’s weights not only gauged more features of the SCE of the 1860s but also, reduced to a large extent similarities not well supported by the chemical space. This indicates that sets of atomic weights as early as the 1840s already allowed for the recognition of the salient features of the SCE of the 1860s, despite the central role ascribed by Meyer, Mendeleev, and others to Cannizzaros’ atomic weights adopted in the 1860 Karlsruhe conference (4, 7).

Although Gmelin (24) illustrated some features of the structure of the SCE in his V-shaped SCE (7) of 1843 (ref. 24, p. 457), the mature chemical space of the 1840s did not trigger the formulation of the SCE at that time. The question that arises is about the further factors accompanying the chemical space, which delayed the formulation of the SCE around a quarter of a century. We believe these factors include social and epistemic aspects of the unfolding of chemistry.

Regarding Meyer and Mendeleev, both chemists enjoyed a mature chemical space and a rather stable set of atomic weights, which contributed to the formulation of their SCEs. Although their systems coincide to a large extent with the possible SCEs of their times, the underlying reasons they had to finally arrange elements as they did cannot be reduced solely to the chemical space. For instance, both chemists regarded Cu and Ag as very similar (*SI Appendix*, Fig. S7), which does not agree with the chemical space of their time. Mendeleev believed in Tl and Cs similarity, a thought Meyer shared until 1869/1870, when he arranged a new family containing Tl (current group 3). Yet, the SCEs resulting from Meyer’s and Mendeleev’s weights show that Tl is most similar to K. Likewise, by 1869, the similarity between Pd and Pt was detectable with only 15% of the chemical space; however, Mendeleev did not include it in his system and did not comment upon it, while Meyer’s system included it. Further aspects used to arrange elements on the system included substance physical properties (19, 28) and numerical relationships among atomic weights (42). Which heuristics did they use to sometimes rely more on arithmetic than on chemical or physical resemblance? These as well as the quarter of a century delay in the formulation of the SCE are questions and hypotheses motivating common work among chemists, historians, mathematicians, and computer scientists. A full understanding of the driving forces leading to the formulation of the SCE in the 1860s may conduct to the computational reconstruction of its formulation as it has been achieved, for instance, for the discovery of the urea cycle (44).

From a methodological stance, we introduced an algorithm that transforms current chemical formulae to fit any given system of atomic weights. In conjunction with chemical databases, it allows for computing approximations to the chemical space known to past chemists. It could be applied to other chemists involved in either devising sets of atomic weights or SCEs, which may include, besides those here studied, Wollaston, Döbereiner, Pettenkofer, Kremers, Gladstone, Dumas, Newlands, Williamson, and Béguyer der Chancourtois (4, 7). We believe there is untapped potential in this approach. Our algorithm is freely available at https://keeper.mpdl.mpg.de/d/2284ca87fd124ea9823f/. Likewise, the interactive information available at https://mchem.bioinf.uni-leipzig.de/1868/main.html allows for the exploration of the chemical space and its effects upon the SCE in a user-friendly manner. There, users can retrieve details of our claims and may further pose and solve new questions by interacting with the data supporting this research.

The method here presented to obtain a SCE given a chemical space is not only restricted to the past. It can actually be used to study possible futures of the SCE, for instance, by exploring the SCEs generated based on chemical spaces produced under extreme conditions of pressure and temperature (3) or computationally generated by the iterative application of types of reactions upon sets of chemicals (45).

As the chemical space has exponentially grown since 1800 up to date and it keeps being concentrated on organic chemistry (5), the question that arises is whether the current SCE is akin to the one formulated in the 1860s or whether twentieth and twenty-first century chemistry has changed its shape. If the SCE provides a big picture of chemistry, data-driven studies as the one here presented may become a tool to convey real-time big pictures of chemistry with natural implications for the teaching and the future of the discipline.

We hope our results and methods contribute to the ongoing development of computational approaches to the history of science and the evolution of knowledge (8, 46, 47).

## Materials and Methods

### Data.

We retrieved 21,521 single-step reactions with publication year before 1869 from Reaxys. These reactions had 11,451 associated substances with their respective formulae. Some of them were curated, and others were discarded (*SI Appendix*), leading to 11,356 substances. We associated each of these substances with its earliest publication year (in a chemical reaction) and with its molecular formula. All data and code used for this research are available (see *Data Availability* below).

### Theoretical Combinations.

For *n* known elements in a given year, its theoretical number of combinations of size *s* is $\mathrm{theo}(n,s)=\left(\begin{array}{c} n\\ s \end{array}\right)$. Hence, the theoretical number of combinations of *n* elements is $\mathrm{theo}(n)=\sum_{s=2}^{n}\left(\begin{array}{c} n\\ s \end{array}\right)$. This is a rough upper bound disregarding valency and compound stability. The percentage of theoretical combinations actually observed (Fig. 1*F*) corresponds to exp(n,s)/theo(n,s), where exp(n,s) is the number of reported substances with *n* elements whose combinations size is *s*.

### Backbone of the SCE.

Fig. 3*D* depicts similarities i→j appearing in more than 60% of the SCEs containing *i* and *j*. This percentage is computed as (f(i→j)/1868−y)×100, where f(i→j) is the number of SCEs containing i→j and *y* is the first year in which *i* and *j* appear in an SCE. The normalization factor 1868−y represents the time window where the similarity i→j could have been observed.

### Similarity between SCEs.

An SCE is devised as described in Fig. 2 and stored as a collection *N* of pairs of elements $\left(e_{i},e_{j}\right)$, indicating the similarity of element *e_i_* with respect to element *e_j_* ($e_{i}\to e_{j}$ in Fig. 3). Each year *x* has an associated network *N_x_*. We quantify the relative fraction of similarities of *N_x_* observed in another network *N_y_* as $s(x,y)=|N_{x}\cap N_{y}|/|N_{x}|$, where $\left|N_{x}\right|$ indicates the number of pairs (*e_i_*, *e_j_*) in *N_x_*. Whenever a network *N_y_* is calculated from a chemical space approximated with a tolerance *τ* (the retrodictive approach), the similarity of such a network regarding the corresponding network to 1868 is given by $s_{\tau }(y,1868)=|N_{y}^{\tau }\cap N_{1868}|/|N_{1868}|$.

### Sampling the Chemical Space.

For each year, we randomly took s% of the space and determined the most similar element(s) for each element. This experiment was carried out 100 times. For each similarity x→y resulting for the whole space of that year, we counted in how many of the 100 experiments x→y appeared. The higher this number, the more stable the similarity is. We carried out this analysis for 19 sample sizes (95, 90, 85, …, 5%). The higher these numbers for different values of s%, the higher the ubiquity of x→y in the chemical space (Fig. 4*B*).

### Chemical Spaces from Atomic Weights.

As contemporary atomic weights are related by simple fractions with atomic weights of different chemists (*SI Appendix*, Table S4), we adjusted a contemporary chemical formula:

[1] $$F={\text{X}}_{x}{\text{Y}}_{x}\ldots {\text{Z}}_{z}\;\text{to}\;F_{A}={\text{X}}_{xf_{A(X)}}{\text{Y}}_{yf_{A(Y)}}\ldots {\text{Z}}_{zf_{A(Z)}}.$$

Here, X, Y, …, Z are chemical elements, and x,y,…,z are their stoichiometric coefficients in *F*; $f_{A(X)},f_{A(Y)},\ldots ,f_{A(Z)}$ are the respective coefficients modifying x,y,…,z to yield the formula *F_A_* as an approximation to that regarded by chemist *A*. Coefficients *f_A_* are calculated as follows: knowing the current [*W*(*e*)] and chemist’s [*A*(*e*)] atomic weights of element *e* (*SI Appendix*, Table S4) as well as the respective values for hydrogen [*W*(*H*) and *A*(*H*)], we calculate the ratios (W/A)(e)=(W(e)/W(H))/(A(e)/A(H)) and (A/W)(e)=(A(e)/A(H))/(W(e)/W(H)). Our aim is determining the simplest fraction *f* approximating either (W/A)(e) or (A/W)(e). As these ratios either fall in the real interval (0,1] or correspond to figures of the form α+β, where *α* is an integer and *β* is a real number in the interval (0,1], we need to find a 0<f≤1 that best approximates either *β* or the ratio falling in the interval (0,1]. The best *f* corresponds to a fraction of a Farey sequence (48) (*SI Appendix*) minimizing the relative error of the approximation (error(r,f)=|r−f|/r, with *r* either (W/A)(e) or (A/W)(e)). We allowed 20 different error tolerances *τ* for the approximation, from 1 to 20% of relative error, in such a manner that for each *τ*, the selected fraction *f* always approximates *r* with an error≤τ. Hence, for a given *τ*, a fraction *f* is found, which corresponds to the coefficient $f_{A(e)}$ in Eq. **1**. By applying this algorithm to each element of the contemporary formula *F*, the respective fractions are found, and the adjusted formula *F_A_* of chemist *A* is found (further details are in *SI Appendix*). By applying this method, it is found, for instance, that contemporary Fe_2_O_3_ corresponds to FeO_3_ according to Berzelius’ table of atomic weights of 1819 (*SI Appendix*, Table S4).

## Supplementary Material

Supplementary File

## Data Availability

Data (molecular formulae) and code have been deposited in Keeper (https://keeper.mpdl.mpg.de/d/2284ca87fd124ea9823f/) (49). All other study data are included in the article and/or *SI Appendix*.

## Footnotes

^*^Reaxys is a trademark of Elsevier Limited (copyright © 2022 Elsevier Limited except certain content provided by third parties). Reaxys data were made accessible to our research project via the Elsevier R&D Collaboration Network.
