# Supplementary material for: The expansion of chemical space in 1826 and in the 1840s prompted the convergence to the periodic system
Source: Proc Natl Acad Sci U S A. 2022 Jul 22;119(30):e2119083119. doi: 10.1073/pnas.2119083119 (PMC9335271; doi:10.1073/pnas.2119083119)
Supplement: Supplementary File [file pnas.2119083119.sapp.pdf]

Supplementary Information to

The expansion of chemical space in 1826 and in the 1840s prompted the convergence to the periodic system

Wilmer Leal, Eugenio J. Llanos, Andrés Bernal, Peter F. Stadler, Jürgen Jost & Guillermo Restrepo

Contents

1 Data curation **2**

2 Disregarded elements and their separations **2**

3 The expanding chemical space **4**

4 Evolution of some molecular fragments **10**

5 Quantifying similarity among chemical elements **12**

6 Similarity values among chemical elements **13**

7 Periodic systems by Meyer and Mendeleev up to 1869/70 **14**

8 Similarities as discussed by Mendeleev (1869) **15**

9 Distribution of similarity values among chemical elements **16**

10 Perturbing the chemical space and its effect on the system of chemical elements **18**

11 Contrasting Meyer and Mendeleev' systems of chemical elements with those of the chemical space (presentist approach) 36

12 Evolution of the system of chemical elements (retrodictive approach) 39

13 Approximating formulae according to the set of atomic weights of a given chemist 45

14 Similarities in the SCE of 1868 and their relationships with those of each chemist's SCE 49

1 Data curation

We retrieved 21,521 single-step reactions with publication year before 1869 from Reaxys, accounting for 11,451 substances. By eliminating substances with unreliable formulae, e.g. holding intervals as stoichiometric coefficients, such as $Ta_{1.15-1.35}S_2$ and by manually curating 245 formulae with non-integer amounts of crystallisation species, e.g. $CdCO_3$*$0.5H_2O$, curated as $CCdHO_{3.5}$, we ended up with 11,356 substances. We associated each of these substances with its earliest publication year (in a chemical reaction) and with its molecular formula.

2 Disregarded elements and their separations

Er and Yt, along with In, were elements whose identity was questioned by Mendeleev and expressed as ?Er, ?Yt and ?In in his table (1). Yt was the symbol used until 1920 for Y (2) and the first Y (or Yt) reaction is from 1872. Thus, neither Mendeleev nor Meyer had clear information about the element. Er was also problematic. By 1868 it was unknown that Er was actually a mixture of an element later (1878) coined Er and Yb. Er was separated one year later into Ho and the current Er and Tm. The same year, Yb was found to be accompanied by current Sc. In 1886 Ho was separated into the current Ho and Dy. The 1879 Yb was found to be a mixture of current Lu and Yb in 1907 (3). It is now known that Di, reported by Mendeleev as an element, was found as a mixture of Di and Sm in 1879. One year later, Sm was separated into Sm and the current Gd; this Sm was found by 1901 to be made of current

Eu and Sm. The 1879 Di turned out to be a mixture of current Pr and Nd in 1885. Therefore, we excluded Er, Yt and Di from our analysis and all the study is based on our findings for the 60 elements shown in Figure 1a (main text).

On the reasons to disregard H, La, Ce, Th and U, Meyer wrote: “The table contains, sorted by increasing atomic weights, with the sole exception of hydrogen, which seems to claim an exceptional position, all elements, the atomic weights of which have so far been determined from the gas density of their compounds or from their thermal capacity, and also Be and In with atomic weights presumably derived from the equivalent weight, a total of 56 elements.” He originally wrote “*Die Tabelle enthält, nach steigenden Atomgewichten geordnet, mit alleiniger Ausnahme des Wasserstoffs, der eine Ausnahmestellung zu beanspruchen scheint, alle Elemente, deren Atomgewichte aus der Gasdichte ihrer Verbindungen oder aus ihrer Wärmecapacität bis jetzt bestimmt worden, und ausserdem Be und In mit vermuthungsweise aus dem Aequivalentgewicht abgeleiteten Atomgewichten, im ganzen 56 Elemente*” (4).

3 The expanding chemical space

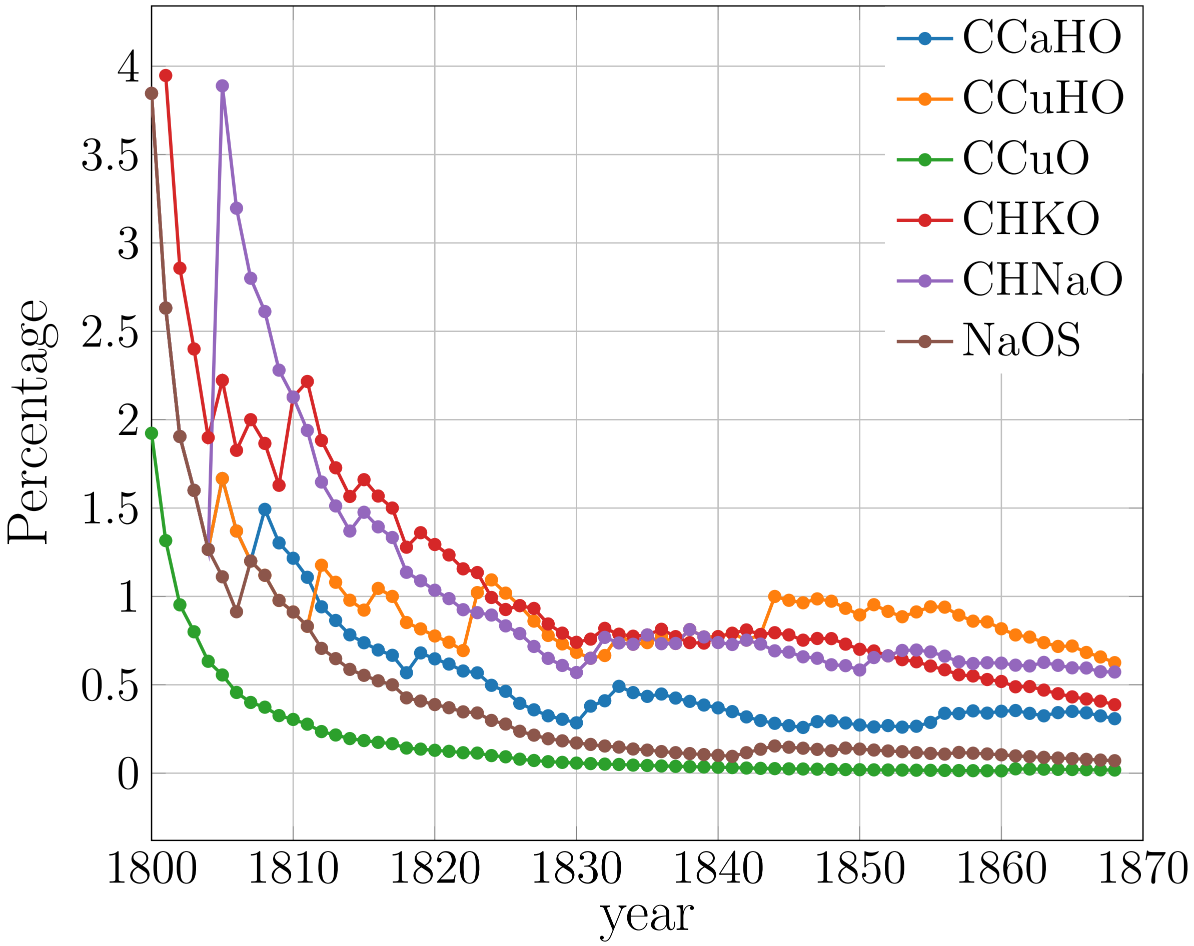

Figure S1: Percentage of chemical space spanned by some combinations containing metals.

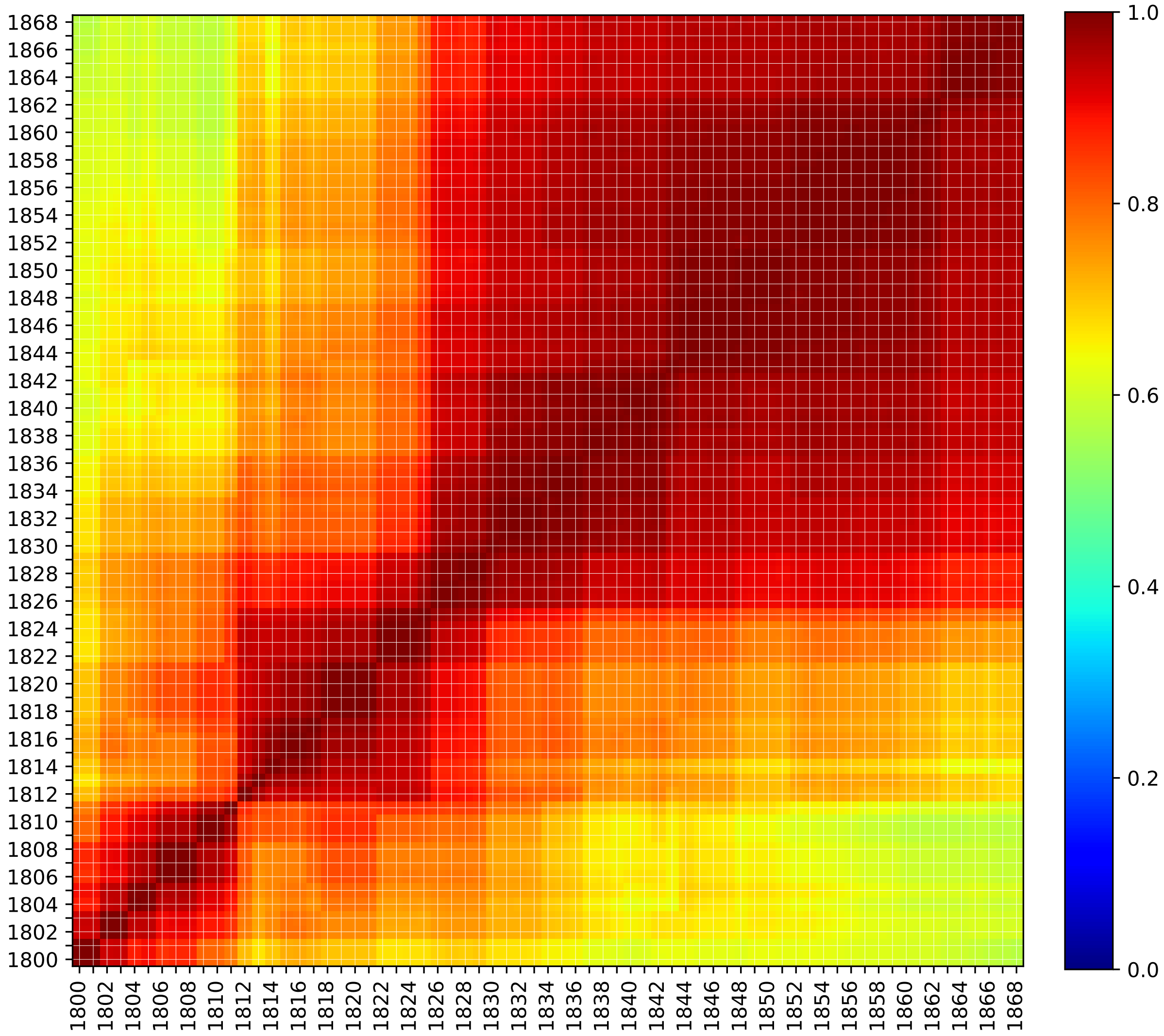

Figure S2: Order correlations of combinations. To determine to which extent the combinations of one year are populated differently or similarly from the combinations of any other year, we calculated the Spearman rank correlation. A given year has a list of combinations with the corresponding number of substances each of the combinations represents up to that particular year. For each year, combinations were ordered according to their number of substances. The Spearman rank correlation was calculated among those ordered lists of combinations. Here the resulting correlation matrix is shown as a heatmap.

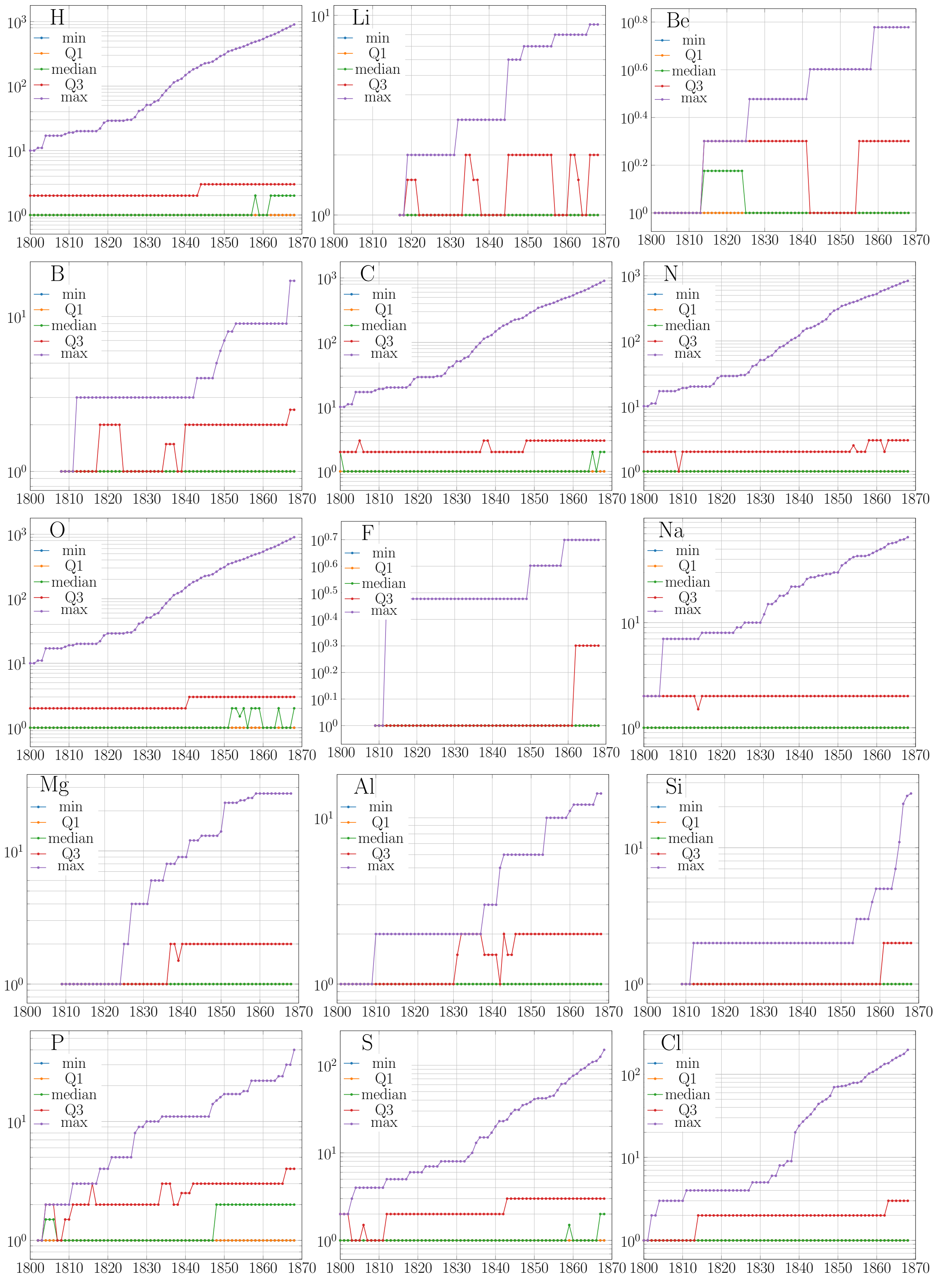
H
Li
Be
B
C
N
O
F
Na
Mg
Al
Si
P
S
Cl
min
Q1
median
Q3
max
1800
1810
1820
1830
1840
1850
1860
1870

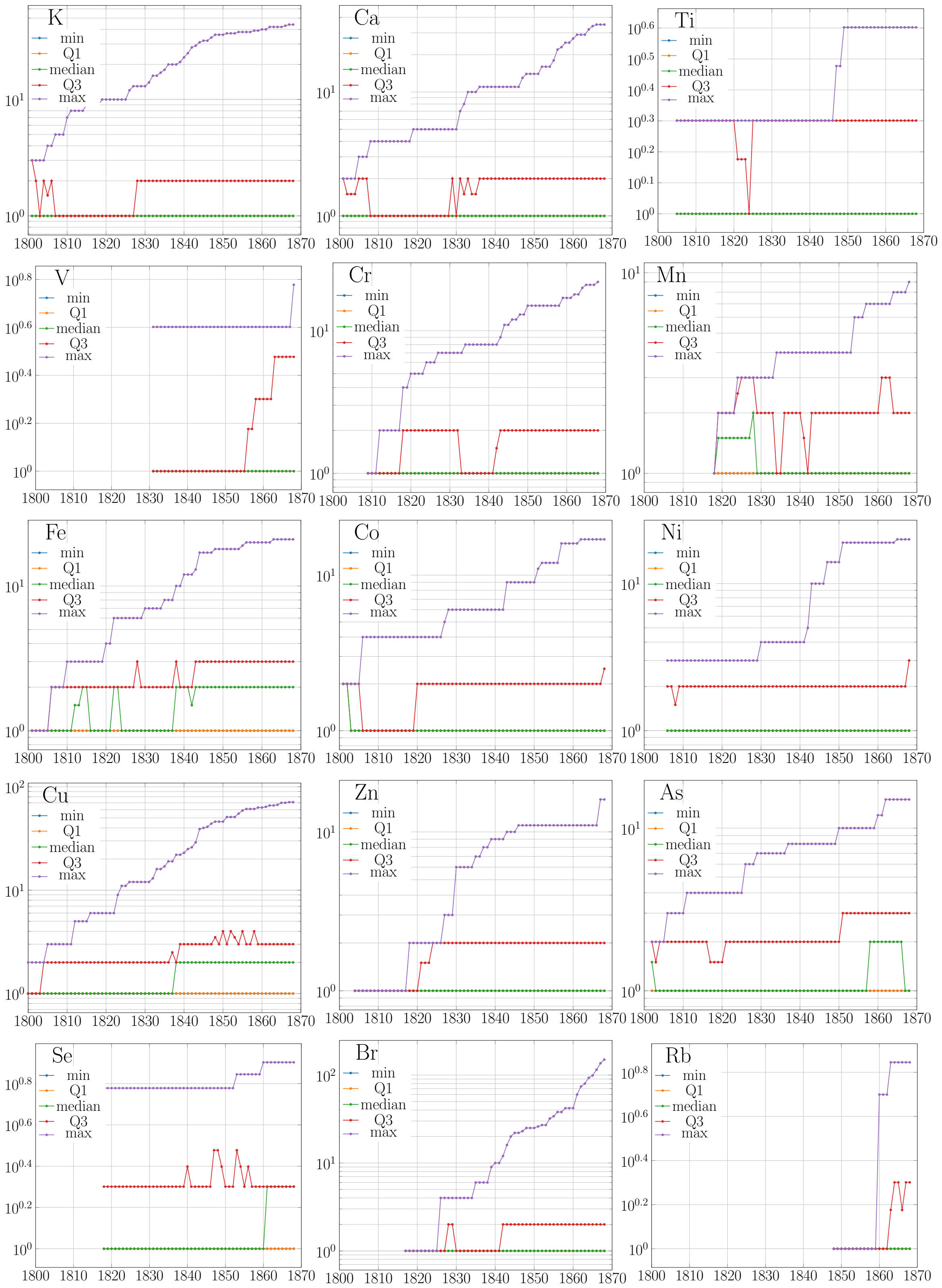
K
Ca
Ti
V
Cr
Mn
Fe
Co
Ni
Cu
Zn
As
Se
Br
Rb
min
Q1
median
Q3
max
1800
1810
1820
1830
1840
1850
1860
1870

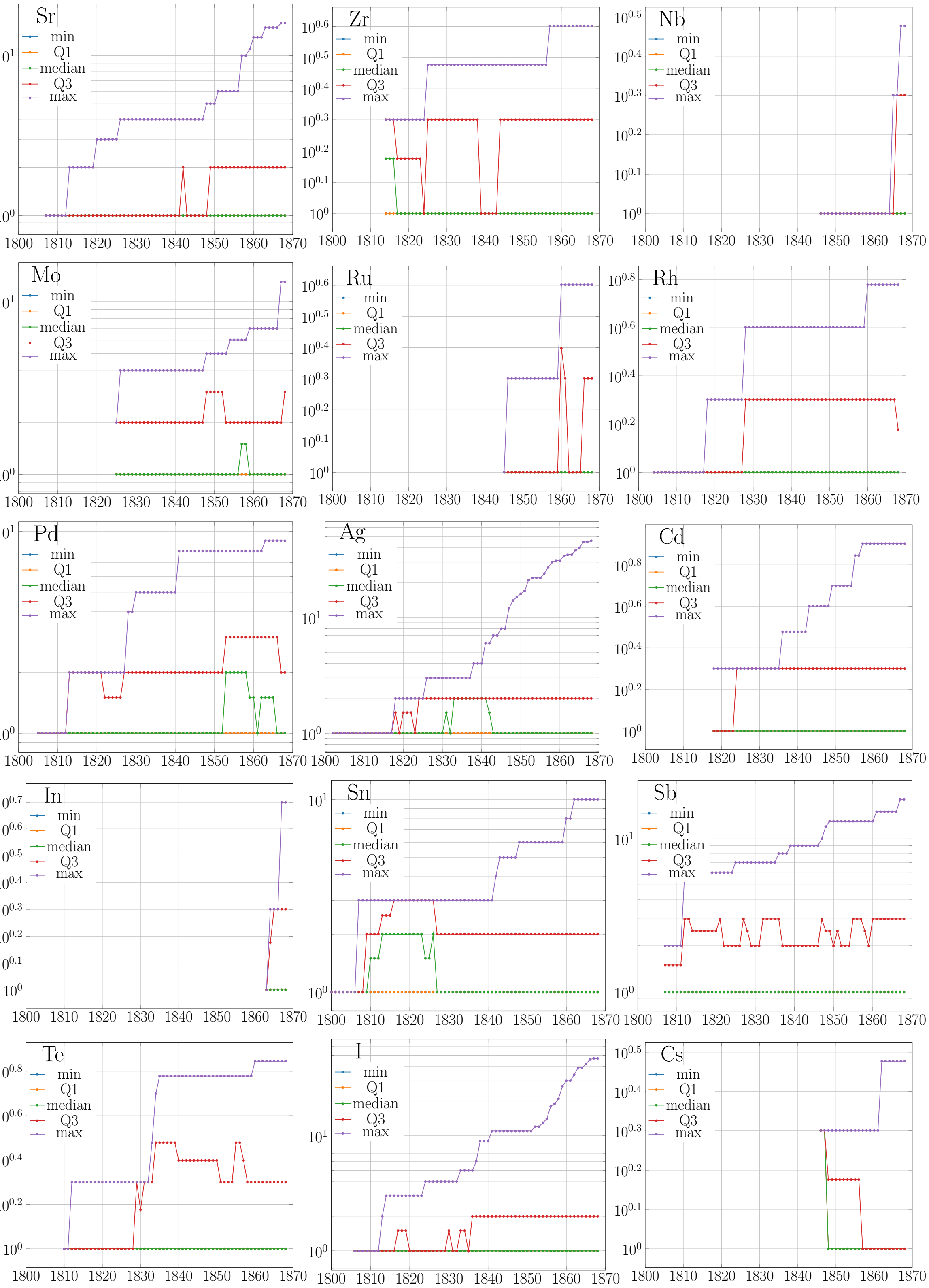
Sr
Zr
Nb
Mo
Ru
Rh
Pd
Ag
Cd
In
Sn
Sb
Te
I
Cs
min
Q1
median
Q3
max

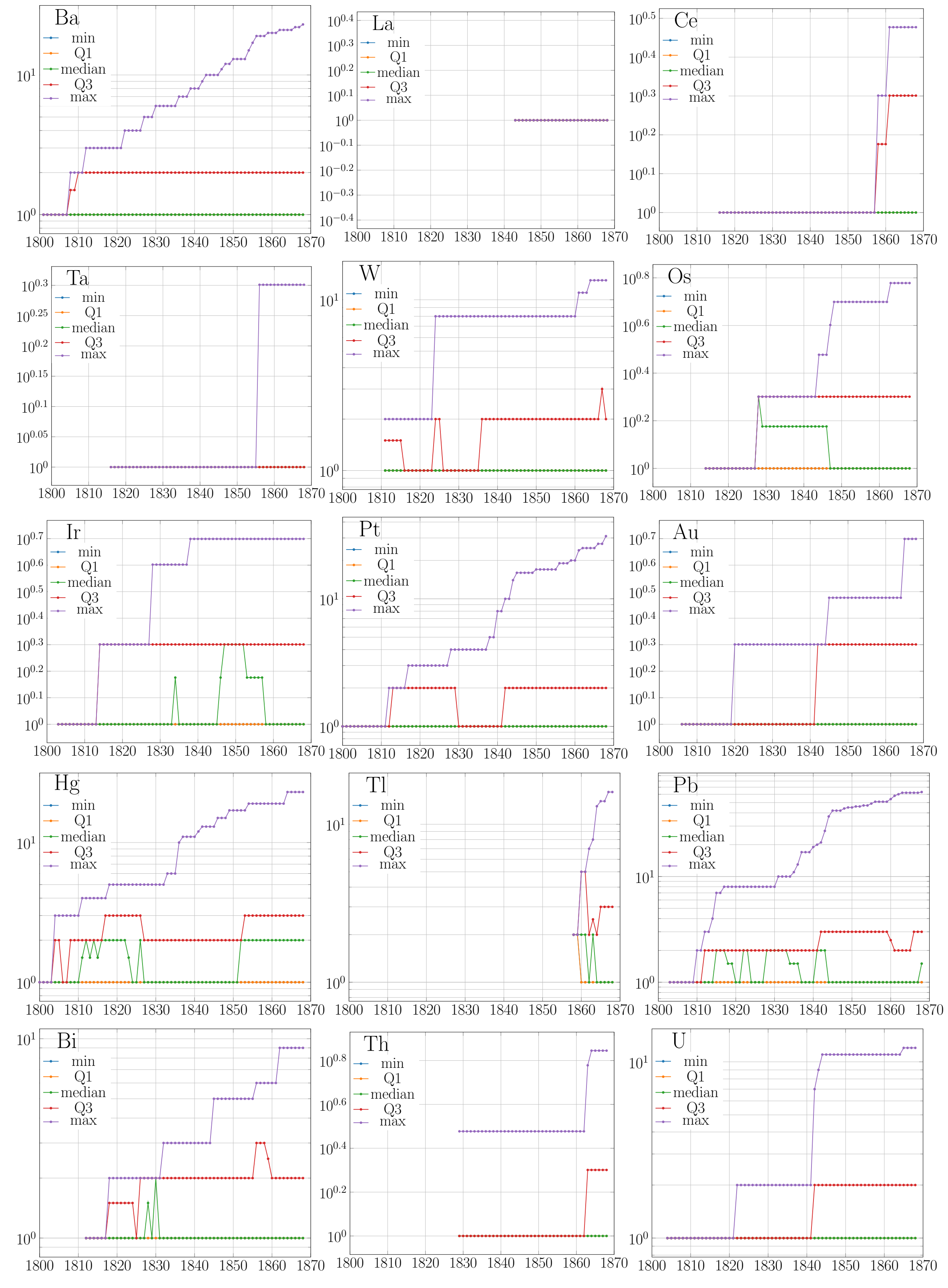

Figure S3: Distribution of substances over combinations for each chemical element.

Table S1: Most populated combinations. Combinations including metals in red and those highlighted in gray include metals and carbon.

1800	1805	1810	1815	1820	1825	1830	1835	1840	1845	1850	1855	1860	1865	1868
CHNO	CHNO	CHNO	CHNO	CHNO	CHNO	CHNO	CHO	CHO	CHO	CHNO	CHNO	CHO	CHO	CHO
CHO	CHO	CHO	CHO	CHO	CHO	CHO	CHNO	CHNO	CHNO	CHO	CHO	CHNO	CHNO	CHNO
CCuHO	CHNaO	CHNaO	CHKO	CHKO	CCuHO	CHKO	CHKO	CHN	CClHO	CHN	CHN	CHN	CHN	CHN
CHN	OS	CHKO	CHNaO	CHNaO	CHKO	CCuHO	CHNaO	CClHO	CHOPb	CClHO	CClHO	CClHO	CClHO	CClHO
CHNaO	CHKO	HNO	CHOPb	CHOPb	CHNaO	CHNaO	CCuHO	CHKO	CHN	CCuHO	CCuHO	CHOS	CHOS	CHOS
Cu	CCuHO	OS	HOSb	HOSb	CHOPb	HNOP	CHN	CCuHO	CCuHO	CHOPb	CHOPb	CCuHO	BrCHO	BrCHO
NaOS	CCaHO	CCaHO	HNO	CCuHO	NaOW	CHOPb	CHOS	CClH	CHKO	CHOS	CClHNO	CClHNO	CClHNO	CClHNO
OS	ClHg	CoHO	OS	HNOS	HOSb	NaOW	HNOP	CHNaO	CHOS	CClHNO	CHOS	CHNOS	CHNOS	CHNOS
CCuO	CHN	CCuHO	CCuHO	HNOSe	HNOS	HNOS	CHOPb	CHOS	CClHNO	CHKO	CHNaO	CHOPb	CClH	CClH
CH	Cu	ClHg	HNOS	HNO	KS	HOSb	HNOS	CHOPb	CClH	CH	CH	CClH	CCuHO	CH
CHgNO	NaOS	NaOS	OSb	OS	HNOSe	KS	CHNS	HNOS	CHNaO	CClH	CHKO	CH	CH	BrCH
CNaO	CuO	AsO	CCaHO	OSb	OS	OS	CCaHO	CH	HNOS	CHNaO	CClH	CHNaO	CHOPb	BrCHNO
ClCu	HNO	CoO	CoHO	CCaHO	CrO	CrO	NaOW	CClHNO	CH	CHNS	CHNS	CHNS	CHNaO	CCuHO
ClCuHO	AsHO	NO	ClHg	NO	CFeN	AsHNaO	KS	CHNOS	BrCHO	HNOS	BrCHO	BrCHO	BrCH	CHNaO
ClH	AsO	Fe	AsO	CrO	CHNS	CHN	OS	FeHKOS	CHNOS	BrCHO	CHNOS	CHKO	CHNS	CHNS
ClHN	CO	AsHNiO	HNOPb	HHgOSe	SSb	FeHKOS	CrO	HNOP	CHNS	CHNOS	HNOS	AgCHNO	CHKO	CHOPb
ClHNaOPt	ClHNTi	HOSSn	NaOS	CoHO	HNO	HNOSe	FeHKOS	CCaHO	FeHOS	CCuHNO	CHMgO	CHIN	CHIN	CClHN
ClNa	ClO	CHN	CoO	ClHg	OSb	CFeN	NO	HOS	ClHNPt	FeHOS	CCuHNO	HNOS	CHS	CHIN
ClSn	CoHO	Cu	NO	AsO	CCaHO	CHNS	HOS	CHHgO	HOS	ClHNPt	AgCHNO	CHMgO	BrCHNO	CHS
CuHO	CoO	CuO	Fe	HNOPb	NO	SSb	CHNOS	CHNS	CHHgO	HOS	FeHOS	CCaHO	AgCHNO	AgCHNO

4 Evolution of some molecular fragments

We determined the temporal appearance of the molecular fragments depicted in Figure S4 by exploring the connection tables of the compounds reported in the database between 1800 and 1869. A connection table is a "listing of atoms and bonds, and other data, in tabular form" (5).

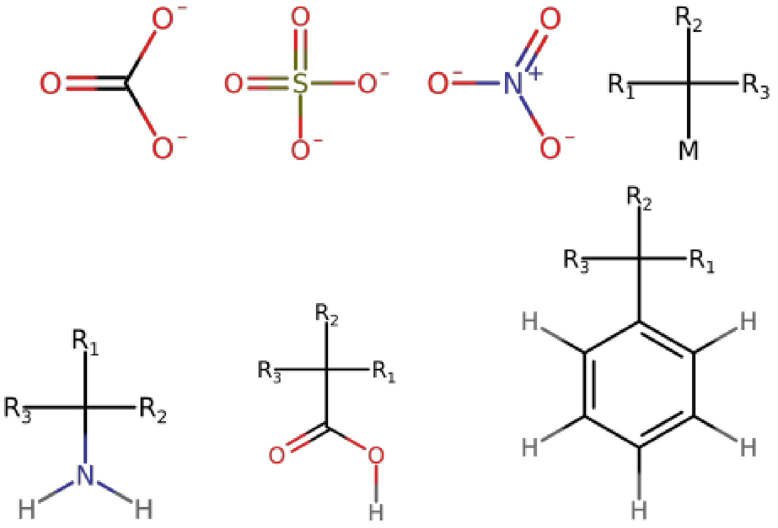

Figure S4: Molecular fragments used to explore the evolution of the chemical space.

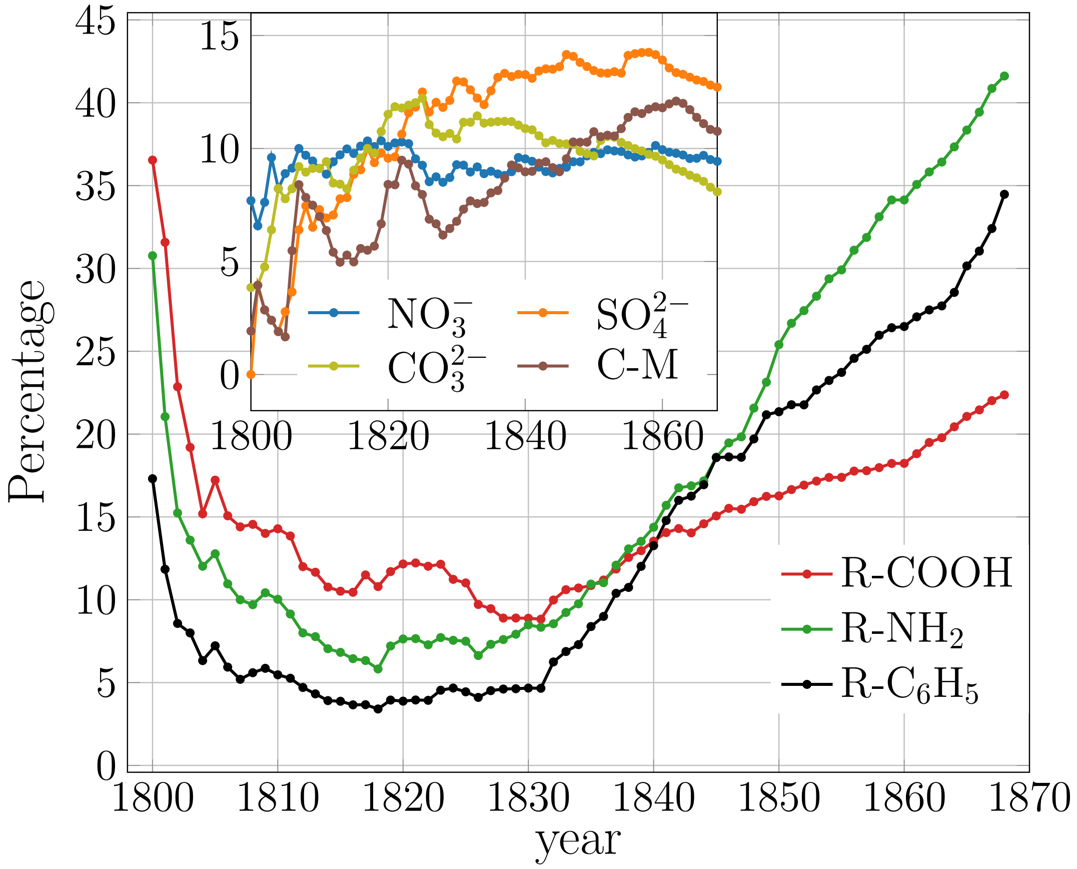

Figure S5: Distribution of typical organic, inorganic and organometallic molecular fragments. C-M means that at least one bond between C and M (see below) is reported on the table. It does not necessarily mean that C and M are bonded by a single covalent bond. The structures of the analysed molecular fragments are shown in Figure S4. In the inset, M stands for a metal, with M={Li, Be, Al, Si, Fe, Co, Zn, As, Rh, Sb, Pt, Hg, Tl, Pb, Bi}

5 Quantifying similarity among chemical elements

We quantified the similarity of element x regarding element y as the fraction of substances of x in whose formulae x can be replaced by y yielding a formula that is part of the chemical space. Hence, for an element x having s_x substances in the chemical space, which are gathered as $\{s_x^1, s_x^2, \ldots, s_x^n\}$, there is an associated multiset of formulae $F_x = \{f(s_x^1), f(s_x^2), \ldots, f(s_x^n)\}$, where $f(s_x^i)$ is the arranged formula of substance i containing element x. Arranged formulae are assigned to a reference element, whose similarity regarding other elements is to be calculated. These formulae are found by replacing the reference element x in their formulae by the symbol X. The resulting formula is lexicographically ordered for the sake of comparison with other formulae. For example, the arranged formula of NH_2Cl, with Cl as reference element, is H_2NX. Figure 2 (main text) shows a toy-example for the similarity between Cl and Br ($s(\mathrm{Cl} \rightarrow \mathrm{Br})$) and Br and Cl ($s(\mathrm{Br} \rightarrow \mathrm{Cl})$). There, Cl has eight substances in the chemical space, namely {NH_2Cl, $HAuCl_4$, VCl_3O, K_3CrCl_6, CH_2CCl_2, NaCl, $KClO_3$}. In turn, Br has seven substances: {CH_2CBr_2, CHBrCHBr, NaBr, $KBrO_3$, $MoBr_4$, $HgOBr_2$}. The multiset of arranged formulae for Cl is F_{Cl} = {H_2NX, $AuHX_4$, OVX_3, CrK_3X_6, $C_2H_2X_2$, NaX, KO_3X} and for Br it is F_{Br} = {$C_2H_2X_2$, $C_2H_2X_2$, NaX, KO_3X, MoX_4, $HgOX_2$}. Note that in both cases all formulae have multiplicity one, except $C_2H_2X_2$, which has multiplicity two in F_{Br}. This shows that Br has two substances with arranged formulae $C_2H_2X_2$. They are CH_2CBr_2 and CHBrCHBr, which are isomers participating in single-step reactions before 1869. Note that $C_2H_2X_2$ has multiplicity one for Cl, as only CH_2CCl_2 had participated in single-step reactions by 1869. Thus, F_{Br} can be rewritten as $F_{\mathrm{Br}} = \{C_2H_2X_2^2, \mathrm{NaX}^1, KO_3X^1, \mathrm{MoX}_4^1, \mathrm{HgOX}_2^1\}$, which, in general, has the form $F_x = \{f_i^{m_x(i)}\}$, where f_i is the i-th arranged formulae of element x whose formula multiplicity is $m_x(i)$. By multiplicity of a formula is meant the number of times the formula shows up in the multiset, that is the number of times the formula is found in the chemical space of element x.

With the list of arranged formulae for elements x and y, we can calculate $s(x \rightarrow y)$ as:

$$s(x \rightarrow y) = \frac{|F_x \cap F_y|}{|F_x|}$$

As $|F_x|$ amounts to counting the multiplicities of arranged formulae of x, then $|F_x| = \sum m_x(i)$. Likewise, finding the common arranged formulae between F_x and F_y amounts to counting the minimum multiplicity of formulae that appear in both multisets F_x and F_y: $|F_x \cap F_y| = \sum \min(m_x(i), m_y(i))$, which for $|F_{\mathrm{Cl}} \cap F_{\mathrm{Br}}| = \min(m_{\mathrm{Cl}}(C_2H_2X_2), m_{\mathrm{Br}}(C_2H_2X_2))+$ $\min(m_{\mathrm{Cl}}(\mathrm{NaX}), m_{\mathrm{Br}}(\mathrm{NaX})) + \min(m_{\mathrm{Cl}}(KO_3X), m_{\mathrm{Br}}(KO_3X)) = \min(1,2) + \min(1,1)+$ $\min(1,1) = 3$. Therefore, the similarities $s(\mathrm{Cl} \to \mathrm{Br})$ and $s(\mathrm{Br} \to \mathrm{Cl})$ are calculated as $s(\mathrm{Cl} \to \mathrm{Br}) = 3/7$ and $s(\mathrm{Br} \to \mathrm{Cl}) = 3/6$.

6 Similarity values among chemical elements

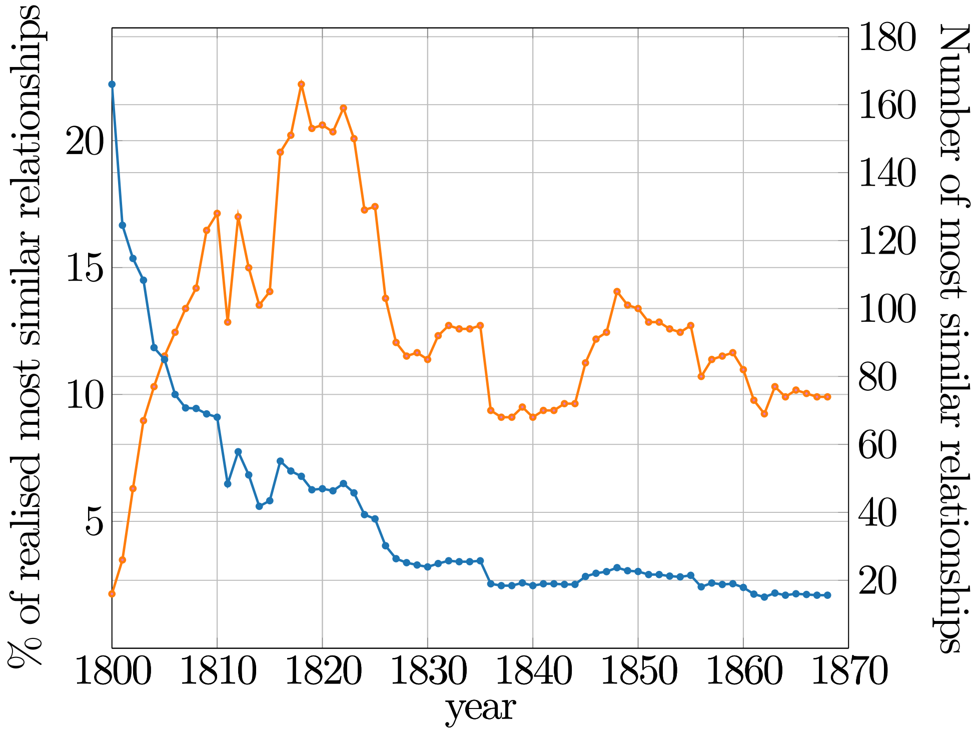

Figure S6: Number (orange) and percentage (blue) of realised "most similar" relationships. Orange curve shows the number of most similar relationships among elements for the SCE of year y (s_y), that is the number of arrows in networks of Figure 3a-c (main text). The percentage of realised most similar relationships among chemical elements over time (blue curve) is calculated as $(s_y/u_y) \times 100$, where u_y is the possible number of relationships for year y, corresponding to $u_y = n_y \times n_y$, with n_y the number of known elements in year y.

7 Periodic systems by Meyer and Mendeleev up to 1869/70

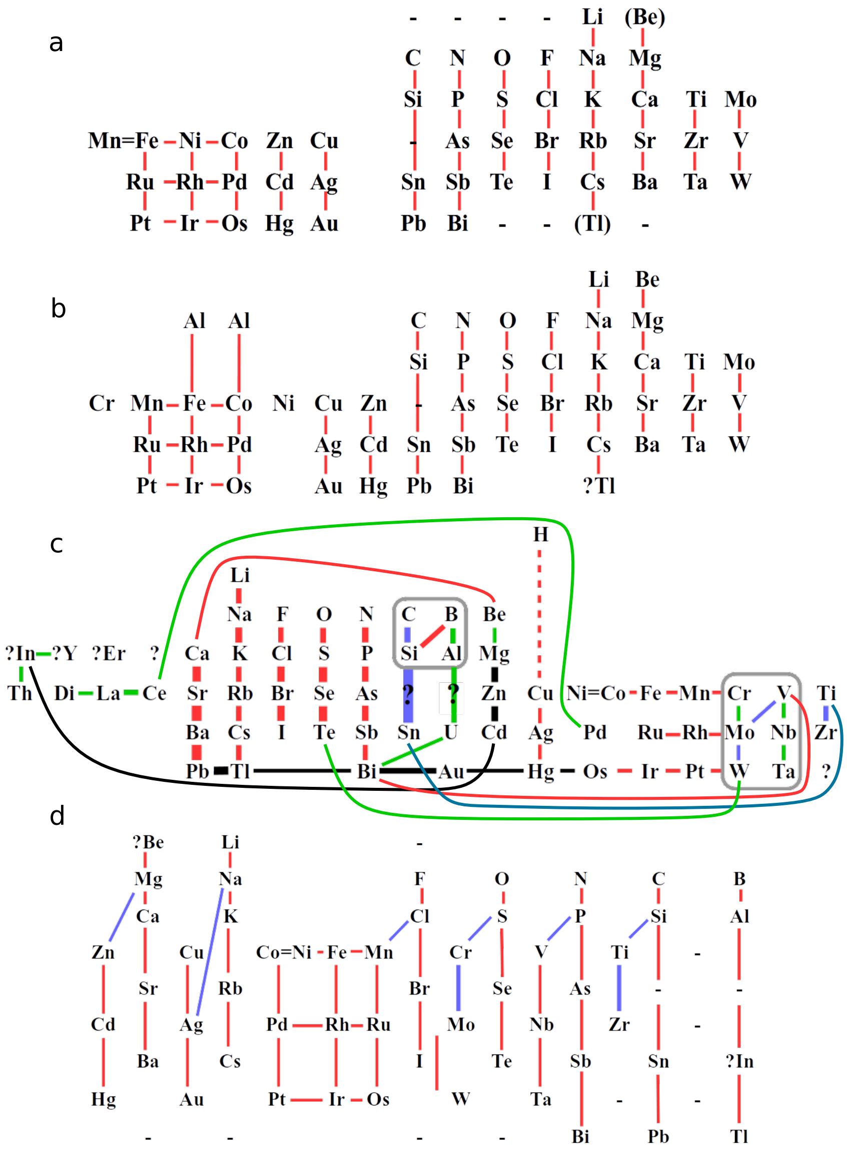

Figure S7: Systems of chemical elements by Meyer (a: 1864, gathering together his three separate tables; b: 1868; d: 1869/70) (6, 7, 4) and Mendeleev (c: 1869, rotated and reflected for the sake of comparison with the other SCEs) (1). Element symbols are updated to current notation. Lines and boxes indicate similarities. The complete list of similarities for Mendeleev is found in Table S2. Line widths are proportional to the number of times the similarity is discussed by each author. Line colours are used only for the sake of clarity

8 Similarities as discussed by Mendeleev (1869)

Table S2: Similarities as mentioned by Mendeleev in his extended 1869 publication (8). Red entries correspond to similarities Mendeleev thought did not exist and the blue one to "not so well studied."

Bi,V,Sb,As	P,N	Te,Se,S	Si,Ti,Zr,Sn
I,F	Mg,Zn,Cd	Cu,Ag	Tl,alkali metals
Pb,Ba,Sr,Ca	Pd,Rh,Ru	Os,Ir,Pt	Si,B
Ag,Cu,Hg	Sb,Bi	Tl,Cs	Halogens
Alkaline-earth metals	N group	S group (in part)	Pt companions
Ce companions	Li,K,Na	Ca,Sr,Ba	Cl,I,Br
O,S,Se,Te	N,P,As,Sb	Mg,Zn,Cd	Ag,Pb,Hg
P,As,Sb	Os,Pt,Ir	W,V,Mo	Ta,Sn,Ti
Si,B,F	O,N,C	Cr,Ni,Cu	Be,Zr,U
N,P,As	Na,K,Rb,Cs	F,Cl,Br,I	C,Si,. . .,Sn
V,Nb,Ta	Cr,Mo	Sr,Ba,Pb	Li,Na,K,Rb,Cs,Tl
Li,Na,K,Cu,Rb,Ag,Cs,Tl	Be,Mg,Ca,Zn,Sr,Cd,Ba,Pb	B,Al,U,Bi	C,Si,Ti,Zr,Sn
N,P,V,As,Nb,Sb,Ta	O,S,Se,Te,W	Be,Ca,Sr,Ba,Pb	V,P,Sb,As
Ti,Si,Sn	Cr,S,Te	Mn,Cl,Br	Si,Ti,?
P,V,As	S,Cr,Se	Cl,Mn,Br	As,Sb,Nb
Nb,V,Sb	In,Mg,Zn,Cd	Zr,C,Sn	{Mg,Zn,Cd},{Ca,Sr,Ba}
Y,Th,In	Co,Ni,Cr,Mn,Fe	Ce,La,Di,Pd,Rh,Ru	Pt,Ir,Os
Mg,{Ca,Sr,Ba}	Pb,Tl,Bi,Au,Hg,Pt,Ir,Os,W	Pb,Tl	Bi,Au
Hg,Pt	H	H,Cu,Ag,Hg	C,B,Si,Al
Ba,Pb,Tl	V,Cr,Nb,Mo,Ta,W	B,Al,U	

9 Distribution of similarity values among chemical elements

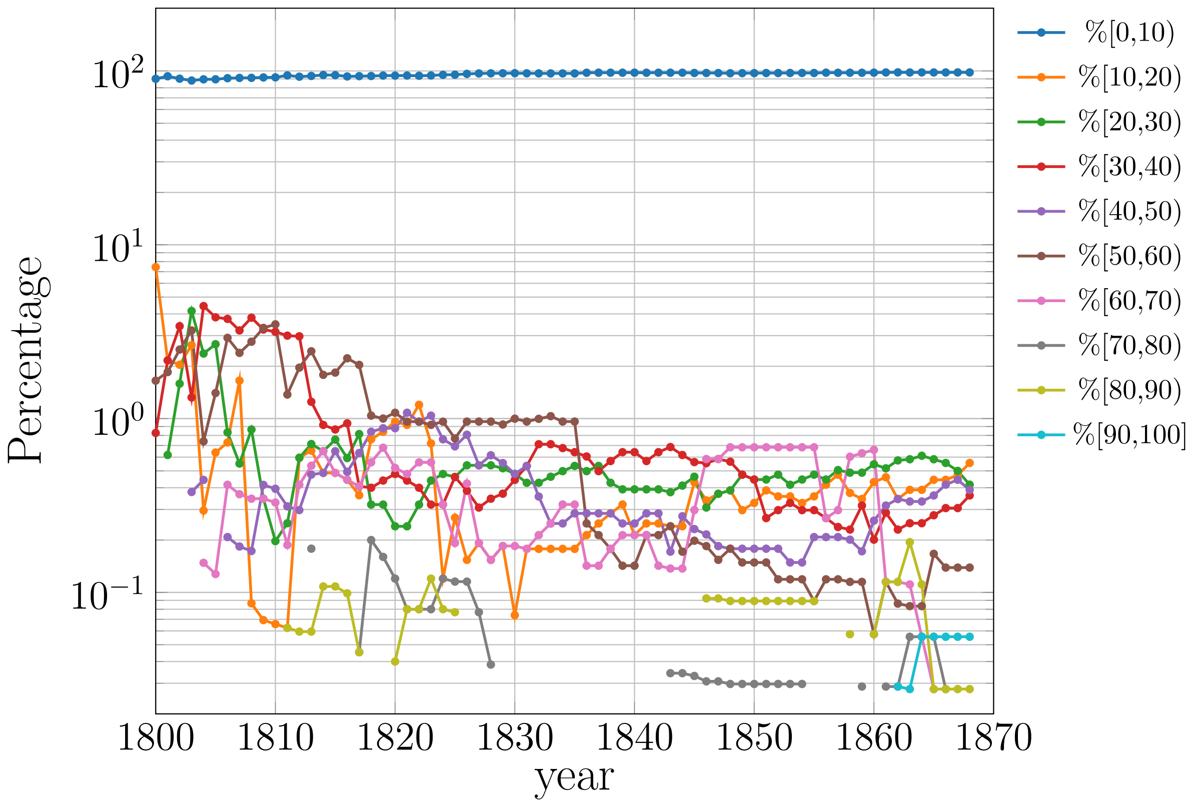

Figure S8: Distribution of similarity values in the SCEs. Each series plots the percentage of element pairs with similarity values in the given interval ($[0, 10), [10, 20), \ldots, [90, 100]$)

10 Perturbing the chemical space and its effect on the system of chemical elements

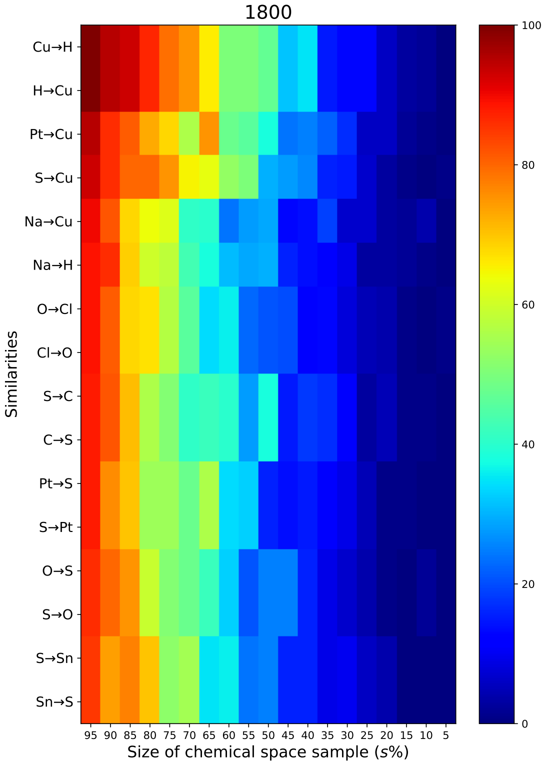

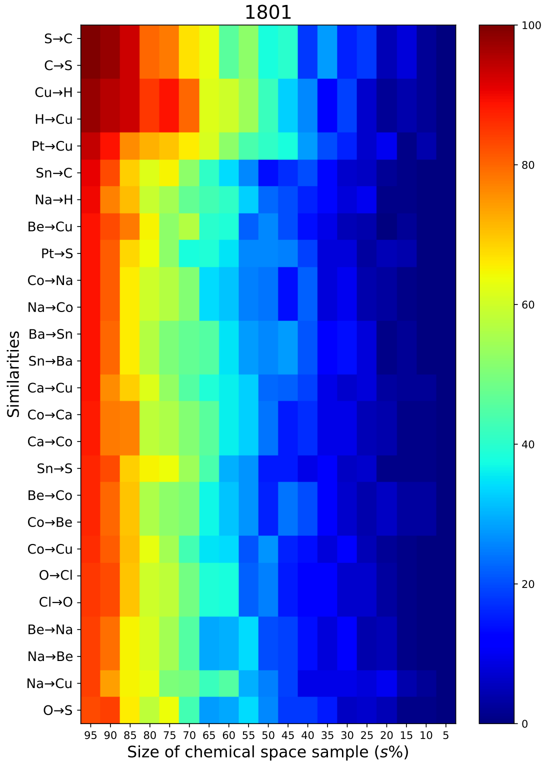

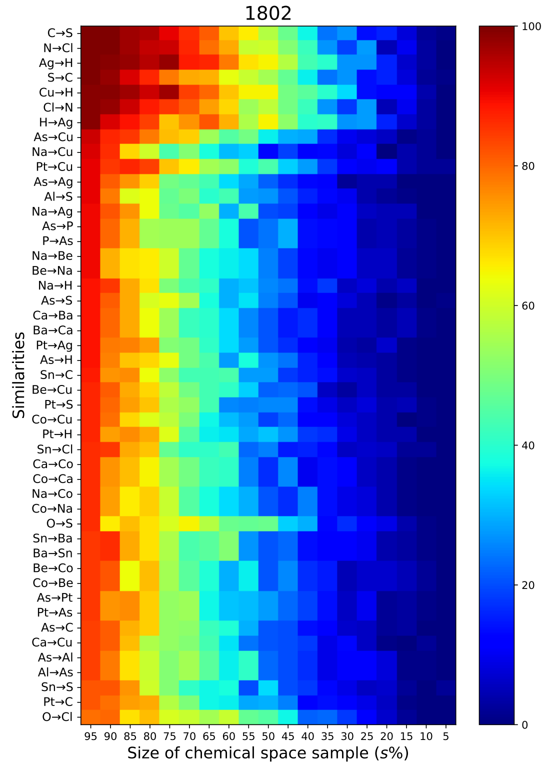

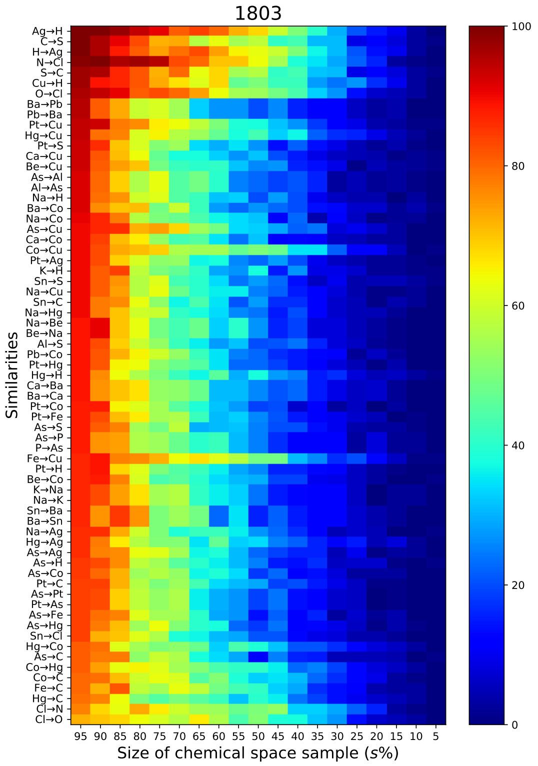

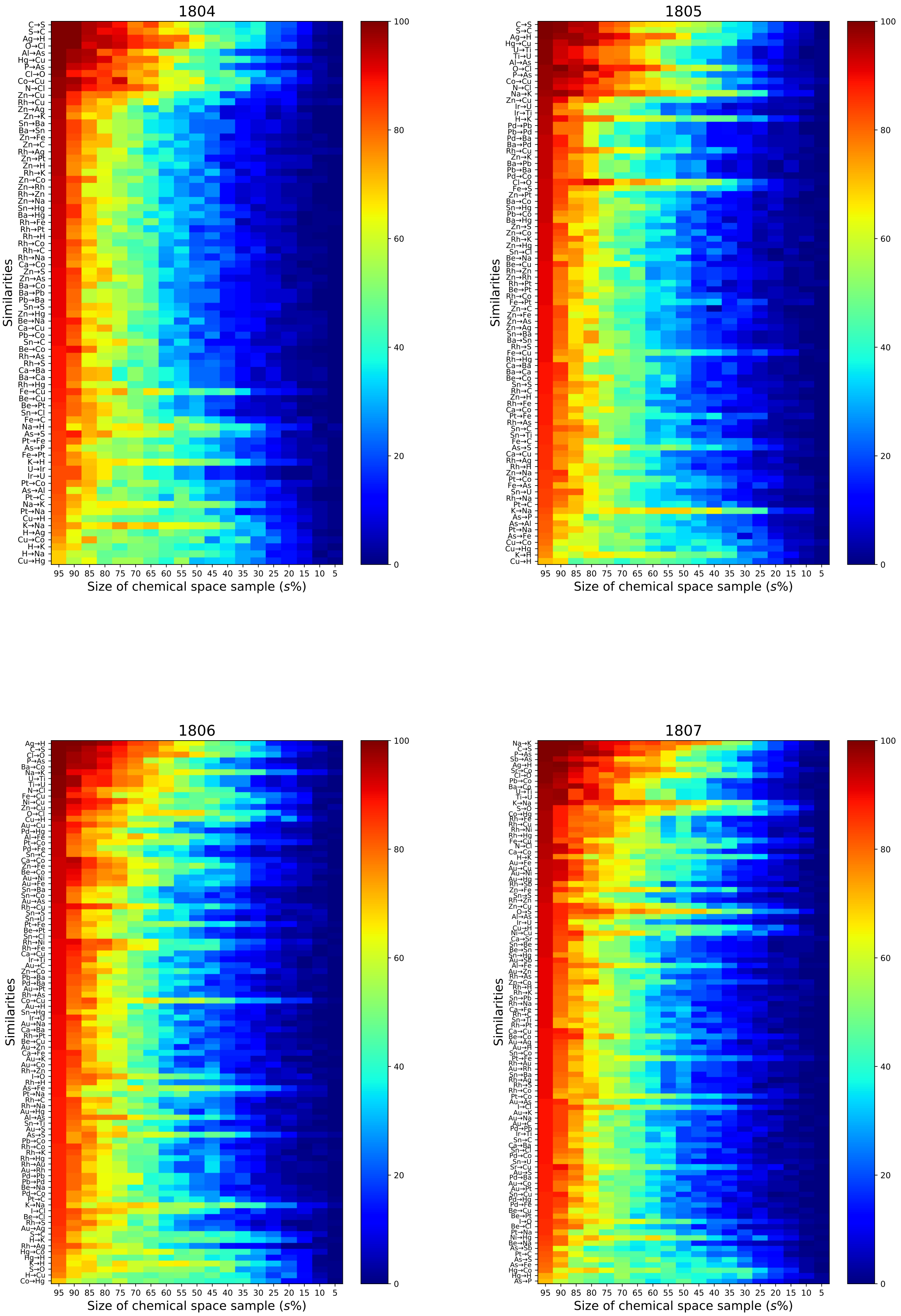
1804
1805
1806
1807
Similarities
Size of chemical space sample (s%)
95 90 85 80 75 70 65 60 55 50 45 40 35 30 25 20 15 10 5
0
20
40
60
80
100

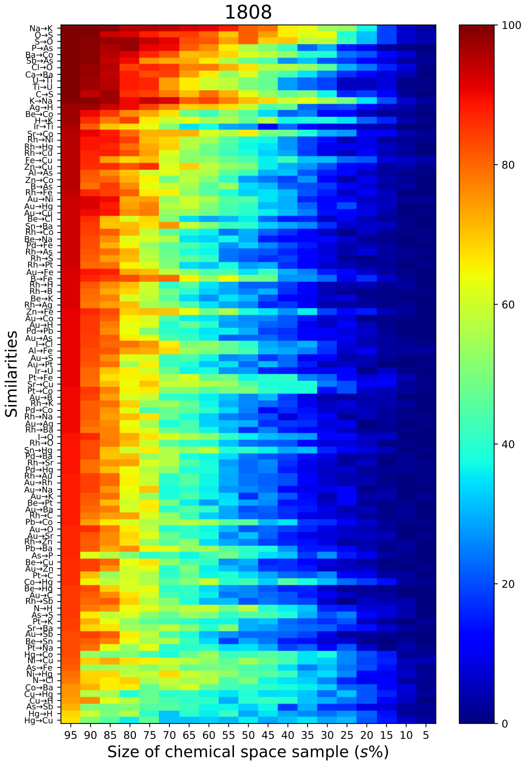
1808
Similarities
Size of chemical space sample (s%)

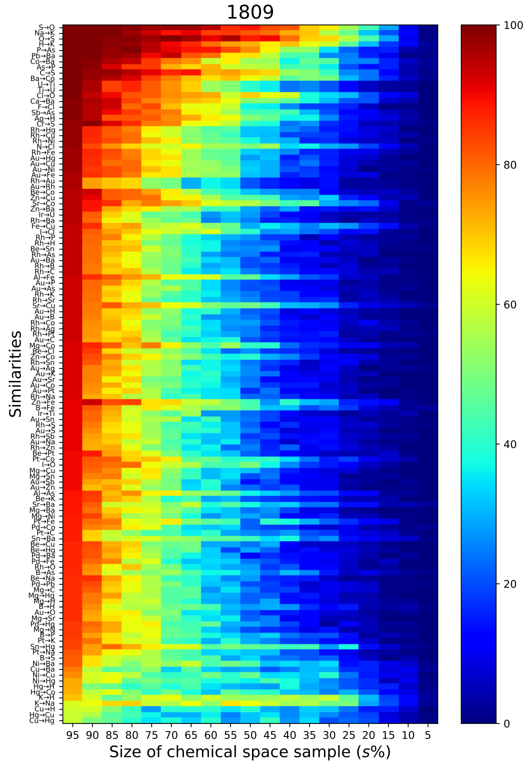
1809
Similarities
Size of chemical space sample (s%)

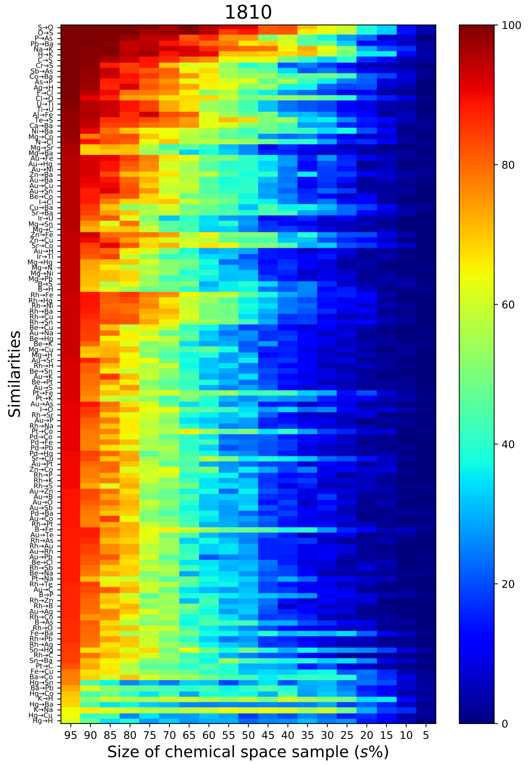
1810
Similarities
Size of chemical space sample (s%)

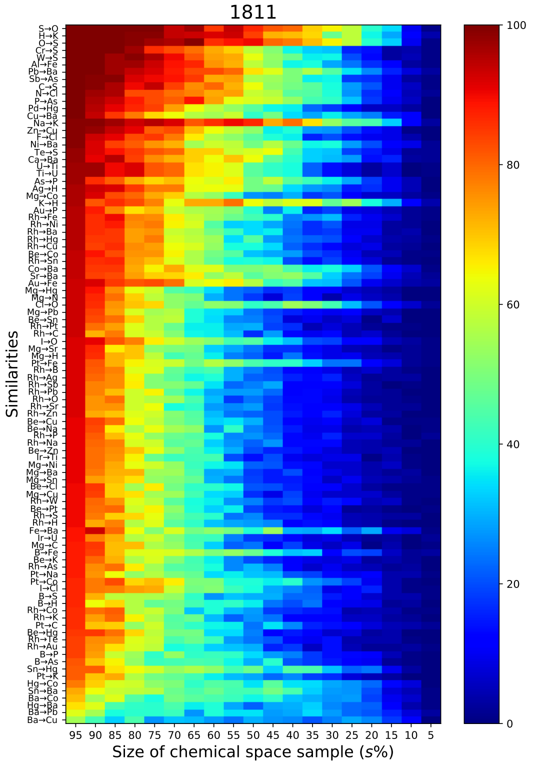
1811
Similarities
Size of chemical space sample (s%)

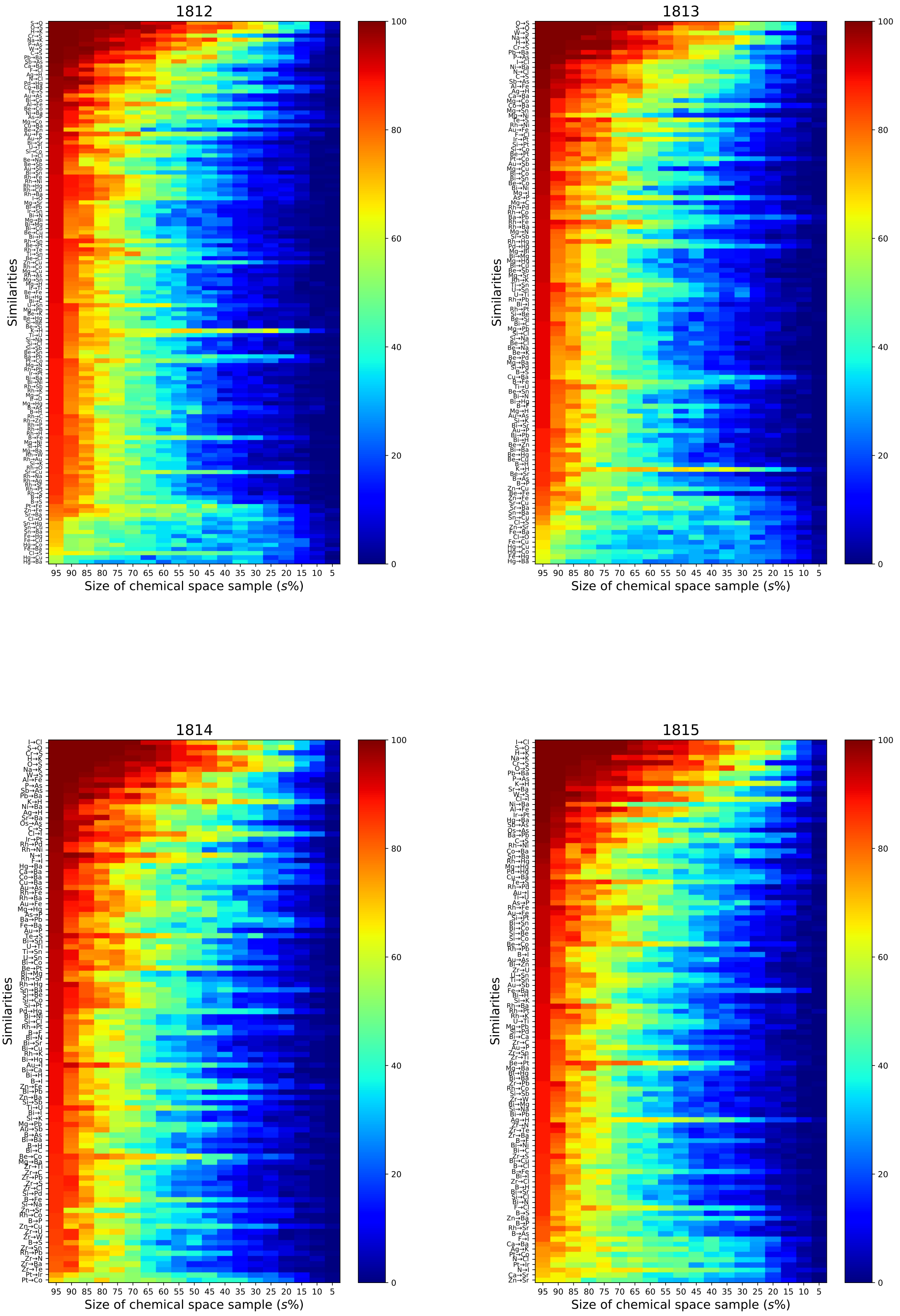
1812
1813
1814
1815
Similarities
Size of chemical space sample (s%)
95 90 85 80 75 70 65 60 55 50 45 40 35 30 25 20 15 10 5
100
80
60
40
20
0

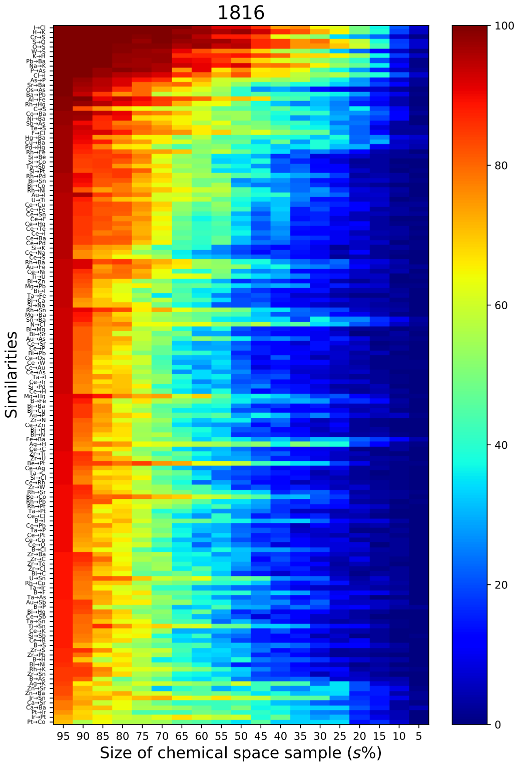
1816
Similarities
95 90 85 80 75 70 65 60 55 50 45 40 35 30 25 20 15 10 5
Size of chemical space sample (s%)
0 20 40 60 80 100

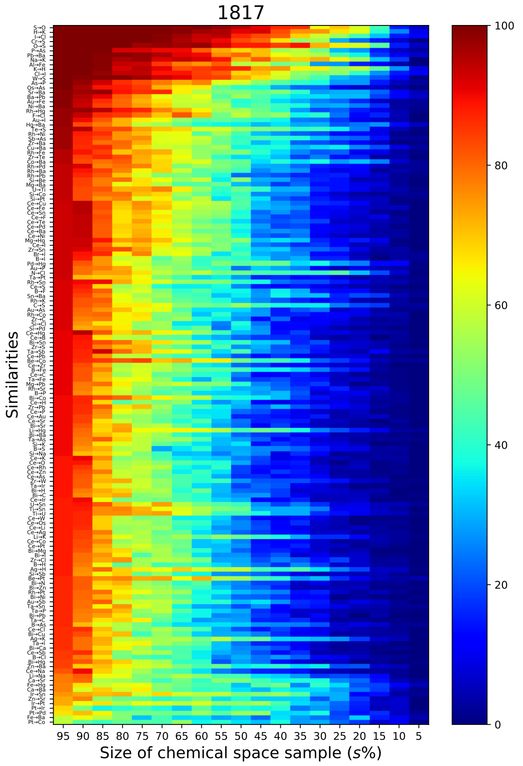
1817
Similarities
95 90 85 80 75 70 65 60 55 50 45 40 35 30 25 20 15 10 5
Size of chemical space sample (s%)
0 20 40 60 80 100

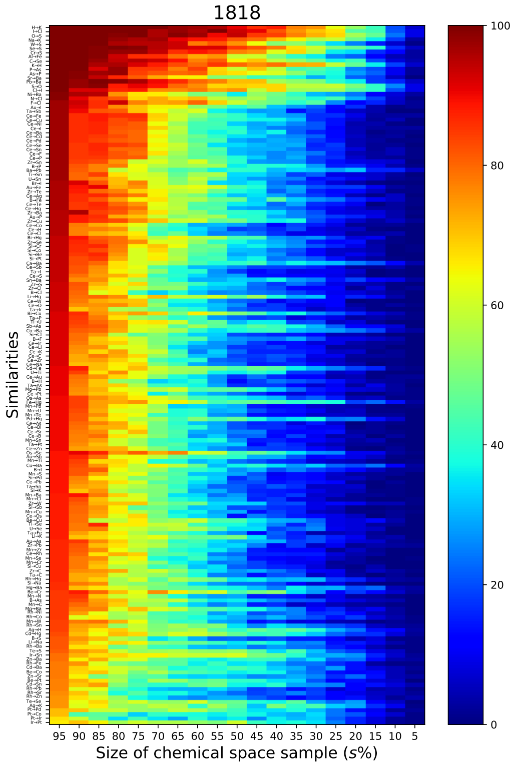
1818
Similarities
95 90 85 80 75 70 65 60 55 50 45 40 35 30 25 20 15 10 5
Size of chemical space sample (s%)
0 20 40 60 80 100

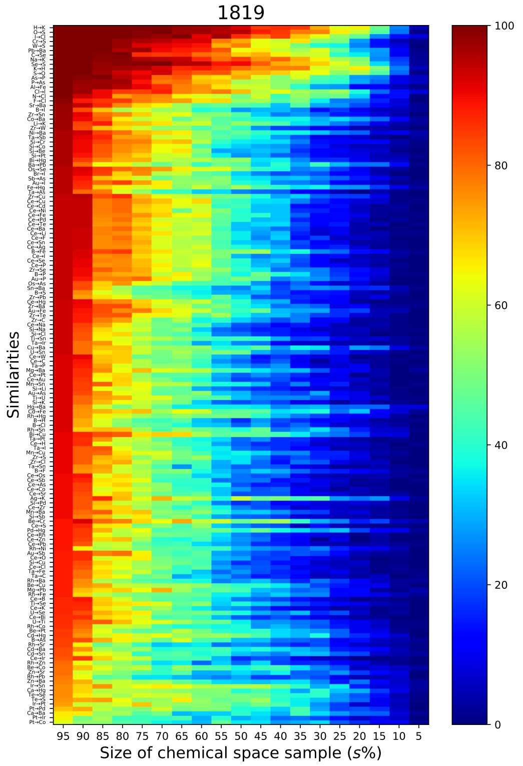
1819
Similarities
95 90 85 80 75 70 65 60 55 50 45 40 35 30 25 20 15 10 5
Size of chemical space sample (s%)
0 20 40 60 80 100

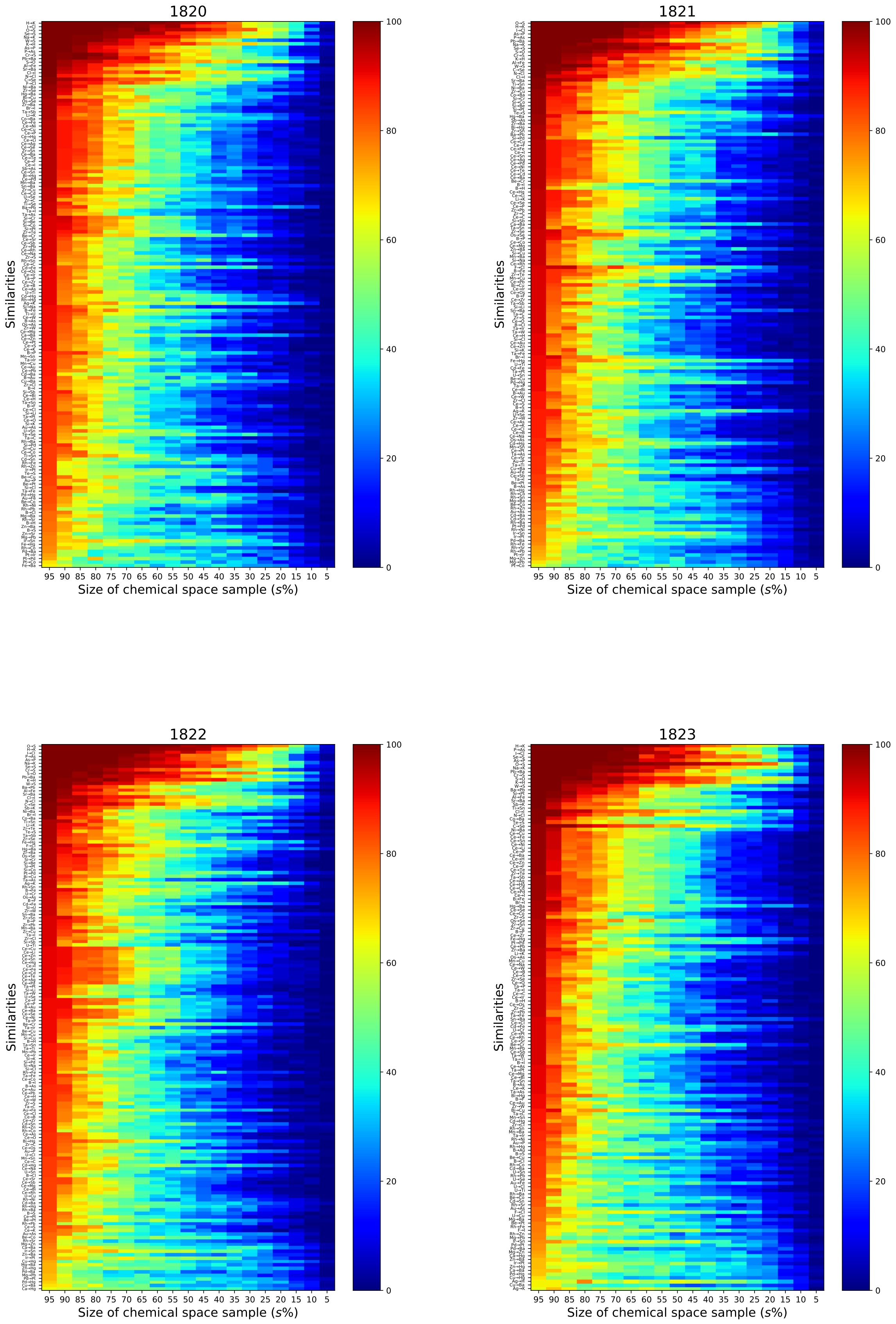

1820
1821
1822
1823
Similarities
Size of chemical space sample (s%)
95 90 85 80 75 70 65 60 55 50 45 40 35 30 25 20 15 10 5
0 20 40 60 80 100

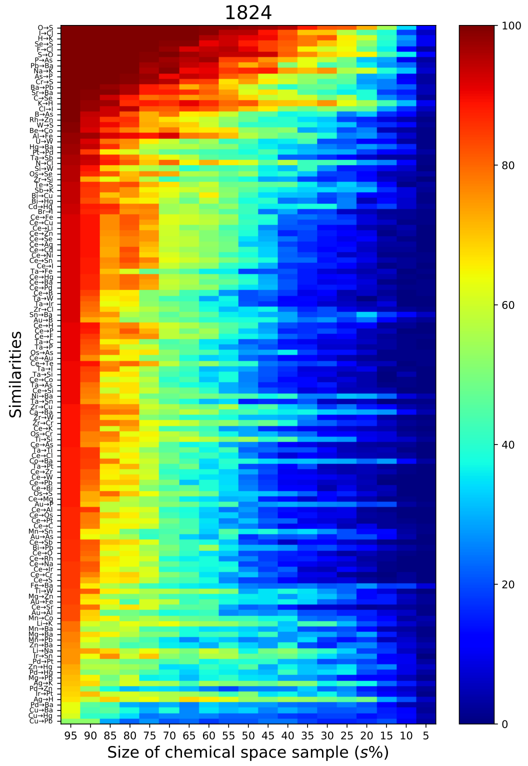
1824
Similarities
Size of chemical space sample (s%)
95 90 85 80 75 70 65 60 55 50 45 40 35 30 25 20 15 10 5
0 20 40 60 80 100

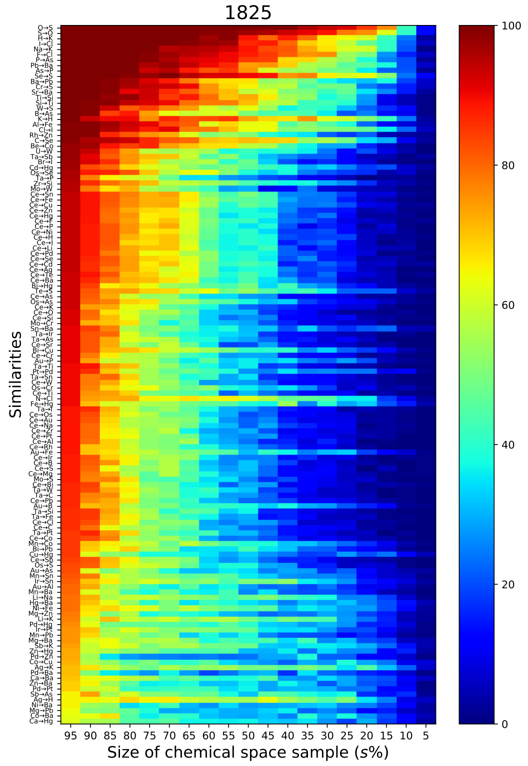
1825
Similarities
Size of chemical space sample (s%)
95 90 85 80 75 70 65 60 55 50 45 40 35 30 25 20 15 10 5
0 20 40 60 80 100

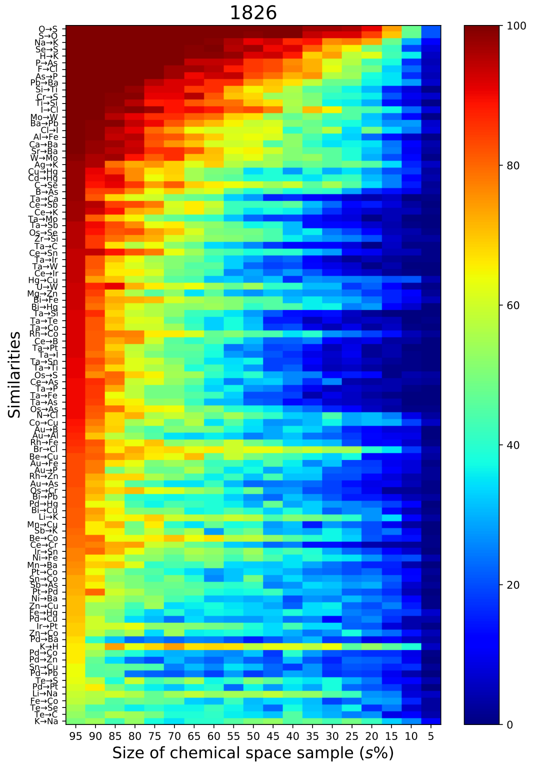
1826
Similarities
Size of chemical space sample (s%)
95 90 85 80 75 70 65 60 55 50 45 40 35 30 25 20 15 10 5
0 20 40 60 80 100

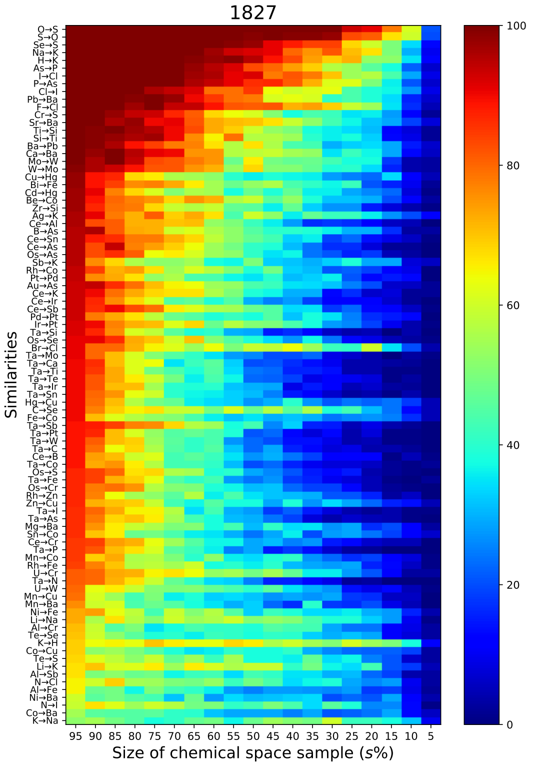
1827
Similarities
Size of chemical space sample (s%)
95 90 85 80 75 70 65 60 55 50 45 40 35 30 25 20 15 10 5
0 20 40 60 80 100

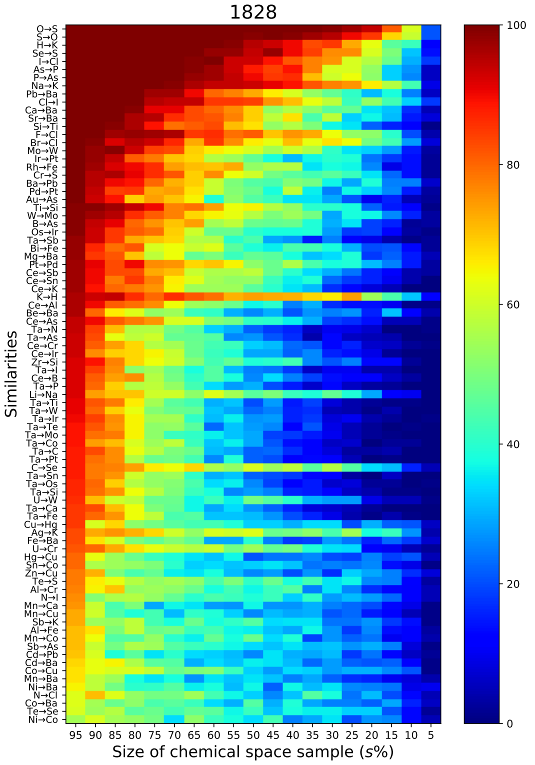
1828
Similarities
Size of chemical space sample (s%)
95 90 85 80 75 70 65 60 55 50 45 40 35 30 25 20 15 10 5
0 20 40 60 80 100

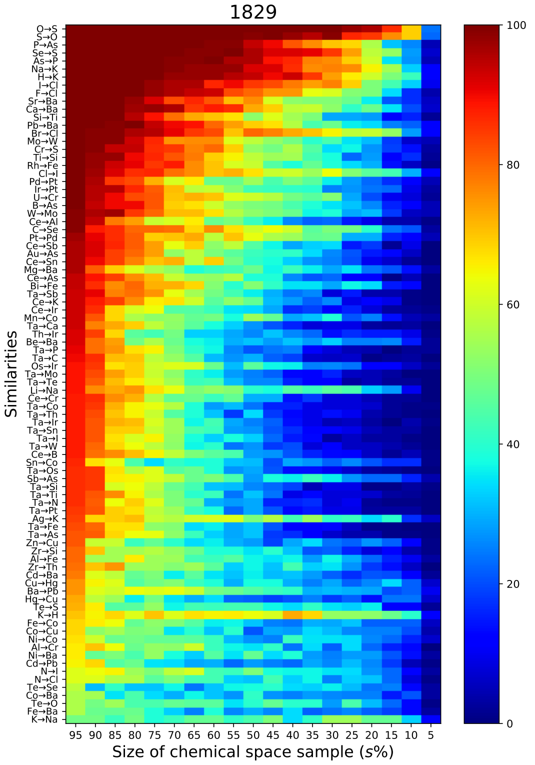
1829
Similarities
Size of chemical space sample (s%)
95 90 85 80 75 70 65 60 55 50 45 40 35 30 25 20 15 10 5
0 20 40 60 80 100

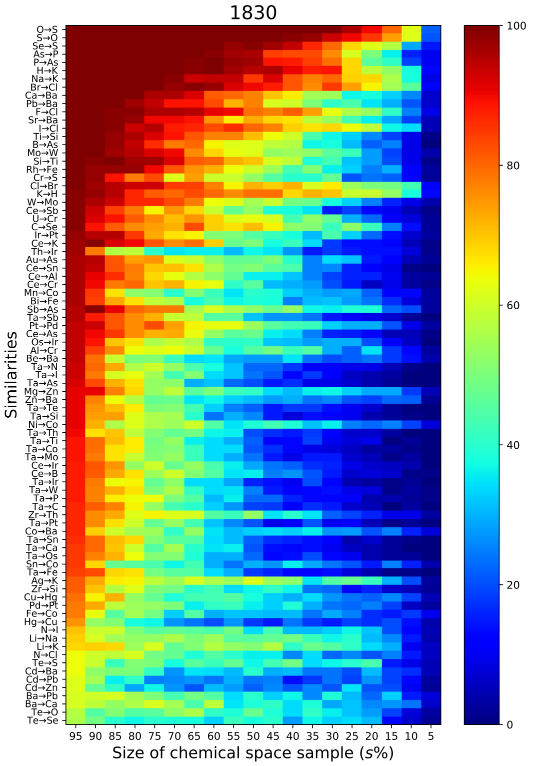
1830
Similarities
Size of chemical space sample (s%)
95 90 85 80 75 70 65 60 55 50 45 40 35 30 25 20 15 10 5
0 20 40 60 80 100

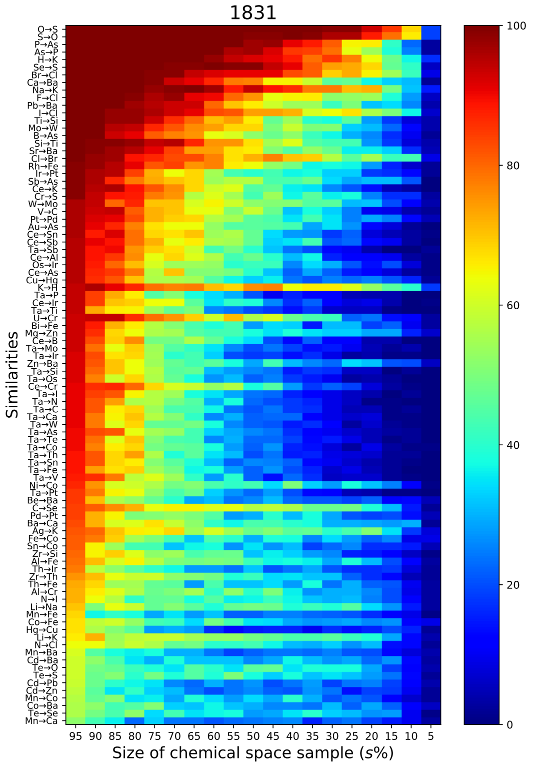
1831
Similarities
Size of chemical space sample (s%)
95 90 85 80 75 70 65 60 55 50 45 40 35 30 25 20 15 10 5
0 20 40 60 80 100

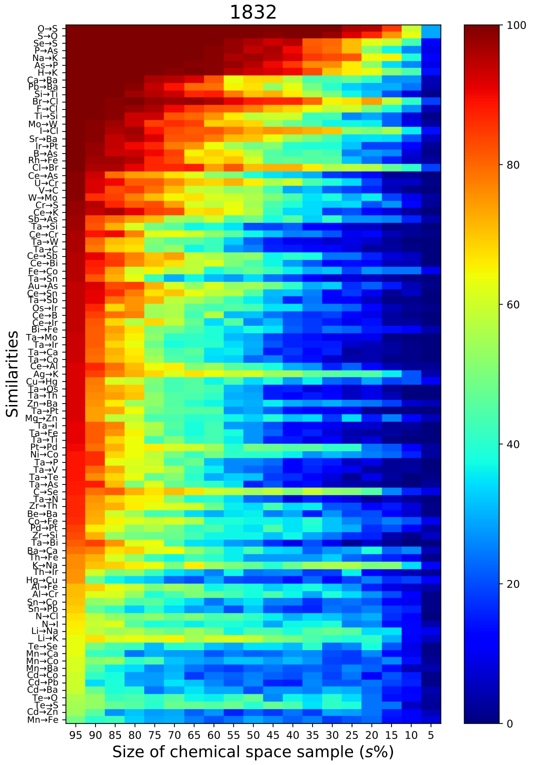
1832
Similarities
Size of chemical space sample (s%)
95 90 85 80 75 70 65 60 55 50 45 40 35 30 25 20 15 10 5
0 20 40 60 80 100

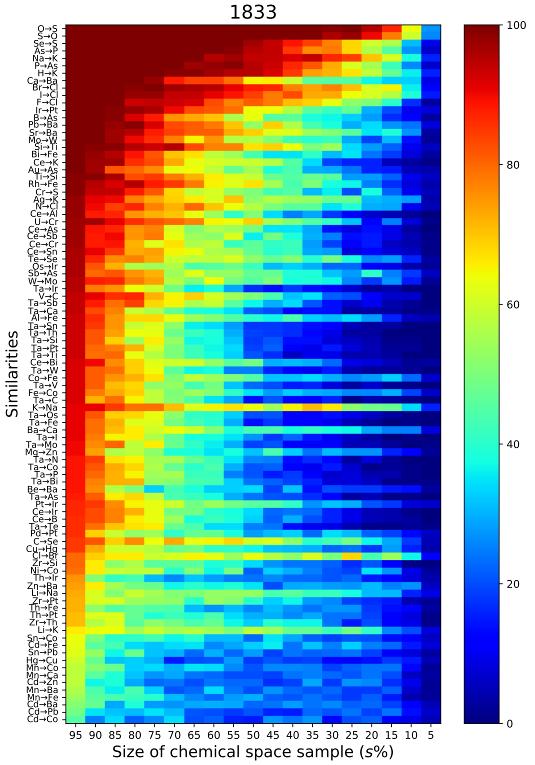
1833
Similarities
Size of chemical space sample (s%)
95 90 85 80 75 70 65 60 55 50 45 40 35 30 25 20 15 10 5
0 20 40 60 80 100

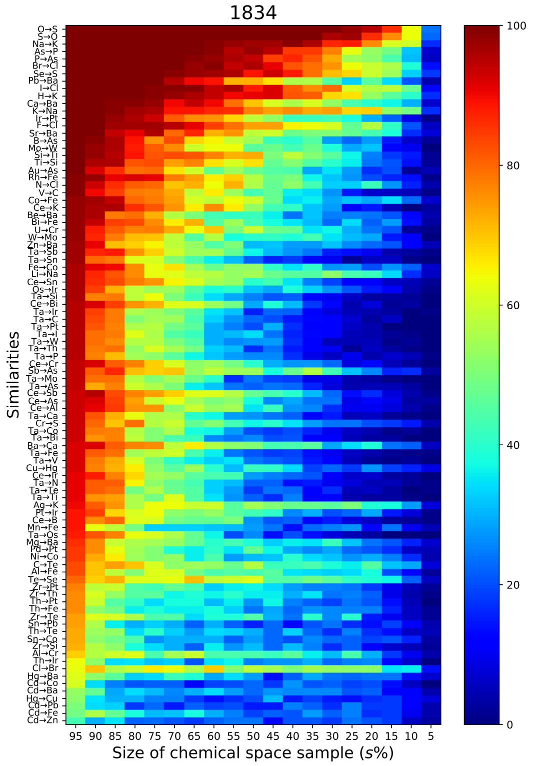
1834
Similarities
Size of chemical space sample (s%)
95 90 85 80 75 70 65 60 55 50 45 40 35 30 25 20 15 10 5
0 20 40 60 80 100

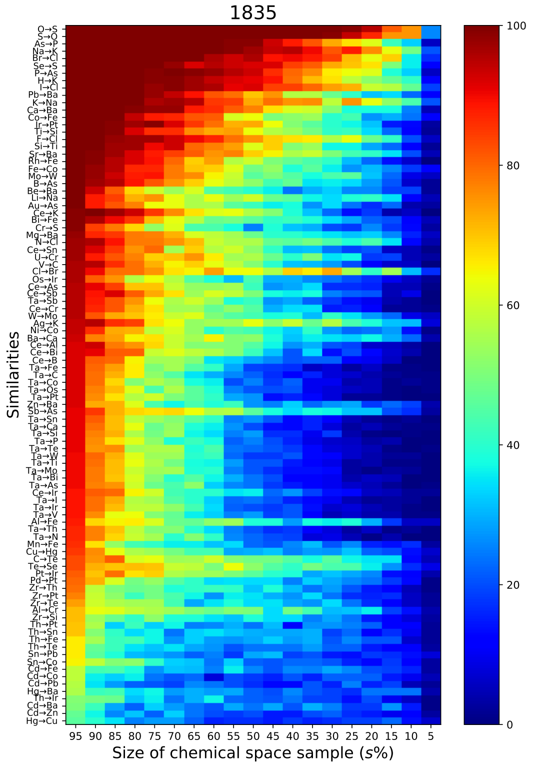
1835
Similarities
Size of chemical space sample (s%)
95 90 85 80 75 70 65 60 55 50 45 40 35 30 25 20 15 10 5
0 20 40 60 80 100

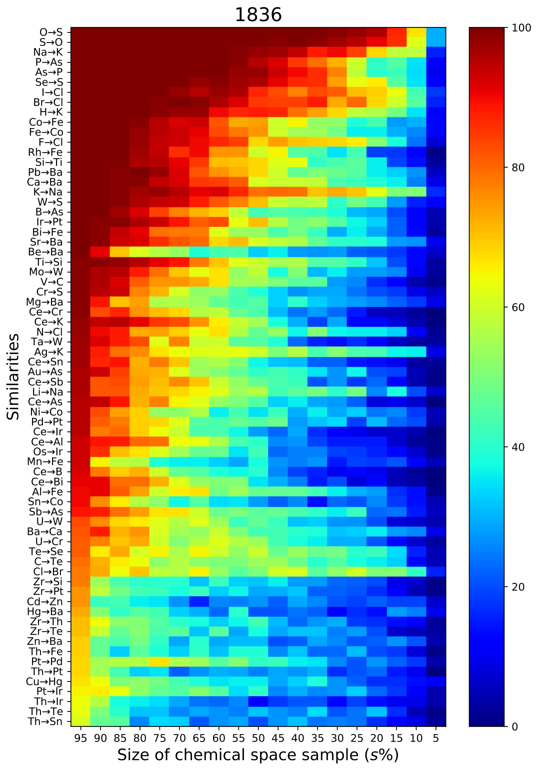

1836
Similarities
Size of chemical space sample (s%)
95 90 85 80 75 70 65 60 55 50 45 40 35 30 25 20 15 10 5
100
80
60
40
20
0

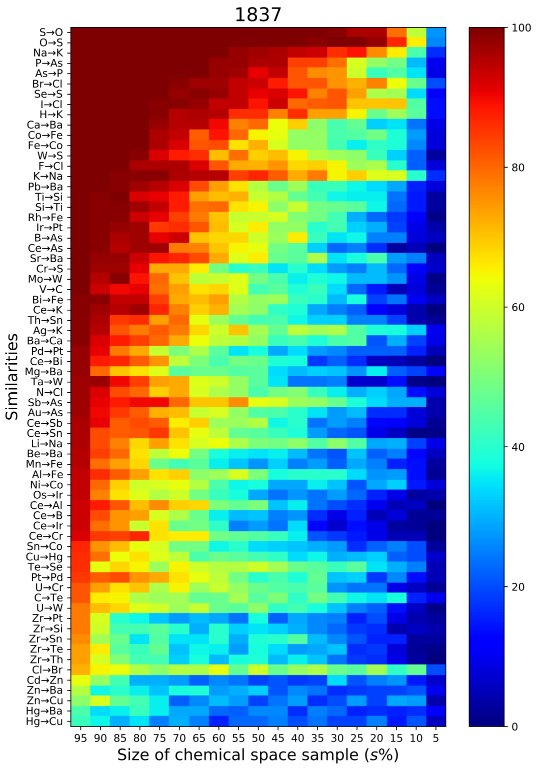

1837
Similarities
Size of chemical space sample (s%)
95 90 85 80 75 70 65 60 55 50 45 40 35 30 25 20 15 10 5
100
80
60
40
20
0

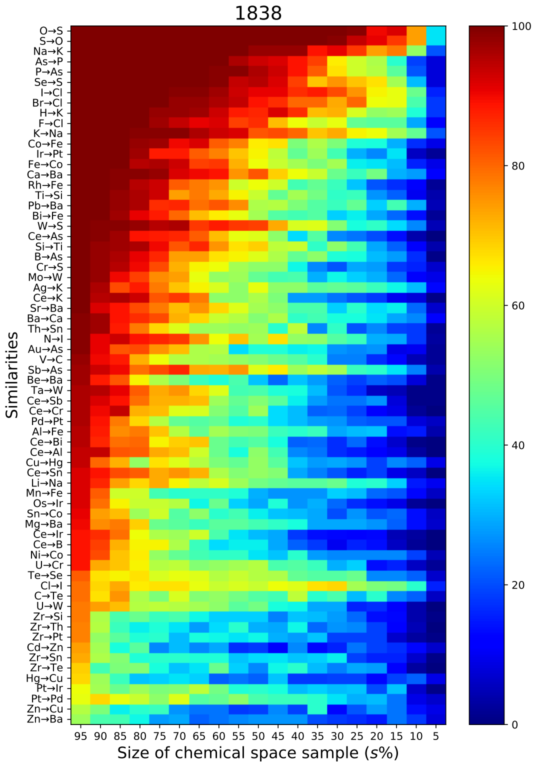

1838
Similarities
Size of chemical space sample (s%)
95 90 85 80 75 70 65 60 55 50 45 40 35 30 25 20 15 10 5
100
80
60
40
20
0

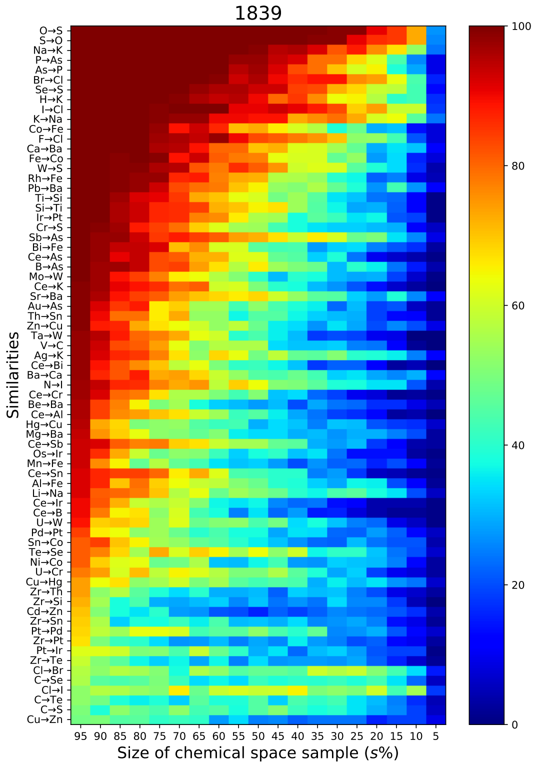

1839
Similarities
Size of chemical space sample (s%)
95 90 85 80 75 70 65 60 55 50 45 40 35 30 25 20 15 10 5
100
80
60
40
20
0

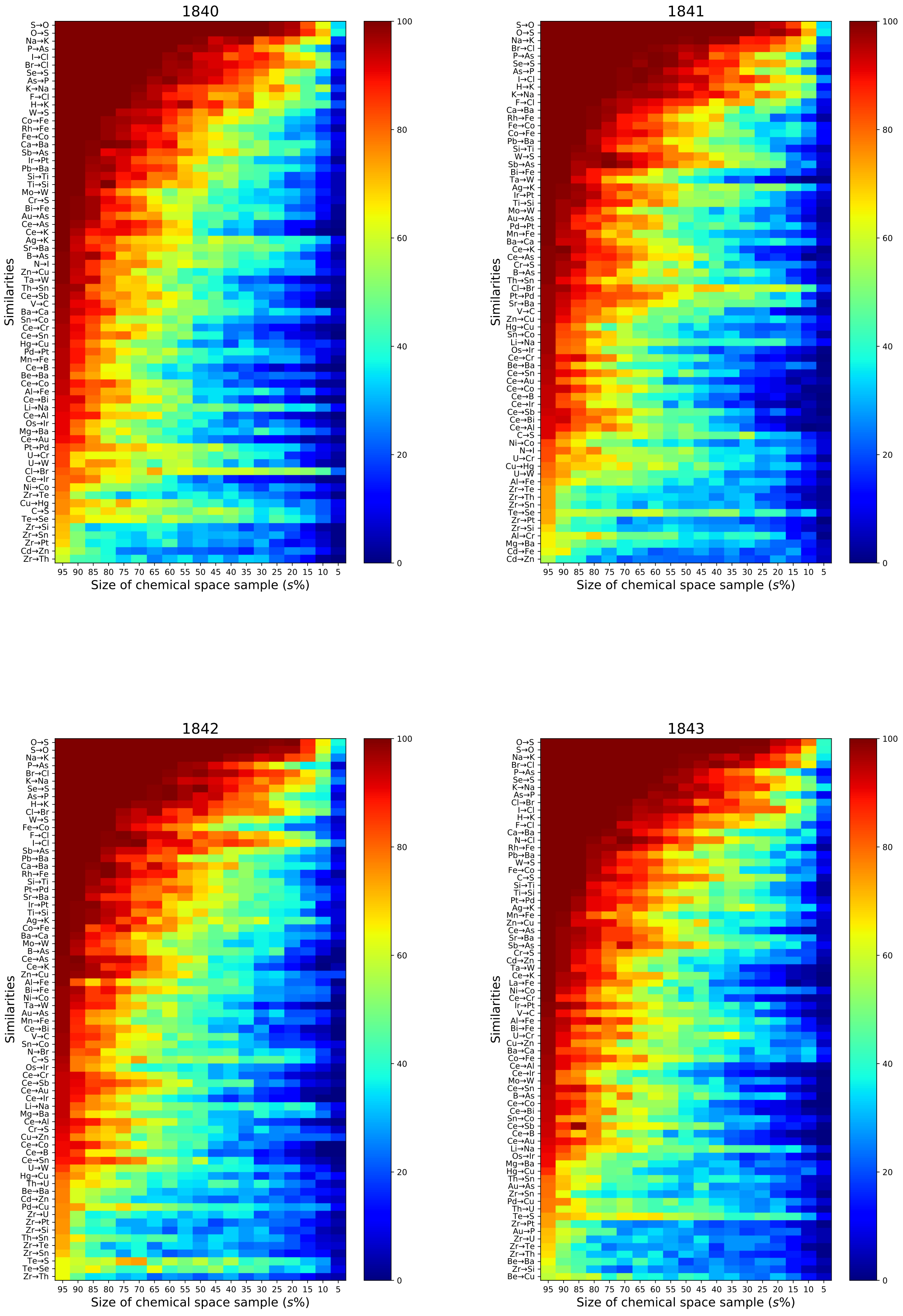

1840
1841
1842
1843
Similarities
Size of chemical space sample (s%)
95 90 85 80 75 70 65 60 55 50 45 40 35 30 25 20 15 10 5
0
20
40
60
80
100

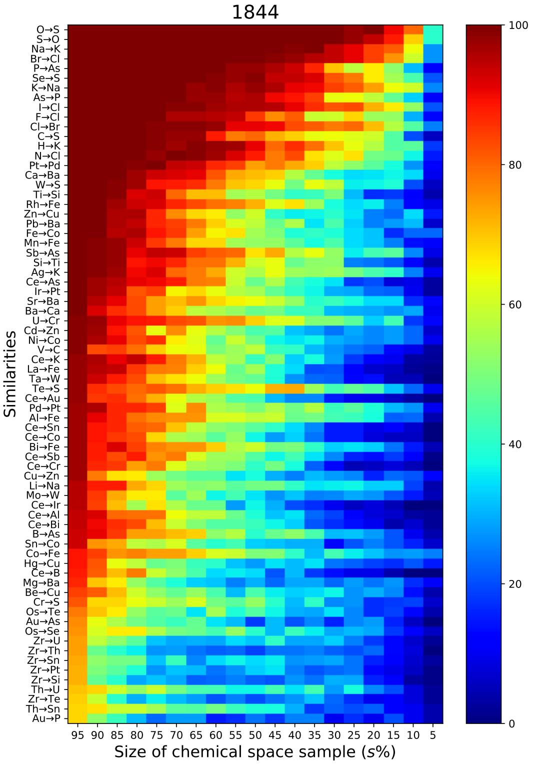
1844
Similarities
Size of chemical space sample (s%)
95 90 85 80 75 70 65 60 55 50 45 40 35 30 25 20 15 10 5
0 20 40 60 80 100
O→S
S→O
Na→K
Br→Cl
P→As
Se→S
K→Na
As→P
I→Cl
F→Cl
Cl→Br
C→S
H→K
N→Cl
Pt→Pd
Ca→Ba
W→S
Ti→Si
Rh→Fe
Zn→Cu
Pb→Ba
Fe→Co
Mn→Fe
Sb→As
Si→Ti
Ag→K
Ce→As
Ir→Pt
Sr→Ba
Ba→Ca
U→Cr
Cd→Zn
Ni→Co
V→C
Ce→K
La→Fe
Ta→W
Te→S
Ce→Au
Pd→Pt
Al→Fe
Ce→Sn
Ce→Co
Bi→Fe
Ce→Sb
Ce→Cr
Cu→Zn
Li→Na
Mo→W
Ce→Ir
Ce→Al
Ce→Bi
B→As
Sn→Co
Co→Fe
Hg→Cu
Ce→B
Mg→Ba
Be→Cu
Cr→S
Os→Te
Au→As
Os→Se
Zr→U
Zr→Th
Zr→Sn
Zr→Pt
Zr→Si
Th→U
Zr→Te
Th→Sn
Au→P

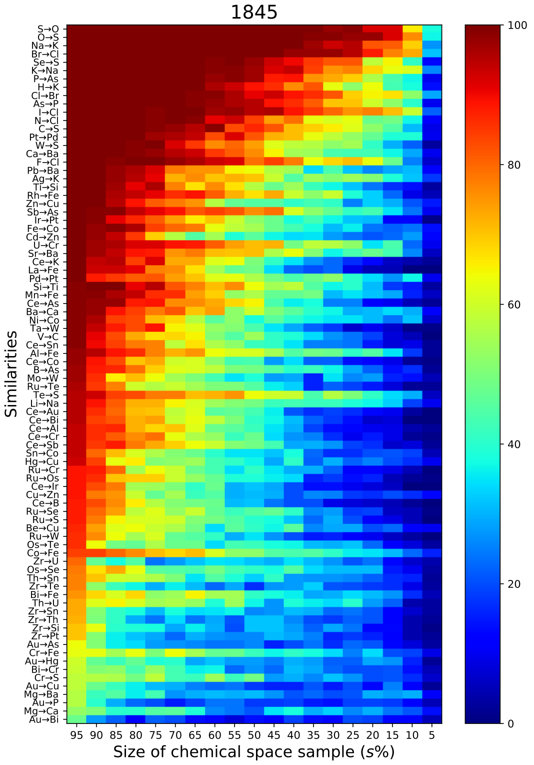
1845
Similarities
Size of chemical space sample (s%)
95 90 85 80 75 70 65 60 55 50 45 40 35 30 25 20 15 10 5
0 20 40 60 80 100

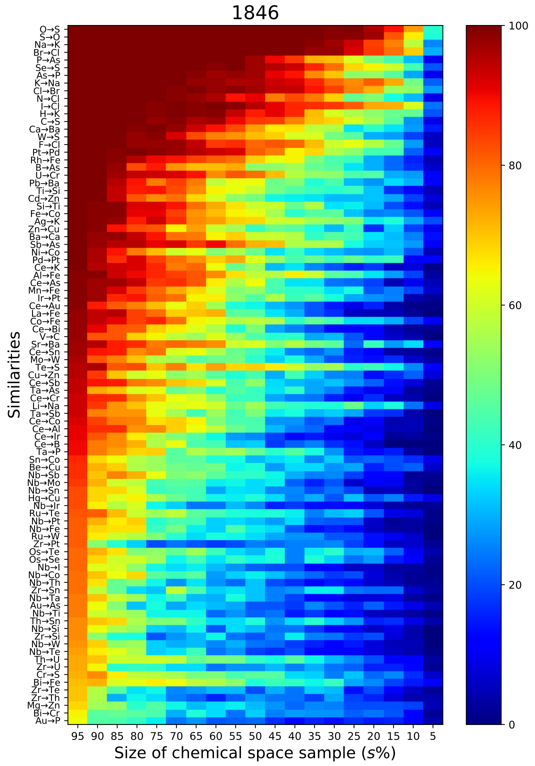
1846
Similarities
Size of chemical space sample (s%)
95 90 85 80 75 70 65 60 55 50 45 40 35 30 25 20 15 10 5
0 20 40 60 80 100

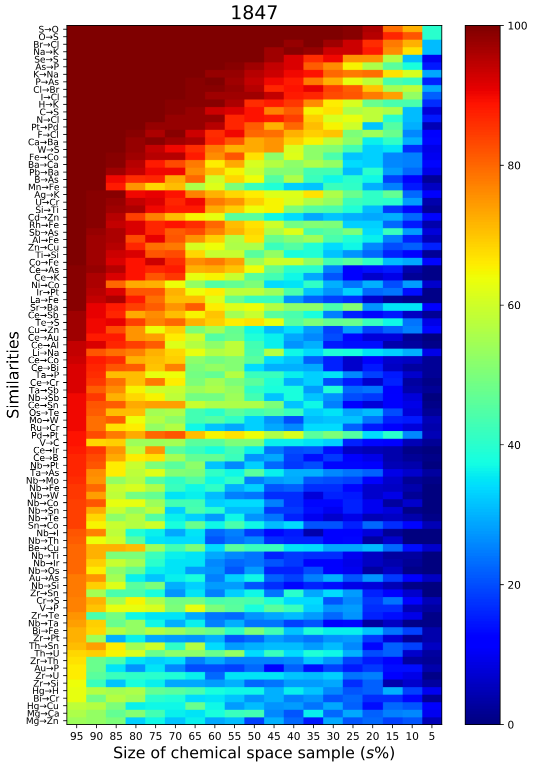
1847
Similarities
Size of chemical space sample (s%)
95 90 85 80 75 70 65 60 55 50 45 40 35 30 25 20 15 10 5
0 20 40 60 80 100

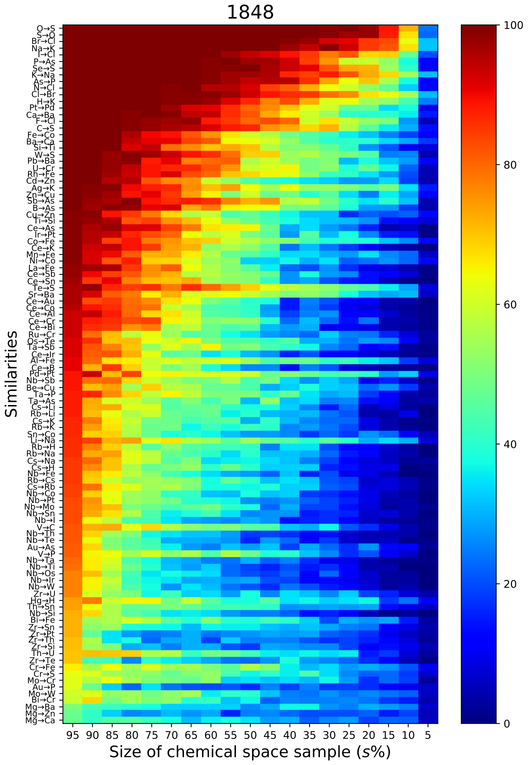
1848
Similarities
Size of chemical space sample (s%)
95 90 85 80 75 70 65 60 55 50 45 40 35 30 25 20 15 10 5
0 20 40 60 80 100

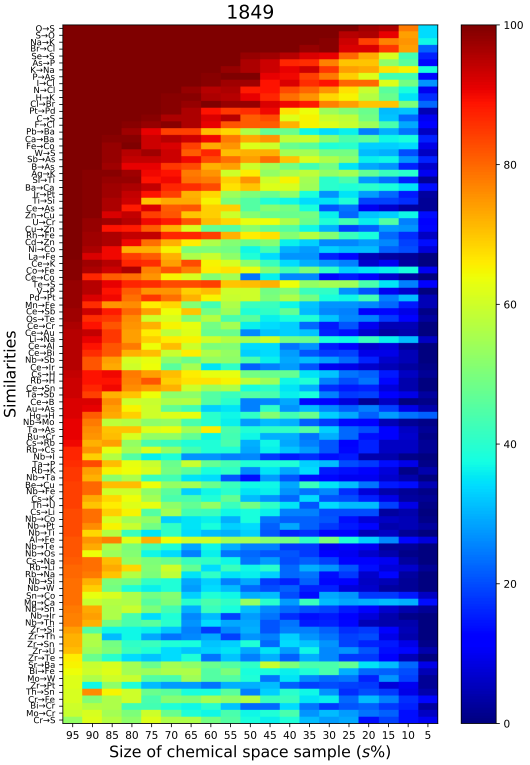
1849
Similarities
Size of chemical space sample (s%)
95 90 85 80 75 70 65 60 55 50 45 40 35 30 25 20 15 10 5
0 20 40 60 80 100

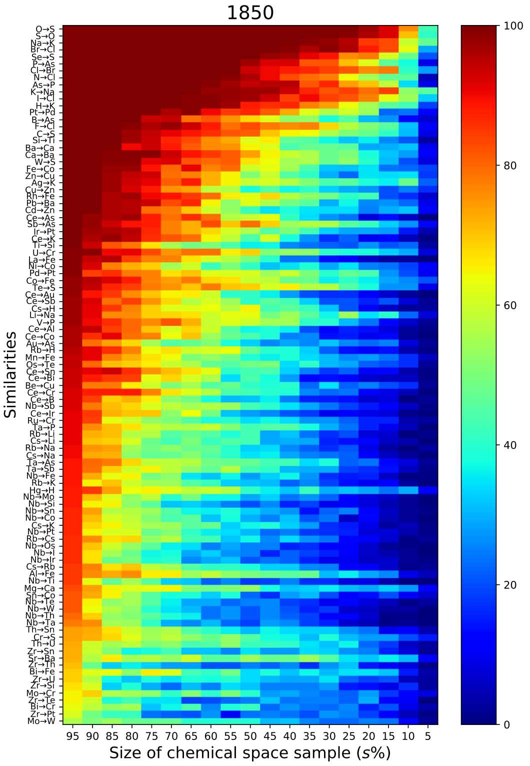
1850
Similarities
Size of chemical space sample (s%)
95 90 85 80 75 70 65 60 55 50 45 40 35 30 25 20 15 10 5
0 20 40 60 80 100

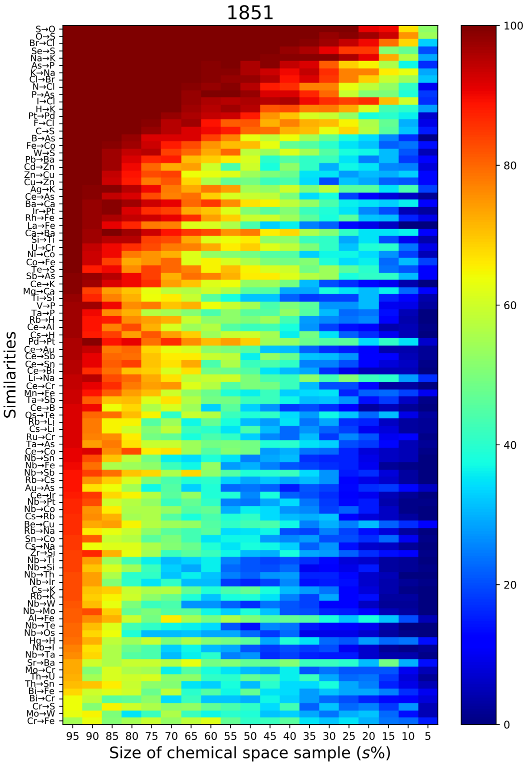
1851
Similarities
Size of chemical space sample (s%)
95 90 85 80 75 70 65 60 55 50 45 40 35 30 25 20 15 10 5
0 20 40 60 80 100

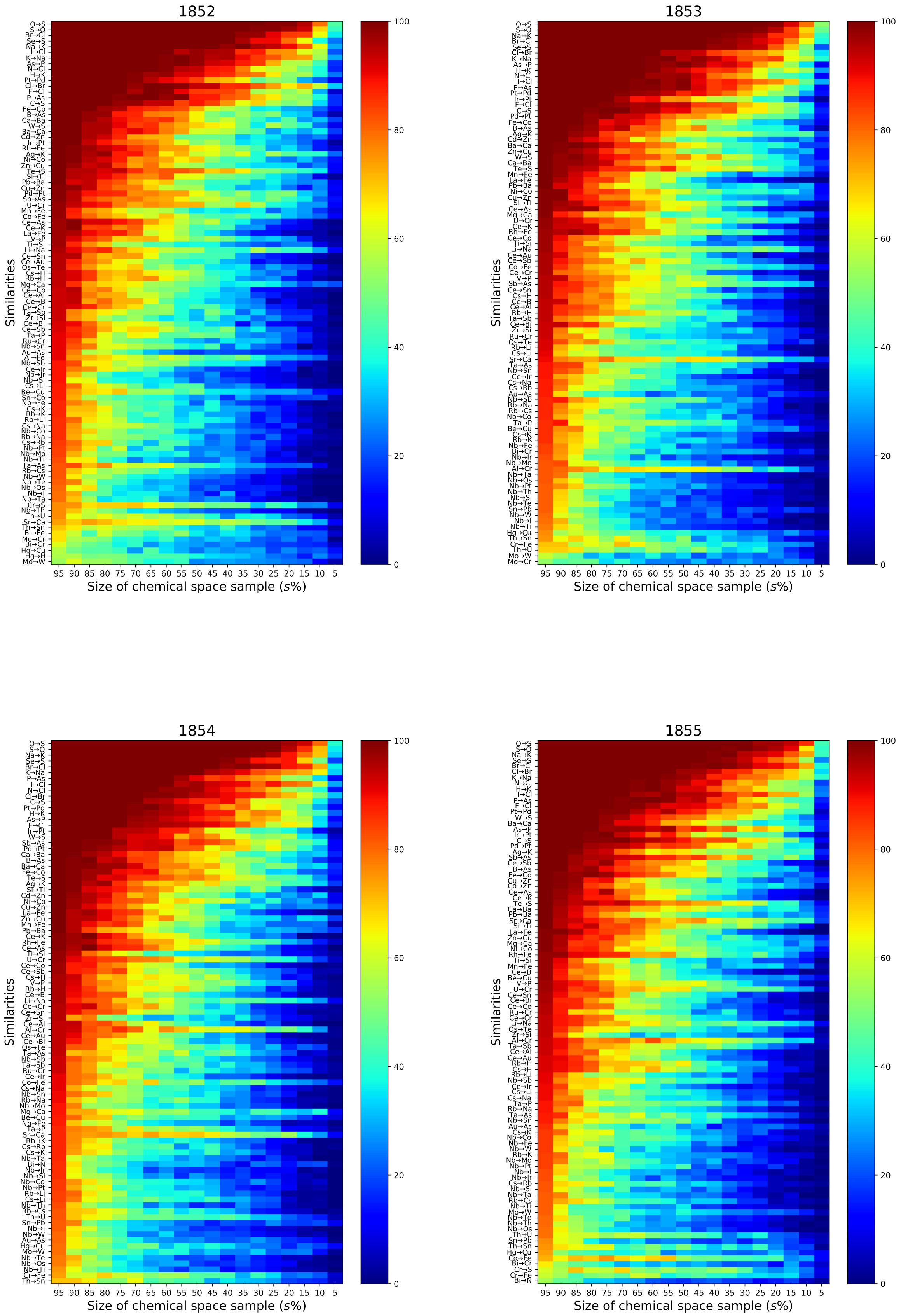
1852
1853
1854
1855
Similarities
Size of chemical space sample (s%)
95 90 85 80 75 70 65 60 55 50 45 40 35 30 25 20 15 10 5
0
20
40
60
80
100

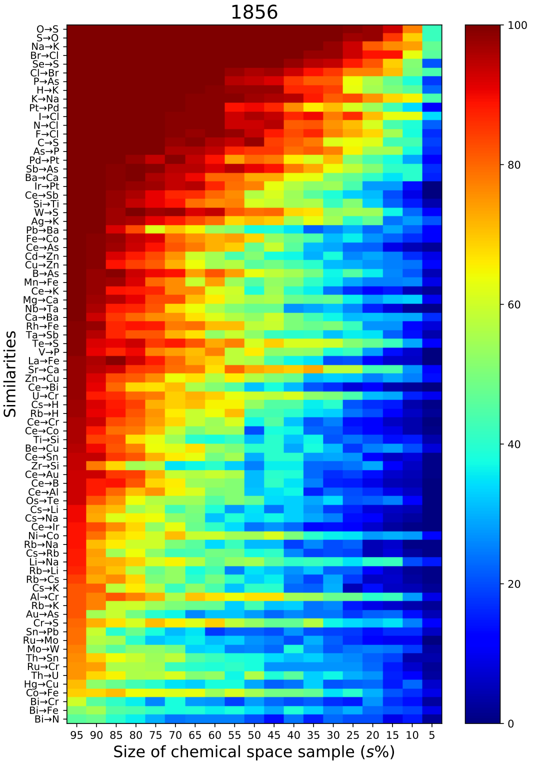
1856
Similarities
Size of chemical space sample (s%)
95 90 85 80 75 70 65 60 55 50 45 40 35 30 25 20 15 10 5
0 20 40 60 80 100

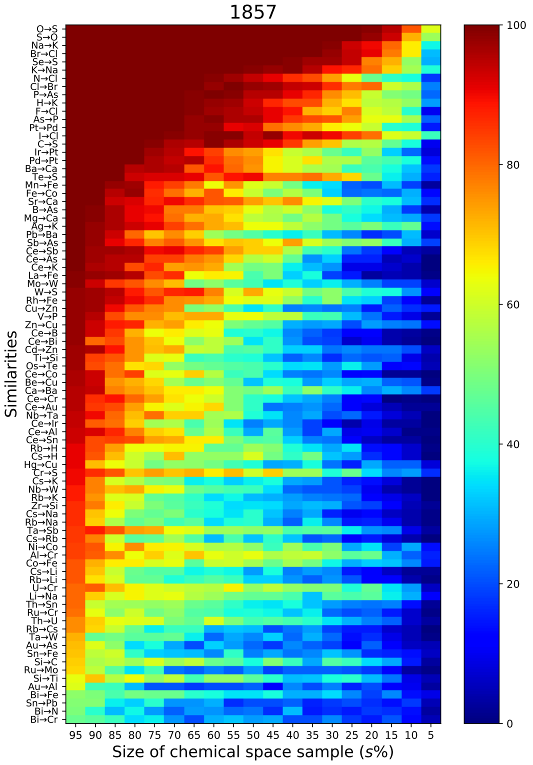
1857
Similarities
Size of chemical space sample (s%)
95 90 85 80 75 70 65 60 55 50 45 40 35 30 25 20 15 10 5
0 20 40 60 80 100

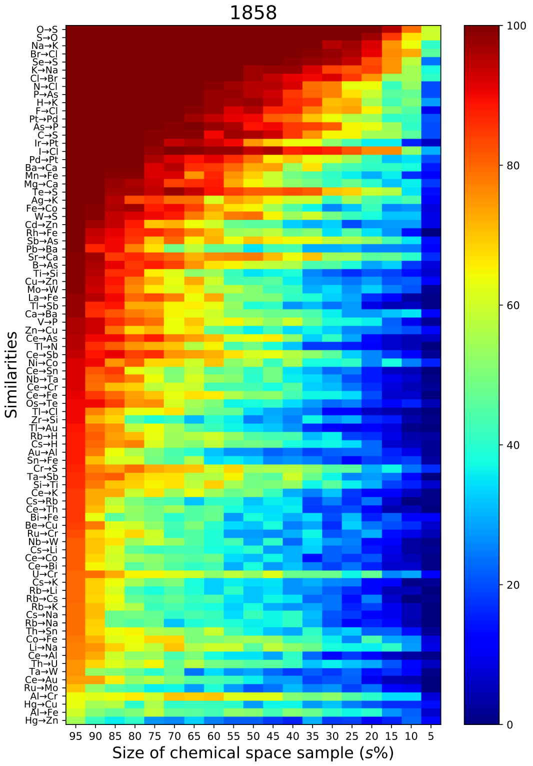
1858
Similarities
Size of chemical space sample (s%)
95 90 85 80 75 70 65 60 55 50 45 40 35 30 25 20 15 10 5
0 20 40 60 80 100

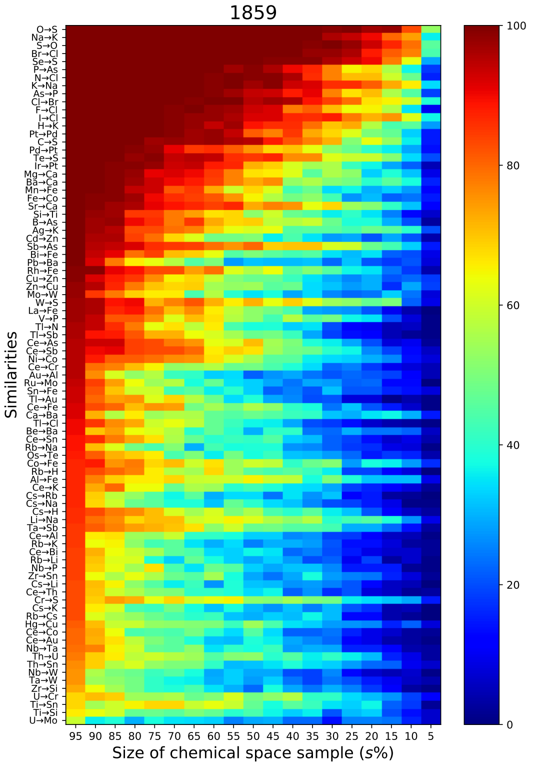
1859
Similarities
Size of chemical space sample (s%)
95 90 85 80 75 70 65 60 55 50 45 40 35 30 25 20 15 10 5
0 20 40 60 80 100

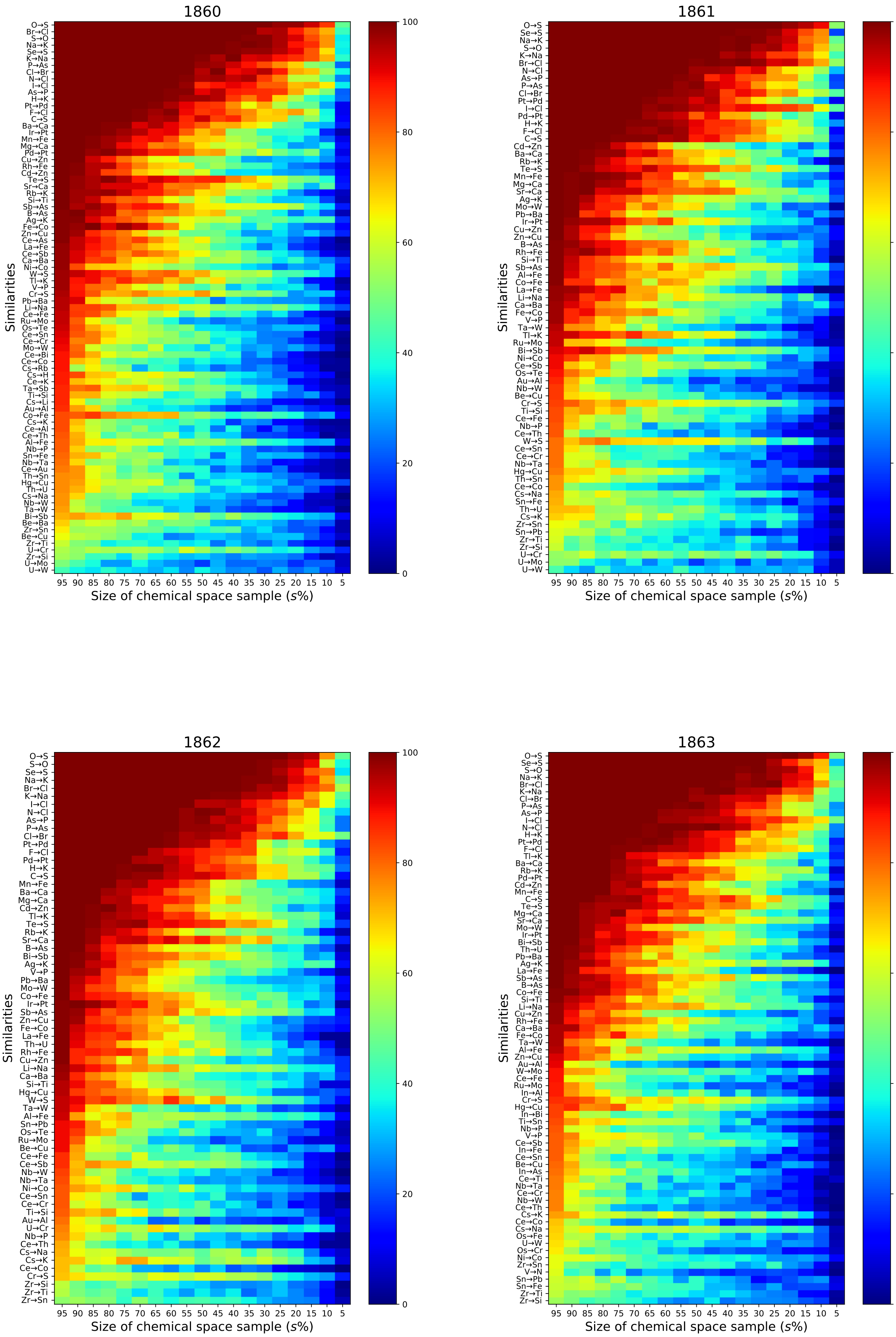
1860
1861
1862
1863
Similarities
Size of chemical space sample (s%)
95 90 85 80 75 70 65 60 55 50 45 40 35 30 25 20 15 10 5
0
20
40
60
80
100

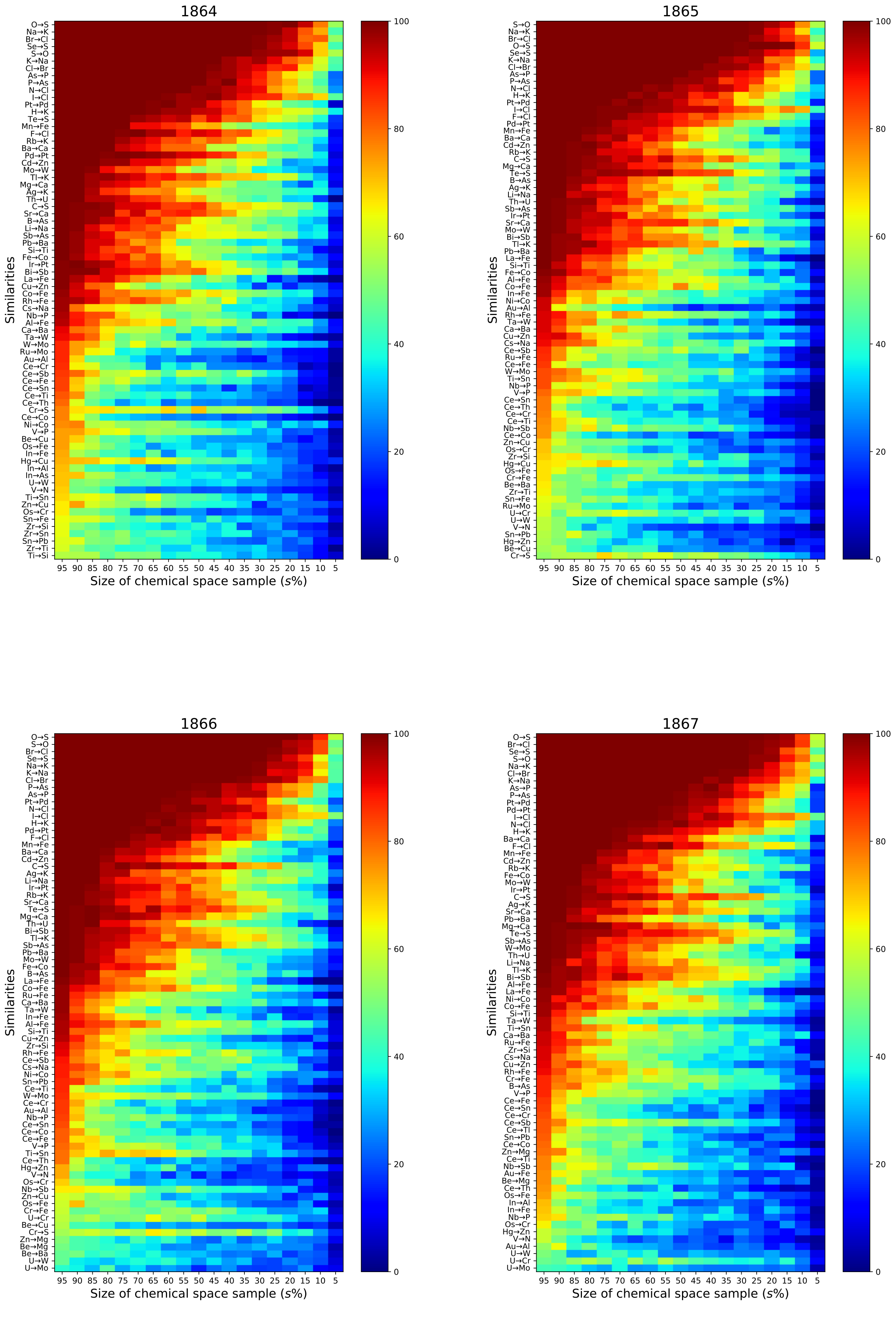
1864
1865
1866
1867
Similarities
Size of chemical space sample (s%)
95 90 85 80 75 70 65 60 55 50 45 40 35 30 25 20 15 10 5
0 20 40 60 80 100

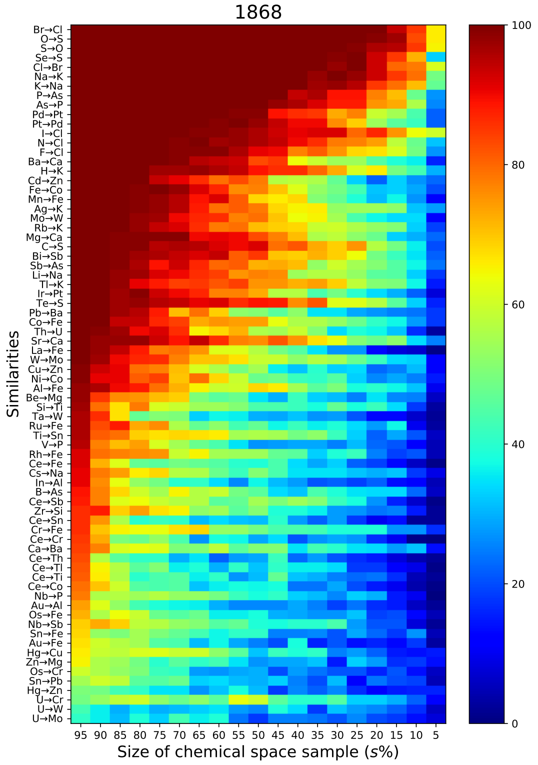

Figure S9: Stability of similarities regarding chemical space size. Each row contains a given similarity observed by considering the chemical space in year *y*. The stability of each similarity corresponds to the percentage of appearance of such similarity in the sampled space of size *s*%. Colours associated to this percentage are shown on the right bar. Further details in Materials and Methods (main document).

11 Contrasting Meyer and Mendeleev' systems of chemical elements with those of the chemical space (presentist approach)

We took the three systems by Meyer, which were formulated in 1864 (6), 1868 (7) and 1869/70 (4); and the first Mendeleev' system published in 1869 (1). We extracted the similarities among the elements out of these systems and contrasted them with the "most similar" relationships of the systems of elements of the respective years 1863, 1867 and 1868. The time difference of one year between the system of elements of each author and the system of elements of the chemical space is to regard the time required for a chemist to be updated with the literature in the nineteenth-century.

As Meyer did not explicitly discuss similarities among elements, we followed his principle of regarding as similar those elements belonging to a column of his 1864 and 1868 tables. In addition, we regarded elements with the same valency as similar for his 1864 table, plus the well-known similarities among transition metals Mn=Fe, Ni, Co, Ru, Rh, Pd and Pt, Ir, Os. Hence, for instance, we regarded Li, Na, K, Rb, Cs, Tl and Cu, Ag, Ag as similar, as they hold valence 1. Note that Meyer did not write the valency of Cu, Ag and Ag in 1864, but one can infer it was 1 from the groups on the left of this group of elements (6). For Meyer's periodic system of 1868, besides elements belonging in the same column, we consider the following transition metal similarities observed in his table: Mn, Fe, Co, Ru, Rh, Pd and Pt, Ir, Os. In this case, we did not regard same valency as a similarity criterion, because valency is not highlighted in Meyer's table and because of his shifting and reshifting of Al (7, 9). Similarities in his 1869 table are further discussed in the paper where they were published. Therefore, besides the usual vertical similarities (in the 1869 published representation corresponding to rows), we also included those similarities mentioned by Meyer (4), plus the transition metal ones: Mn, Ru, Os, Fe, Rh, Ir and Co=Ni, Pd, Pt (Figure S7). Mendeleev discussed thoroughly the similarities and even some lack of similarities (8), both of them listed in Table S2.

By contrasting the similarities reported by each author with those corresponding to most similar relationships among chemical elements allowed by the chemical space, we calculated the true positive and false negative rates of each system of elements. True positives (TP) correspond to similarities observed by year y and extracted from chemist's system of

elements. True negatives (TN) correspond to non most similar relationships observed in year y that are also non similarities according to chemist's system of elements. False positives (FP) are non similarities in year y corresponding to similarities in chemist's system of elements. False negatives (FN) are similarities in year y not retrieved from chemist's system of elements. The true positive rate (TPR) is given by $TP/(TP + FN)$, false positive rate (FPR) by $FP/(FP + TN)$.

Table S3: True positives and negatives (TP, TN), false positives and negatives (FP, FN), true positive and false positive rates (TPR, FPR) and the data used for their calculation.

	Meyer 1864	Meyer 1868	Meyer 1869/70	Mendeleev 1869
Number of elements (n) in chemist's system of elements	50	52	55	60
Possible number of similarities for n elements: $n \times (n - 1)$	2450	2652	2970	3540
Number of most similar relationships observed from the chemical space in year $y - 1$, with y the publication year of chemist's system of elements	77	74	74	74
Number of similarities retrieved from chemist's system of elements	586	204	222	403
Number of most similar relationships observed from the chemical space and not retrieved from chemist's system of elements	36	35	28	31
Number of most similar relationships observed from the chemical space and from chemist's system of elements	41	39	46	43
Number of similarities retrieved from chemist's system of elements but not corresponding to most similar relationships from the chemical space in year $y - 1$	545	165	176	360
True positives (TP)	0.532	0.527	0.622	0.581
True negatives (TN)	0.77	0.907	0.939	0.896
False positives (FP)	0.23	0.064	0.061	0.104
False negatives (FN)	0.468	0.473	0.378	0.419
True positive rate (TPR)	0.532	0.527	0.622	0.581
False positive rate (FPR)	0.23	0.066	0.061	0.104

12 Evolution of the system of chemical elements (retrodictive approach)

Table S4: Atomic weights as reported by nine nineteenth-century chemists. The primary sources for these figures are: Dalton (10), pages 546, 547; Thomson (11); Berzelius 1819 (12); Berzelius 1826 (13, 14, 15); Gmelin (16), pages 50, 51; Lenßen (17), pages 122, 123 (we assume 1 for H); Meyer 1864 (6), we assume 1 for H; Odling (18), page 642; Hinrichs (19), pages 13, 14; Meyer 1868 (7, 9); Mendeleev (1); Meyer 1869/70 (4), current (20).

Element	Dalton 1810	Thomson 1813	Berzelius 1819	Berzelius 1826	Gmelin 1843	Lenßen 1857	Meyer 1864	Odling 1864	Hinrichs 1867	Meyer 1868	Mendeleev 1869	Meyer 1869/70	Current
H	1	0.132	6.2175	6.244	1	1	1	1	1	1	1	1	1.008
O	7	1	100	100	8	8	16	16	16	16	16	15.96	15.999
C	5.4	0.751	75.33	75	6	6	12	12	12	12	12	11.97	12.011
N	5	0.878	77.26	88.518	14	7	14.04	14	14	14.04	14	14.01	14.007
S	13	2	201.165	207.58	16	16	32.07	32	32	32.07	32	31.98	32.06
P	9	1.32	392.3	196.15	31.4	16	31	31	31	31	31	30.9	30.973
Cl	33	4.498	142.65	220	35.4	17.7	35.46	35.5	35.5	35.46	35.5	35.38	35.45
Na	28	5.882	581.84	290.92	23.2	23	23.05	23	23	23.05	23	22.99	22.989
K	42	5	979.83	487.915	39.2	39.11	39.13	39	39.1	39.13	39	39.04	39.0983
Ca	24	2.62	512.06	256.03	20.5	20	40	40	40	40	40	39.9	40.078
Mg	17	1.368	316.72	158.36	12.7	12	24	24	24	24	24	23.9	24.305
Sr	46	5.9	1094.6	547.3	44	43.67	87.6	87.5	87.6	87.6	87.6	87	87.62
Ba	68	8.731	1713.86	856.93	68.6	68.59	137.1	137	137	137.1	137	136.8	137.327
Fe	50	6.666	678.43	339.215	27.2	28	56	56	56	56	56	55.9	55.845
Cu	56	8	791.39	395.695	31.8	31.7	63.5	63.5	63.4	63.5	63.4	63.3	63.546
Zn	56	4.315	806.45	403.225	32.2	59	65	65	65.2	65	65.2	64.9	65.38
Ag	100	12.618	2703.21	1351.605	108.1	108	107.94	108	108	107.94	108	107.66	107.8682
Hg	167	25	2531.6	1265.8	101.4	100	200.2	200	200	200.2	200	199.8	200.592
Pb	95	25.974	2589	1294.5	103.8	103.6	207	207	207	207	207	206.4	207.2
Li			255.63	127.8	68.6	6.95	7.03	7	7	7.03	7	7.01	6.94
Be	30	3.6	662.56	331.28	17.7	7	9.3	9	9.3	9.3	9.4	9.3	9.012
B			69.655	135.98	10.8	11		11	11		11	11	10.81
F			75.03	116.9	18.7	9.5	19	19	19	19	19	19.1	18.998
Al	15	2.136	342.333	171.667	13.7	13.7		27.5	27.4	27.3	27.4	27.3	26.981
Si	45	4.066	296.42	277.8	14.8	15	28.5	28	28	28.5	28	28	28.085
Ti	40			389.1	24.5	25	48	50	50	48	50	48	47.867
Cr			703.638	351.86	28.1	26.8		52.5	52.2	52.6	52	52.4	51.9961
Mn	40	7.13	711.575	355.787	27.6	27.5	55.1	55	55	55.1	55	54.8	54.938
Co	55	7.326	738	369	29.6	29.5	58.7	59	60	58.7	59	58.6	58.933
Ni	50	7.305	739.51	369.755	29.6	29.6	58.7	59	58	58.7	59	58.6	58.6934
As	42	6	940.77	470.385	75.2	37.5	75	75	75	75	75	74.9	74.921
Se			495.91	494.59	40	39.7	78.8	79.5	79.4	78.8	79.4	78	78.971
Br					78.4	40	79.97	80	80	79.97	80	79.75	79.904
Rb							85.4	85	85.4	85.4	85.4	85.2	85.4678
Zr	45	5.656		420.21	22.4	33.6	90	89.5	89.6	90	90	89.7	91.224
Ce	45	11.494	1149.44	574.72	46.3	47.3		92			92		140.116
Mo		5.882	596.8	598.56	48	46	92	96	92	92	96	95.6	95.95
Rh			1500.1	750.65	52.1	51.2	104.3	104	104.4	104.3	104.4	104.1	102.905
Ru						52.1	104.3	104		104.3	104.4	103.5	101.07
Pd		14.204	1407.5	714.6	53.4	53.2	106	106.5	106.6	106	106.6	106.2	106.42
Cd			1393.54	696.77	55.8	20	111.9	112	112	111.9	112	111.6	112.414
U	60	12	3146.86	2711.36	217	60		120	120		116		238.028
Sn	50	14.705	1470.58	735.29	59		117.6	118	118	117.6	118	117.8	118.71
Sb	40		1612.9	806.45	129	60	120.6	122	122	120.6	122	122.1	121.76
Te		4.107	806.45	403.225	64	64.2	128.3	129	128	128.3	128	128	127.6
I				783.35	126	63.5	126.8	127	127	126.8	127	126.5	126.904
Cs							133	133	133	133	133	132.7	132.905
Tl							204	203	204	204	204	202.7	204.38
Bi	68	11.111	1773.8	1330.4	106.4	104	208	210	210	208	210	207.5	208.98
Th					59.6	59.5		231.5	231		118		232.0377
In									71		75.6	113.4	114.818
V					68.6	68.5	137	137	139.2	137	51	51.2	50.9415
La					36.1	47		92			94		138.905
Nb											94	93.7	92.906
Ta			1823.15	1152.87	185	92.3	137.6	138	137.6	137.6	182	182.2	180.947
W	56	8	1207.689	1183.2	95	92	184	184	184	184	186	183.5	183.84
Pt	100	12.161	1215.226	1215.23	98.7	99	197.1	197	198	197.1	197.4	196.7	195.084
Ir					98.7		197.1	197	198	197.1	198	196.7	192.217
Os					99.6	99.4	199	199		199	199	198.6	190.23
Au	140		2486	1243	199	98.4	196.7	196.5	197	196.7	197	196.2	196.966

Dalton 1810

**H**																
Li	**Be**											B	**C**	**N**	**O**	F
**Na**	**Mg**											**Al**	Si	**P**	**S**	**Cl**
**K**	**Ca**		**Ti**	V	Cr	Mn	**Fe**	**Co**	**Ni**	**Cu**	**Zn**			**As**	Se	Br
Rb	**Sr**		Zr	Nb	Mo		Ru	Rh	Pd	**Ag**	Cd	In	**Sn**	**Sb**	Te	I
Cs	**Ba**			Ta	W		Os	Ir	**Pt**	**Au**	**Hg**	Tl	**Pb**	Bi		
		La	Ce													
			Th		**U**											

Thomson 1813

**H**																
Li	**Be**											B	**C**	**N**	**O**	F
**Na**	**Mg**											**Al**	**Si**	**P**	**S**	**Cl**
**K**	**Ca**		Ti	V	Cr	Mn	**Fe**	**Co**	**Ni**	**Cu**	**Zn**			**As**	Se	Br
Rb	**Sr**		Zr	Nb	Mo		Ru	Rh	**Pd**	**Ag**	Cd	In	**Sn**	Sb	**Te**	I
Cs	**Ba**			Ta	**W**		Os	Ir	**Pt**	Au	**Hg**	Tl	**Pb**	**Bi**		
		La	Ce													
			Th		**U**											

Berzelius 1819

**H**																
**Li**	**Be**											**B**	**C**	**N**	**O**	**F**
**Na**	**Mg**											**Al**	**Si**	**P**	**S**	**Cl**
**K**	**Ca**		Ti	V	**Cr**	**Mn**	**Fe**	**Co**	**Ni**	**Cu**	**Zn**			**As**	**Se**	Br
Rb	**Sr**		Zr	Nb	Mo		Ru	**Rh**	**Pd**	**Ag**	**Cd**	In	**Sn**	**Sb**	**Te**	I
Cs	**Ba**			**Ta**	**W**		Os	Ir	**Pt**	**Au**	**Hg**	Tl	**Pb**	**Bi**		
		La	**Ce**													
			Th		**U**											

Berzelius 1826

H																
Li	Be											B	C	N	O	F
Na	Mg											Al	Si	P	S	Cl
K	Ca		Ti	V	Cr	Mn	Fe	Co	Ni	Cu	Zn			As	Se	Br
Rb	Sr		Zr	Nb	Mo		Ru	Rh	Pd	Ag	Cd	In	Sn	Sb	Te	I
Cs	Ba			Ta	W		Os	Ir	Pt	Au	Hg	Tl	Pb	Bi		
		La	Ce													
			Th		U											

Gmelin 1843

H																
Li	Be											B	C	N	O	F
Na	Mg											Al	Si	P	S	Cl
K	Ca		Ti	V	Cr	Mn	Fe	Co	Ni	Cu	Zn			As	Se	Br
Rb	Sr		Zr	Nb	Mo		Ru	Rh	Pd	Ag	Cd	In	Sn	Sb	Te	I
Cs	Ba			Ta	W		Os	Ir	Pt	Au	Hg	Tl	Pb	Bi		
		La	Ce													
			Th		U											

Lenßen 1857

H																
Li	Be											B	C	N	O	F
Na	Mg											Al	Si	P	S	Cl
K	Ca		Ti	V	Cr	Mn	Fe	Co	Ni	Cu	Zn			As	Se	Br
Rb	Sr		Zr	Nb	Mo		Ru	Rh	Pd	Ag	Cd	In	Sn	Sb	Te	I
Cs	Ba			Ta	W		Os	Ir	Pt	Au	Hg	Tl	Pb	Bi		
		La	Ce													
			Th		U											

Meyer 1864

H																
Li	Be											B	C	N	O	F
Na	Mg											Al	Si	P	S	Cl
K	Ca		Ti	V	Cr	Mn	Fe	Co	Ni	Cu	Zn			As	Se	Br
Rb	Sr		Zr	Nb	Mo		Ru	Rh	Pd	Ag	Cd	In	Sn	Sb	Te	I
Cs	Ba			Ta	W		Os	Ir	Pt	Au	Hg	Tl	Pb	Bi		
		La	Ce													
			Th		U											

Odling 1864

H																
Li	Be											B	C	N	O	F
Na	Mg											Al	Si	P	S	Cl
K	Ca		Ti	V	Cr	Mn	Fe	Co	Ni	Cu	Zn			As	Se	Br
Rb	Sr		Zr	Nb	Mo		Ru	Rh	Pd	Ag	Cd	In	Sn	Sb	Te	I
Cs	Ba			Ta	W		Os	Ir	Pt	Au	Hg	Tl	Pb	Bi		
		La	Ce													
			Th		U											

Hinrichs 1867

H																
Li	Be											B	C	N	O	F
Na	Mg											Al	Si	P	S	Cl
K	Ca		Ti	V	Cr	Mn	Fe	Co	Ni	Cu	Zn			As	Se	Br
Rb	Sr		Zr	Nb	Mo		Ru	Rh	Pd	Ag	Cd	In	Sn	Sb	Te	I
Cs	Ba			Ta	W		Os	Ir	Pt	Au	Hg	Tl	Pb	Bi		
		La	Ce													
			Th		U											

Meyer 1868

**H**																
**Li**	**Be**											B	**C**	**N**	**O**	**F**
**Na**	**Mg**											**Al**	**Si**	**P**	**S**	**Cl**
**K**	**Ca**		**Ti**	**V**	**Cr**	**Mn**	**Fe**	**Co**	**Ni**	**Cu**	**Zn**			**As**	**Se**	**Br**
**Rb**	**Sr**		**Zr**	Nb	**Mo**		**Ru**	**Rh**	**Pd**	**Ag**	**Cd**	In	**Sn**	**Sb**	**Te**	**I**
**Cs**	**Ba**			**Ta**	**W**		**Os**	**Ir**	**Pt**	**Au**	**Hg**	**Tl**	**Pb**	**Bi**		
		La	Ce													
			Th		U											

Mendeleev 1869

H																
Li	Be											B	C	N	O	F
Na	Mg											Al	Si	P	S	Cl
K	Ca		Ti	V	Cr	Mn	Fe	Co	Ni	Cu	Zn			As	Se	Br
Rb	Sr		Zr	Nb	Mo		Ru	Rh	Pd	Ag	Cd	In	Sn	Sb	Te	I
Cs	Ba			Ta	W		Os	Ir	Pt	Au	Hg	Tl	Pb	Bi		
		La	Ce													
			Th		U											

Meyer 1869/1870

**H**																
**Li**	**Be**											**B**	**C**	**N**	**O**	**F**
**Na**	**Mg**											**Al**	**Si**	**P**	**S**	**Cl**
**K**	**Ca**		**Ti**	**V**	**Cr**	**Mn**	**Fe**	**Co**	**Ni**	**Cu**	**Zn**			**As**	**Se**	**Br**
**Rb**	**Sr**		**Zr**	**Nb**	**Mo**		**Ru**	**Rh**	**Pd**	**Ag**	**Cd**	**In**	**Sn**	**Sb**	**Te**	**I**
**Cs**	**Ba**			**Ta**	**W**		**Os**	**Ir**	**Pt**	**Au**	**Hg**	**Tl**	**Pb**	**Bi**		
		La	Ce													
			Th		U											

Figure S10: Chemical elements used to build up the systems of elements of the nine nineteenth-century chemists. Elements known by the year discussed in each table are shown in black, while undiscovered elements and known by 1869 in grey. In red mixtures that were thought to be elements.

Table S5: Selection of elements for each chemist. The number of initial elements refers to those we used to build up the systems of elements of each chemist (Supplementary Figure S10). Disregarded elements correspond to those not having substances in the database participating in single step chemical reactions (Section 1).

Chemist	Number of initial elements	Number of disregarded elements
Dalton (1810)	37	6[e]
Thomson (1813)	37	4[f]
Berzelius (1819)	46	1[g]
Berzelius (1826)	49	0
Gmelin (1843)	54	1[h]
Lenßen (1857)[a]	53	0
Meyer (1864)[a]	51[b]	0
Odling (1864)	58	0
Hinrichs (1867)	55	0
Meyer (1868)	53[c]	0
Mendeleev (1869)	60	0
Meyer (1869/70)	56[d]	0

[a] We assumed 1 as the atomic weight for H.

[b] Meyer reported 50 elements (Figure S10), but it is clear that H made part of his system (9).

[c] Meyer reported 52 elements, here we included H (see note b).

[d] Meyer reported 55 elements, here we included H (see note b).

[e] Bi, Ce, Mn, Si, W, Zr

[f] Ce, Mn, Mo, Zr

[g] Mo

[h] La

Table S6: Spearman rank correlation for the ordering of elements according to atomic weights by each of the nine nineteenth-century chemists.

Element	Dalton 1810	Thomson 1813	Berzelius 1819	Berzelius 1826	Gmelin 1843	Lenßen 1857	Meyer 1864	Odling 1864	Hinrichs 1867	Meyer 1868	Mendeleev 1869	Meyer 1869/70	Current
Dalton 1810	1	0.9050	0.8750	0.8907	0.8037	0.9008	0.8779	0.8786	0.8849	0.8817	0.8771	0.8817	0.8711
Thomson 1813	0.9050	1	0.9037	0.8932	0.8322	0.8516	0.8389	0.8430	0.8444	0.8447	0.8440	0.8447	0.8518
Berzelius 1826	0.8750	0.9037	1	0.9561	0.8747	0.9033	0.8903	0.8968	0.8929	0.8938	0.8954	0.8975	0.9047
Berzelius 1826	0.8907	0.8932	0.9561	1	0.8886	0.9436	0.9409	0.9393	0.9370	0.9434	0.9378	0.9437	0.9440
Gmelin 1843	0.8037	0.8322	0.8747	0.8886	1	0.8626	0.8545	0.8512	0.8439	0.8577	0.8469	0.8488	0.8321
Lenßen 1857	0.9008	0.8516	0.9033	0.9436	0.8626	1	0.9454	0.9437	0.9414	0.9478	0.9277	0.9280	0.9056
Meyer 1864	0.8779	0.8389	0.8903	0.9409	0.8545	0.9454	1	1	0.9997	1	0.9750	0.9750	0.9741
Odling 1864	0.8786	0.8430	0.8968	0.9393	0.8512	0.9437	1	1	0.9998	1	0.9676	0.9762	0.9552
Hinrichs 1867	0.8849	0.8444	0.8929	0.9370	0.8439	0.9414	0.9997	0.9998	1	0.9997	0.9639	0.9664	0.9584
Meyer 1868	0.8817	0.8447	0.8938	0.9434	0.8577	0.9478	1	1	0.9997	1	0.9758	0.9758	0.9750
Mendeleev 1869	0.8771	0.8440	0.8954	0.9378	0.8469	0.9277	0.9750	0.9676	0.9639	0.9758	1	0.9946	0.9669
Meyer 1869/70	0.8817	0.8447	0.8975	0.9437	0.8488	0.9280	0.9750	0.9762	0.9664	0.9758	0.9946	1	0.9994
Current	0.8711	0.8518	0.9047	0.9440	0.8321	0.9056	0.9741	0.9552	0.9584	0.9750	0.9669	0.9994	1

13 Approximating formulae according to the set of atomic weights of a given chemist

We exemplify our algorithm by taking contemporary compound Fe_2O_3, whose formula is to be approximated according to the set of atomic weights of Berzelius by 1819 (Table S4). Hence, it is our aim to find $f(\text{Fe})$ and $f(\text{O})$ in $\text{Fe}_{2\times f(\text{Fe})}\text{O}_{3\times f(\text{O})}$, according to 1819 Berzelius' weights. We start by collecting the contemporary atomic weights W of the elements involved in the formula, as well as those A reported by the author (chemist) (12). Corresponding weights for H are also needed for the sake of normalisation:

$$W(\text{Fe}) = 55.845 \quad A(\text{Fe}) = 678.43$$
$$W(\text{H}) = 1.008 \quad A(\text{H}) = 6.2175$$

(W/A)(Fe) = (55.845/1.008)/(678.43/6.2175) = 0.5077

(A/W)(Fe) = (678.43/6.2175)/(55.845/1.008) = 1.9695

So, the ratios we are interested in approximating as simple ratios are either 0.05077 or 1.9695. We say that 0.5077 must be expressed by any fraction f of the form x/y, such that $0 < f \leq 1$. Likewise, that 1.9695 can be decomposed and approximated by a fraction of the form $1.9695 = 1 + x/y$. This corresponds to the fractions of a Farey sequence. For example, the fractions of a Farey sequence of order 3 are $F_3 = \{0/1, 1/3, 1/2, 2/3, 1/1\}$, which are all those reduced fractions x/y with x and y lower or equal than 3 (21).

For our purposes, we need to find the fraction of a Farey sequence of order n that best approximates 0.5077 or 1.9695 (i.e. 0.9695). This procedure entails selecting the order n of the Farey sequence and a way to quantify how a fraction of F_n approximates 0.5077 or 0.9695. To select n we took all formulae of the chemical space by 1869 and extracted all their stoichiometric coefficients. We found that the maximum value was lower than 200. This means that actual stoichiometric coefficients of the chemical space are at most fractions of 200, including 200. So, all fractions to be explored in the current work correspond to the Farey sequence F_{200}.

Having selected the order of the Farey sequence to work with, we then proceed to devise a way to quantify the accuracy of the approximation of the ratio r by the fraction f. We, therefore, calculate the relative error of the approximation.

$$error(r, f) = \frac{|r - f|}{r} \times 100$$

Where $f \in F_{200}$.

As F_{200} has 12,233 fractions (the number of fractions $|F_n| = \frac{n(n+3)}{2} - \sum_{k=2}^{n} |F_{\lfloor \frac{n}{k} \rfloor}|$ (22), we set up an order to explore those fractions, based on the aim of finding simple fractions. That is, we need fractions x/y such that both x and y are small whole numbers. We quantify such a "simplicity" of fractions by their associated "area" $x \times y$. The smaller the area of a fraction, the simpler the fraction is. Hence, we order F_{200} fractions by non decreasing order of their area, that is F_{200} is arranged as (0/1, 1/1,1/2, 2/1, 1/3, 3/1,..., 199/200, 200/199). According to this order, we quantify the relative error of the approximation. To decide which fraction better

approximates the ratio in study (in this example either 0.5077 or 0.9695), we further need a stopping criterion indicating the amount of error to be allowed (tolerance). We selected 20 different values of tolerance τ, from 1% to 20% of relative error.

Hence, the best fraction approximating the given ratio is that simple fraction whose $error(r, f) \leq \tau$. For each τ we have a best approximating fraction.

Let us start with $\tau = 1$.

$$error(0.5077, 0/1) = 100 \nleq 1$$

$$error(0.9695, 0/1) = 100 \nleq 1$$

$$error(0.5077, 1/1) = 96.95 \nleq 1$$

$$error(0.9695, 1/1) = 3.14 \nleq 1$$

$$\vdots$$

$$error(0.5077, 40/78) = 1.00 \leq 1$$

$$error(0.9695, 40/78) = 47.11 \nleq 1$$

So, we say that 0.5077 is better approximated by 40/78 with a tolerance of less than 1% of relative error. That is, that by approximating 0.5077 by 40/78 we are reducing 0.5077 1% of its value.

This result implies that contemporary Fe_2O_3 is transformed into $Fe_{2\times40/78}O_?$, according to the table of atomic weights of Berzelius by 1819. We now apply the above procedure to find the factor to multiply the stoichiometric coefficient of oxygen in Fe_2O_3.

$$W(\mathrm{O}) = 15.999 \quad A(\mathrm{O}) = 100.00$$
$$W(\mathrm{H}) = 1.008 \quad A(\mathrm{H}) = 6.2175$$

$(W/A)(\mathrm{O}) = (15.999/1.008)/(100.00/6.2175) = 0.9868$

$(A/W)(\mathrm{O}) = (100.00/6.2175)/(15.999/1.008) = 1.0133$

$$error(0.9868, 0/1) = 100 \nleq 1$$

$$error(0.0133, 0/1) = 100 \nleq 1$$

$$error(0.9868, 1/1) = 1.34 \nleq 1$$

$$error(0.0133, 1/1) = 7418.80 \nleq 1$$

$$\vdots$$

$$error(0.9868, 43/44) = 0.97 \leq 1$$

$$error(0.0133, 43/44) = 7247.92 \nleq 1$$

Thus, contemporary Fe_2O_3 by Berzelius's 1819 atomic weights is transformed into $Fe_{2\times40/78}O_{1\times43/44}$, that is $Fe_{40/39}O_{129/44}$. Now, following the same procedure for $\tau = 2$, it is obtained that:

$$error(0.5077, 0/1) = 100 \nleq 2$$

$$error(0.9695, 0/1) = 100 \nleq 2$$

$$\vdots$$

$$error(0.5077, 1/2) = 1.52 \leq 2$$

$$error(0.9695, 1/2) = 48.43 \nleq 2$$

$$error(0.9868, 0/1) = 100 \nleq 2$$

$$error(0.0133, 0/1) = 100 \nleq 2$$

$$error(0.9868, 1/1) = 1.34 \leq 2$$

$$error(0.0133, 1/1) = 7418.80 \nleq 2$$

Hence, Fe_2O_3 is approximated to $Fe_{2\times1/2}O_{3\times1/1}$, with $\tau = 2$, that is FeO_3.

For $\tau = 2$ to $\tau = 20$, the coefficients for Fe and O keep being 1/2 and 1/1, respectively. Thus,

for 19 out of the 20 tolerance values, Fe_2O_3 is approximated to FeO_3, which is actually as Berzelius reported "oxidum ferricum" in 1819 (12) (page 58).

14 Similarities in the SCE of 1868 and their relationships with those of each chemist's SCE

For every chemist publishing a set of atomic weights in year y, known Reaxys substances (S_{y-1}) up to year $y-1$ (inclusive) were retrieved and the corresponding SCE P_{y-1} was obtained (see Figure 2 (main text)). Formulae of substances S_{y-1} were approximated with 20 different tolerance values (τ), each τ yielding a SCE with similarities gathered in P^{τ}_{y-1} (Section 13).

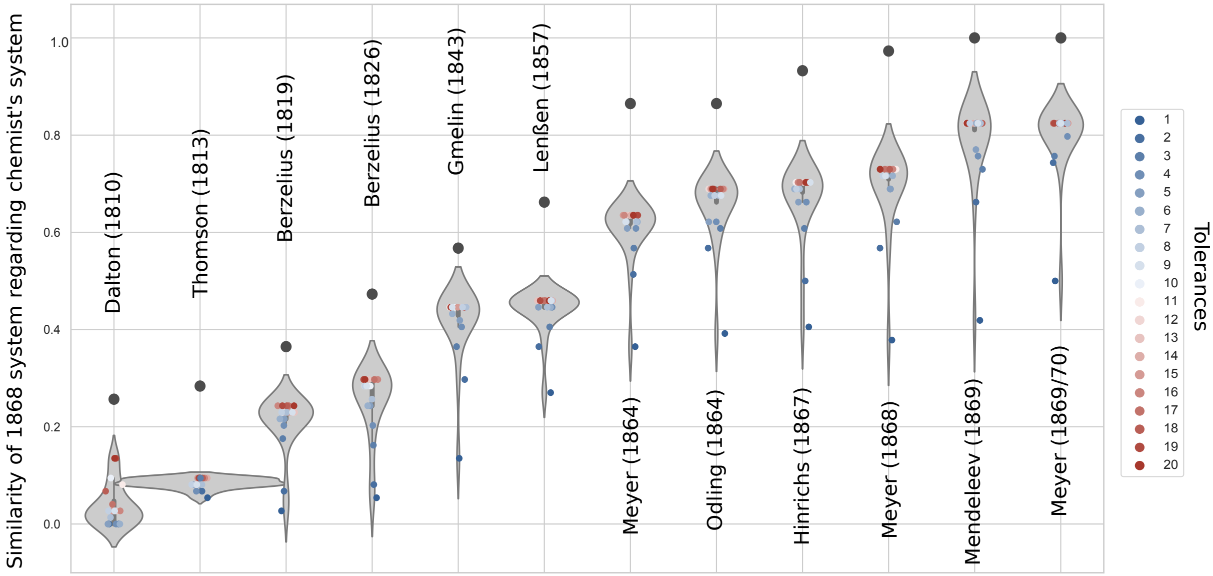

Figure S11: Fraction of 1868 similarities observed by chemist space with tolerance τ, calculated as $|P^{\tau}_{y-1} \cap P_{1868}|/|P_{1868}|$, with P_{1868} collecting the actual similarities by 1868. The 20 similarity values (coloured dots) for each chemist are gathered together in a violin plot. For the sake of comparison the similarity $|P_{y-1} \cap P_{1868}|/|P_{1868}|$ is depicted as a black dot

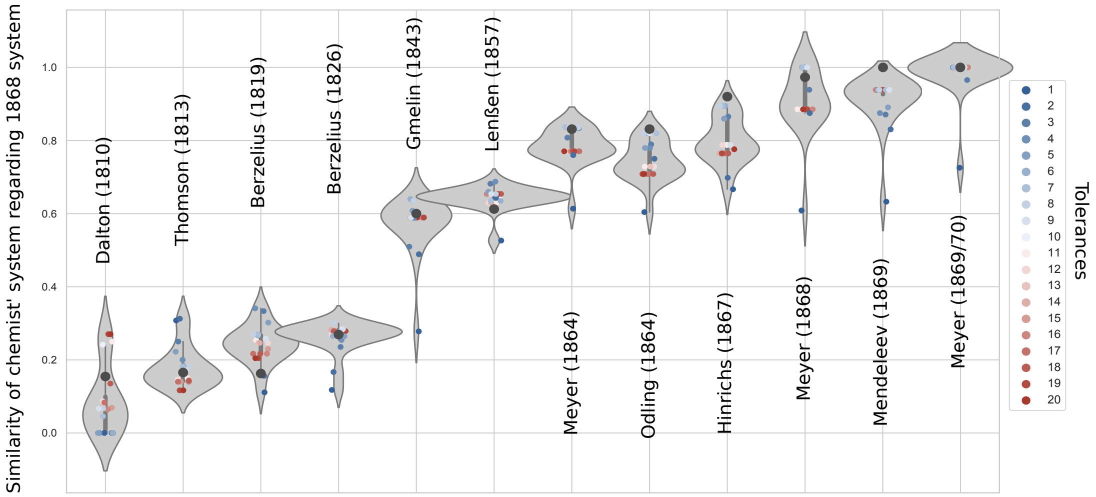

Figure S12: Fraction of similarities observed by chemist' space with tolerance τ in year $y-1$ that are observed in 1868, calculated as $|P^{\tau}_{y-1} \cap P_{1868}|/|P^{\tau}_{y-1}|$. The 20 similarity values (coloured dots) for each chemist are gathered together in a violin plot. For the sake of comparison the similarity $|P_{y-1} \cap P_{1868}|/|P_{y-1}|$ is depicted as a black dot.

According to Figure S12, differences in the systems of atomic weights appear as a major issue in the construction of SCEs during the early years of the century. For instance, in Dalton's case (1810) strong perturbations on the formulae accounting for differences in atomic weights (low tolerances) did not produce any 1868 similarity, while tiny perturbations associated with high tolerances made that about 30% of the resulting similarities matched those observed in 1868 (Figure S12). Interestingly, this is a larger similarity than that of the SCE obtained with the unperturbed chemical space (our modern formulae, black dot in Dalton's violin in Figure S12), which means that Dalton's data and assumptions regarding atomic weight could actually have been used to improve the SCE. A similar behaviour is observed for Berzelius (1819) (Figure S12). These behaviours especially occurred before 1830, as a consequence of the large number of similarities resulting from those exploratory times. For instance, the black dot in Berzelius 1819 (Figure S12) indicates that out of the 166 most similar relationships allowed by the chemical space of that time, 27 were observed in 1868. However, Berzelius' (1819) upper coloured dot (Figure S12) shows that his atomic weights (with tolerance 4) led to a system with only 44 most similar relationships and of those, 15 were found in 1868 (Interactive Information).

For some chemists, the spaces meeting their atomic weights produced rather stable fractions of similarities observed in 1868 (Figure S12). Berzelius (1826), Lenßen and Meyer (1869/70) are instances of this behaviour. Three remarkable cases, where the systems of each chemist

coincide with the available system of their times are Berzelius (1826), Gmelin and Meyer (1869/70). In these cases the black dot in Figure S12 lies about the middle of the coloured dots of these chemists. For Berzelius (1826) about 30% of his similarities matched those of 1868, for Gmelin it was about 60% and for Meyer 100%. In this latter case, for almost all perturbations of the actual 1868 space (from tolerance 5 to 20), all resulting similarities corresponded to those of the unperturbed space (Interactive Information). That is, Meyer's 1869/70 atomic weights produced the system we know was actually available by 1869, corresponding to that depicted in Figure 3c (main text), disregarding Ce, Th, La and U, which Meyer did not include. Therefore, Meyer attained 100% and 0% of true and false negatives, respectively. Meyer's 1869/70 system outperformed Mendeleev's contemporary one, as Mendeleev did not attain all the similarities of his time (his coloured dots lie below the black dot in Figure S12). This was mainly caused by Mendeleev's attempt at completeness associated to his efforts to include rare earths and In. Such a discrepancy is observed in Mendeleev's true positive (82%) and false negative rates (18%) for tolerances between 6 and 20. Disregarding Ce, Th, La and U, only In remains as the single difference between Meyer and Mendeleev's systems. For Meyer, In was similar to Al, while Mendeleev found In similar to Ce and Ce to In. The system by 1869 shows that In was actually similar to Al (Figure 3c).

References

[1] Mendeleev, D. On the relation of the properties to the atomic weights of the elements. In Jensen, W. B. (ed.) *Mendeleev on the Periodic Law: Selected Writings, 1869-1905*, chap. 1, 16–17 (Dover, New York, 2002).

[2] Coplen, T. B. & Peiser, H. S. History of the recommended atomic-weight values from 1882 to 1997: A comparison of differences from current values to the estimated uncertainties of earlier values (technical report). *Pure and Applied Chemistry* **70**, 237–257 (2019).

[3] Evans, C. *Episodes from the History of the Rare Earth Elements* (Boston : Kluwer Academic Publishers, 1996).

[4] Meyer, L. Die Natur der chemischen Elemente als Function ihrer Atomgewichte. *Ann. Chem. Pharm.* **VII Supplementband**, 354–364 (1870).

[5] Warr, W. A. Representation of chemical structures. *Wiley Interdisciplinary Reviews: Computational Molecular Science* **1**, 557–579 (2011). URL https://onlinelibrary.wiley.com/doi/abs/10.1002/wcms.36.

[6] Meyer, L. *Die modernen Theorien der Chemie und ihre Bedeutung für die chemische Statik* (Breslau: Verlag von Maruschke & Berendt, 1864).

[7] Rex, F. Zur erinnerungen an felix hoppe-seyler, lothar meyer und walter hückel. *Bausteine zur Tübinger Universitätsgeschichte* **8**, 103–130 (1997).

[8] Mendeleev, D. On the correlation between the properties of the elements and their atomic weights. In Jensen, W. B. (ed.) *Mendeleev on the Periodic Law: Selected Writings, 1869-1905*, chap. 2, 18–37 (Dover, New York, 2002).

[9] Lothar Meyer's Pathway to Periodicity. *Ambix* **66**, 265–302 (2019). URL https://doi.org/10.1080/00026980.2019.1677976. PMID: 31645216, https://doi.org/10.1080/00026980.2019.1677976.

[10] Dalton, J. *A New System of Chemical Philosophy, part 2* (Manchester, 1810).

[11] Thomson, T. On the Daltonian Theory of Definite Proportions in Chemical Combinations. *Annals of Philosophy* **2**, 32–52 (1813).

[12] Berzelius, J. J. *Essai sur la théorie des proportions chimiques et sur l'influence chimique de l'électricité* (Paris, 1819).

[13] Berzelius, J. J. Ueber die Bestimmung der relativen Anzahl von einfachen Atomen in chemischen Verbindungen. *Annalen der Physik* **83**, 397–416 (1826). URL https://onlinelibrary.wiley.com/doi/abs/10.1002/andp.18260830802. https://onlinelibrary.wiley.com/doi/pdf/10.1002/andp.18260830802.

[14] Berzelius, J. J. Ueber die Bestimmung der relativen Anzahl von einfachen Atomen in chemischen Verbindungen. *Annalen der Physik* **84**, 1–24 (1826). URL

https://onlinelibrary.wiley.com/doi/abs/10.1002/andp.18260840902.
https://onlinelibrary.wiley.com/doi/pdf/10.1002/andp.18260840902.

[15] Berzelius, J. J. Ueber die Bestimmung der relativen Anzahl von einfachen Atomen in chemischen Verbindungen. *Annalen der Physik* **84**, 177–190 (1826). URL https://onlinelibrary.wiley.com/doi/abs/10.1002/andp.18260841006. https://onlinelibrary.wiley.com/doi/pdf/10.1002/andp.18260841006.

[16] Gmelin, L. *Handbuch der chemie* (Karl Winter, Heidelberg, 1843). URL //catalog.hathitrust.org/Record/010055907. 8 v. in 9.

[17] Lenßen, E. Ueber die Gruppirung der Elemente nach ihrem chemisch - physikalischen Character. *Justus Liebigs Annalen der Chemie* **103**, 121–131 (1857). URL https://chemistry-europe.onlinelibrary.wiley.com/doi/abs/10.1002/jlac.18571030202. https://chemistry-europe.onlinelibrary.wiley.com/doi/pdf/10.1002/jlac.18571030202.

[18] Odling, W. On the proportional numbers of the elements. *Quarterly Journal of Science* **1**, 642–648 (1864).

[19] Hinrichs, G. *Atomechanik* (Iowa, 1867).

[20] Meija, J. *et al.* Atomic weights of the elements 2013 (iupac technical report). *Pure and Applied Chemistry* **88**, 265 – 291 (01 Mar. 2016). URL https://www.degruyter.com/view/journals/pac/88/3/article-p265.xml.

[21] Farey, J. On a curious property of vulgar fractions. *The Philosophical Magazine and Journal* **47**, 385–386 (1816).

[22] Vardi, I. *Computational Recreations in Mathematica*. The advanced book program (Addison-Wesley, 1991). URL https://books.google.de/books?id=29hQAAAAMAAJ.
